# Defects in auxiliary fuel oxidation and mitochondrial pyruvate transport mark transition to overt heart failure in Tgαq*44 mice

**DOI:** 10.1186/s12967-026-07883-y

**Published:** 2026-03-03

**Authors:** Mariola Olkowicz, Agata Jedrzejewska, Urszula Tyrankiewicz, Filip Aleksander Fedak, Piotr Berkowicz, Grzegorz Kwiatkowski, Hernando Rosales-Solano, Kanchan Sinha Roy, Agnieszka Karas, Oliwia Krol, Marta Tomczyk, Ryszard T. Smolenski, Janusz Pawliszyn, Stefan Chlopicki

**Affiliations:** 1 Jagiellonian Centre for Experimental Therapeutics (JCET), Jagiellonian University, Bobrzynskiego 14, Krakow, 30-348 Poland; 2 Department of Biopharmaceutics and Pharmacodynamics, Medical University of Gdansk, Hallera 107, Gdansk, 80-416 Poland; 3 Department of Biochemistry, Medical University of Gdansk, Debinki 1, Gdansk, 80-211 Poland; 4 Doctoral School of Exact and Natural Sciences, Jagiellonian University, Lojasiewicza 11, Krakow, 30-348 Poland; 5 Department of Chemistry, University of Waterloo, 200 University Avenue West, Waterloo, ON N2L 3G1 Canada; 6 Department of Pharmacology, Jagiellonian University Medical College, Grzegorzecka 16, Krakow, 31-531 Poland

**Keywords:** Heart failure, Energy metabolism, Non-targeted proteomics, Mitochondrial pyruvate uptake, Branched-chain amino acid accumulation, Ketone oxidation

## Abstract

**Background:**

Defective catabolism of alternative and glucose-sparing fuel sources has recently been implicated in the development of cardiovascular and metabolic diseases, including heart failure (HF), but the molecular mechanisms and a causal relationship linking them to altered glucose metabolism are unknown.

**Methods:**

Herein, alterations in cardiac protein expression in an established HF model (Tgαq*44 mice) were explored at different ages (4−14 months) in relation to changes in energy substrate preference, high-energy phosphate metabolism, and a snapshot of plasma metabolites. A small cohort of HF cases (*n* = 20) and non-failing controls (*n* = 18) was also used to confirm translational value of the findings.

**Results:**

The progression of HF in Tgαq*44 mice was characterised by an increased reliance on glucose along with reduced mitochondrial oxidative metabolism that was associated with impaired MPC (mitochondrial pyruvate carrier)−mediated pyruvate utilisation and redirection of glycolytic intermediates into the hexose monophosphate shunt, glycogenesis, and serine biosynthetic pathway. Defects in fatty acid (FA), pyruvate, branched-chain amino acid (BCAA), and ketone body (KB) oxidation, alongside prominent elevation of lactate, represented major features of altered cardiac metabolism in end-stage HF. Chronic accumulation of BCAAs next to suppressed KB and disrupted glucose oxidation were also found in patients with advanced HF, underlying the clinical relevance of the observed alterations.

**Conclusion:**

This study provides the comprehensive pattern of metabolic evolution of HF, highlighting several possible avenues to rescue from the HF-prone phenotype, such as promoting BCAA and KB catabolism, or normalising glucose utilisation by overexpressing MPC.

**Supplementary Information:**

The online version contains supplementary material available at 10.1186/s12967-026-07883-y.

## Background

Myocardial energy metabolism is intricately linked to cardiac function, and its perturbations result in an energy-starved heart and, consequently, the development of contractile dysfunction [1, 2]. This is specifically seen in heart failure (HF), where metabolic inflexibility and energetic deficit are associated with maladaptive metabolic remodelling. The metabolic changes that occur in HF are complex and distinct for different aetiologies/subtypes of HF [3]. However, investigations on altered substrate utilisation in numerous single-gene transgenic or gain- and loss-of-function animal models are challenged in their ability to accurately recapitulate disease aetiology, duration, and severity, and as such have provided conflicting information, thus, further examinations integrating multiple levels of analysis: from molecular mechanisms to clinical outcomes are warranted. In line with the above, the failing heart undergoes dramatic alterations in energy substrate utilisation and overall metabolic profile, which cannot only lead to a disturbed balance of cardiac energy metabolism but also to an overall compromised ATP production primarily from mitochondrial oxidative phosphorylation [4–6]. The relative contribution of the different fuel sources for mitochondrial ATP production varies depending on the type of HF [5, 6]. While a common metabolic defect in all forms of HF is a decrease in mitochondrial oxidation of pyruvate originating from glucose (i.e., glucose oxidation) that occurs regardless of whether glycolysis is increased, observations on alterations in fatty acid (FA) oxidation in the failing heart are less conclusive. These discrepant results might be to a large extent explained by differences in disease aetiology, co-morbidities involved with HF, differences in transcriptional and post-translational regulation of energy metabolism, and/or the severity of the disease [5], in addition to contradictions in literature arising from methodological differences [7]. Nonetheless, the precise alteration of each metabolic pathway in HF has yet to be established.

Adding to the above, although mitochondrial FA and carbohydrate oxidation constitute the major source of ATP production in the heart, it is becoming increasingly clear that catabolism of other (auxiliary) energy fuels, such as ketones and branched chain amino acids (BCAAs), can also contribute to energy production [3, 8]. Although these substrates contribute less to overall energy production, perturbations in cardiac ketone and BCAA metabolism may also impact the severity of HF through alterations in cellular signalling [9–11]. Notably, in contrast to ketones that have recently appeared as a super fuel being oxidised by the heart in preference to FAs and glucose, which potentially may improve cardiac function in the failing heart in addition to correction of cardiac efficiency, the role of amino acid (AA) metabolism in the diseased heart has rarely been studied [12–15]. Nonetheless, it is essential to verify to what extent alterations in these substrate fuels’ utilisation contribute to the progression of chronic HF from early compensated to late decompensated HF.

Although small animal models can mimic various aspects of the pathogenesis of human HF, including metabolic perturbations [16–18], most of these models develop decompensated HF in a relatively short period after the primary insult, thus, the information on the timing of metabolic alterations and the sequential relationship between structural/functional and metabolic processes, in particular in the transition phase from asymptomatic to overt HF, is lacking. In this context, Tgαq*44 mice (maintained in the FVB background) with targeted overexpression of HA-tagged, constitutively activated Gαq (HAαq*) protein in cardiomyocytes mimicking excessive neurohormonal stimulation may represent a unique model of chronic HF, relevant to human pathophysiology, as it is characterised by a prolonged course of HF progression [19]. Slowly developing HF (in this model) is featured by progressive decline of cardiac performance with concomitant cardiac fibrosis, significantly impaired physical activity, and endothelial dysfunction in aorta, coronary, cerebral and liver circulation [20–29]. Additionally, our preliminary studies revealed reversion to foetal gene expression with a shift towards glycolysis and less efficient glucose oxidation, impairment of FA oxidation (FAO) capacity, and insufficient compensatory increase in FAO at the age of 12 months [30] that seemed to be partially improved by physical activity of moderate intensity [31]. However, myocardial intermediary metabolism that extends beyond FAs and carbohydrates has not been explored with this model until now; despite numerous papers describing various aspects of the pathophysiology of chronic HF in Tgαq*44 mice [20–29, 32–34].

In the present study, we sought to elucidate the pattern and time-course of alterations in bioenergetic metabolism in the unique murine model of slowly developing chronic HF (Tgαq*44). By integrating proteomic, metabolomic, and functional data, metabolism-related disease pathways associated with the progression of HF were identified, validated with clinical data, and ultimately pointed to defective auxiliary fuel catabolism along with impaired mitochondrial pyruvate uptake that featured metabolic remodelling and HF progression.

## Methods

### Animals

Tgαq*44 mice, initially developed by Mende et al. [19], and wild-type control mice (FVB), were bred in the Institute of Experimental and Clinical Medicine of the Polish Academy of Sciences (Warsaw). Animals were maintained under specific pathogen-free conditions in isolated ventilated cages, in a room with controlled environment (22–25 °C, 65–75% humidity, and a 12-h light/dark cycle). Water and food (standard laboratory diet) were supplied ad libitum. Due to a higher level of aggression in ageing males than females, which could significantly interfere with functional/behavioural assessments, all experiments were performed on female Tgαq*44 and age-matched female FVB mice at the ages of 4, 8, 10, 12, and 14 months, representing three distinct stages of HF development: (1) early compensated stage of HF (4 months); (2) the transition phase to overt HF (8−10 months); and (3) decompensated, end-stage HF (12−14 months) [27]. Tgαq*44 mice that mirror the HFrEF (HF with reduced ejection fraction) phenotype in humans exhibit delayed progression of HF with cardiac systolic and diastolic functions which begin to deteriorate at 4–6 months of age, and end-stage cardiac decompensation that usually occurs at ∼12–14 months [20, 27] that is also associated with impaired right ventricular function and liver congestion [29] adding another aspect of HF progression, relevant to human pathophysiology, in this model of chronic HF.

Tgαq*44 and FVB mice were euthanised with ketamine (100 mg/kg) and xylazine (10 mg/kg) administered intraperitoneally. The time from induction of anaesthesia to the heart collection was maintained for up to 10 min to limit the potential effect of anaesthesia and post-mortem variables on metabolic outcome. All procedures involving animals were approved by the 2nd Local Ethical Committee on Animal Testing in the Institute of Pharmacology, Polish Academy of Sciences (Krakow, Poland; permit no. 190/2020), and conducted in accordance with the guidelines of Directive 2010/63/EU of the European Parliament on the protection of animals used for scientific purposes.

### Echocardiographic assessment of left ventricular function

**Fig. 1:**
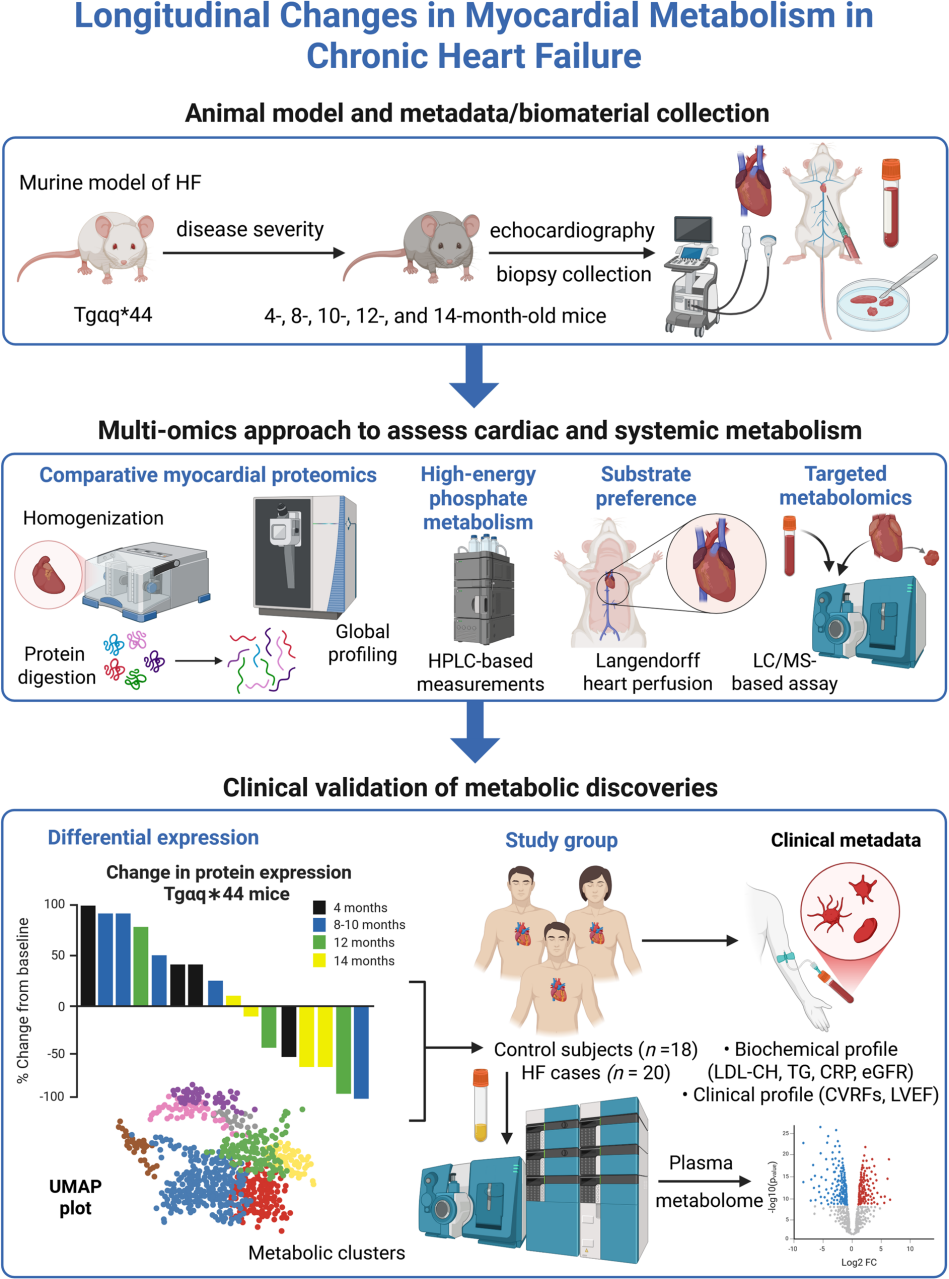
Overview of the study design. Multi-omics flow diagram demonstrating the process of discovering and clinically validating protein/metabolite marker panel. This figure was created with BioRender.com

Echocardiographic examinations were performed with a Vinno 6 Lab high-resolution ultrasound imaging system (VINNO Technology, Suzhou, China) equipped with a 21-MHz linear probe and tissue Doppler. Under ketamine/xylazine anaesthesia, the chest hair was removed and mice were placed on a heating platform to sustain body temperature at 37 °C. The probe was placed over the anterior chest wall and directed to the ascending aorta in 2D mode. Afterwards, the mode was switched to Doppler flow velocity. Left ventricular structure and systolic function were assessed in M-mode with 1–3 replicates recorded for each mouse. A visual representation of the research design is shown in Fig. 1.

### Comparative cardiac proteome profiling

Tgαq*44 and FVB mice ranging from 4−14 months of age were sacrificed and subjected to transcardial perfusion with saline to flush out the blood [35]. The saline perfusion was continued until the fluid exiting the right auricle was clear of blood. LVs were dissected into saline solution, snap-frozen in liquid nitrogen, and stored at − 80 °C. To perform the protein extraction, hearts were thawed on ice and resuspended in lysis buffer (6 M urea, 50 mM ammonium bicarbonate (ABC); 1/8, w/v) supplemented with protease inhibitors. Subsequently, samples were subjected to homogenisation and centrifuged at 10,000 x g for 5 min at room temperature to remove insoluble debris. Protein concentrations were determined using the Bradford method. Protein samples (150 µg) were reduced with 5 mM dithiothreitol (DTT) at 56 °C for 30 min, then alkylated with 15 mM iodoacetamide (IAA) at room temperature in the dark for 45 min. Prior to digestion, the samples were diluted in ABC buffer to a final urea concentration of 1 M. Next, the samples were subjected to digestion with Lys-C (LabJOT, Warsaw) for 2 h and trypsin (Promega, Warsaw) overnight at 37 °C at a 1:50 enzyme: protein ratio. The resulting peptide samples were acidified and desalted on Pierce Peptide Desalting Spin Columns (Merck, Poznan) and later on concentrated through freeze drying. Each sample (15 µg) was reconstituted with 0.1% formic acid prior to label-free mass spectrometric analysis, as previously described [36]. Exponentially modified protein abundance index (emPAI) was used for estimation of absolute protein amount in complex samples employing the number of sequenced peptides per protein [37].

### Cardiac substrate preference at rest and under dobutamine stress in Langendorff-perfused hearts

Metabolic flux assays were carried out with isolated Langendorff-perfused hearts from 4- and 14-month-old FVB and Tgαq*44 mice (*n* = 5–10/group) [38]. Hearts were quickly excised and cannulated through the aorta. Next, the cannulated heart was connected to a perfusion column apparatus maintained at 37 °C using a temperature-controlled water bath. Hearts were perfused retrogradely for 45 min with Krebs–Henseleit buffer (15 min stabilisation period + 30 min continuous perfusion) with the following substrates (mM): 5 ᴅ-glucose, 1 sodium lactate, 0.5 sodium pyruvate, 0.25 L-leucine, 0.25 L-isoleucine, 0.3 L-valine, 0.25 L-glutamate, 0.5 sodium octanoate, and 0.4 β-hydroxybutyrate (β-OHB) supplemented with 0.05 mM L-carnitine. Dobutamine (Dbn, Merck, Poland) stress was induced by the infusion of the drug (Dbn) at 10 nmol/10 µL, which produced increases in cardiac contractility and output. Coronary effluent samples were collected during the 15-minute stabilisation period before brief ischaemia (30-s coronary occlusion induced by clamping the inflow tubing), at reactive hyperaemia, and immediately before and after injection of the bolus of Dbn. Aliquots were stored at − 80 °C until analysis with a mass spectrometry-based platform to profile intermediary metabolites [39]. Additionally, at the end of the perfusion, hearts were snap frozen with liquid nitrogen and stored at − 80 °C until processing for biochemical analysis.

### Solid-phase microextraction (SPME) technology for metabolite analysis

The metabolites recovered in the coronary effluents were extracted with an innovative and greener sample preparation technique – SPME which integrates sampling, extraction, and pre-concentration into a single step [40, 41]. SPME is widely recognised for its applicability across diverse biological matrices, including in vivo applications, due to its simplified, solvent-minimised workflow. It has been successfully applied in the analysis of a broad range of metabolites in a variety of matrices, which demonstrated its utility in metabolomics studies [41–43]. SPME offers several important advantages for metabolomics investigations, namely: (1) a solvent-free, streamlined workflow that uses coated devices of various geometries and dimensions, with matrix-compatible, tuneable extraction phases; (2) the ability to minimise matrix effects by decoupling sampling from sample complexity; (3) an improved enrichment/sensitivity (detection) of low-abundance species; and (4) the suitability for automation. The thin film (TF) geometry (format) of SPME was applied in this study to enhance the sensitivity of the technique without compromising sampling time, owing to its expanded surface area, which offers more active/accessible sites for analyte diffusion and adsorption, resulting in more efficient extraction. TF-SPME blades with a matrix-compatible HLB/PAN coating (length – 10 mm and thickness – 45 μm) were made in-house at the University of Waterloo, as detailed elsewhere [44]. The general procedure for the use of the TFME devices involved: (1) preconditioning with MeOH/H_2_O (1/1, v/v) for 30 min followed by a brief rinsing step (H_2_O, 5s) to activate the coating material and prepare it for interaction with the sample matrix; (2) 30-min extraction by directly immersing the coating into the complex matrix; (3) a quick washing step with pure water to remove materials loosely attached to the coating surface; and (4) a 30-min solvent desorption step with ACN/H_2_O (8/2, v/v). Collected extracts were further submitted to liquid chromatography-mass spectrometry (LC/MS) analysis according to the targeted metabolomics protocol detailed elsewhere [39]. Briefly, bioenergetic phenotyping of isolated perfused hearts was attained with targeted LC-MRM/MS-based assay involving over 100 analytes related with energy-generation processes (i.e., amino acids (AAs), nucleosides, organic acids (OAs) and carbohydrates as well as acylcarnitines (ACs) and bacteria-derived metabolites). All the separations/detections for metabolomics were performed on the UFLC system (Shimadzu, Japan) coupled with QTRAP hybrid triple quadrupole mass spectrometer (5500, AB Sciex, Framingham, MA, USA). The metabolites were ionised by a TurboV™ heated electrospray ionisation source and scheduled MRM (multiple reaction monitoring) was conducted in both positive and negative modes.

### High-energy phosphate metabolism in the failing heart

Cardiac high-energy phosphates and related nucleotides, including ATP, ADP, AMP, phosphocreatine (PCr), creatine, NAD^+^, NADH (as adenosine diphosphate ribose − ADPR), NADP^+^, GTP, GDP, GMP, and IMP were measured using high-pressure liquid chromatography (HPLC), as previously described [45]. Briefly, mouse hearts were dissected by freeze-clamping in liquid nitrogen while the animals were anaesthetised with a ketamine/xylazine mixture (50 mg/kg and 5 mg/kg, respectively) and artificially ventilated. The frozen whole hearts were subjected to freeze-drying for 24 h and then extracted with 0.4 M HClO_4_ at a 1:25 ratio using a glass homogeniser. Following centrifugation at 14,000 rpm for 10 min at 4 °C, the supernatants were neutralised with 2 M KOH. Aliquots from the second round of centrifugation were retained for HPLC-based analysis. On the basis of the results collected, specific energetic indicators were computed. The cardiac ATP/ADP ratio and adenylate energy charge (AEC) were utilised as markers of cellular energetic equilibrium and as proxies for phosphorylation potential [46, 47]. In turn, the phosphocreatine (PCr) and creatine acted as shuttle molecules between the sites of ATP production and hydrolysis, and the total PCr pool served as the primary energy reserve in the heart [48].

### Targeted metabolomics and the aberrant energy status in blood

Quantitative targeted metabolite profiling of individual AAs, OAs, including lactate, pyruvate, succinate, fumarate, malate, α-ketoglutarate, citrate, β-OHB, and ACs was performed using a validated, optimised LC/MS−based protocol, as reported [39, 49]. Plasma samples (50 µL) were spiked with stable isotope-labelled internal standards followed by extraction with ice-cold methanol (150 µL) to arrest cellular metabolism and optimise the stability of the metabolites. The instrumental analysis of methanolic extracts was carried out on a triple quadrupole QTRAP5500 mass spectrometer (AB SCIEX, Framingham, MA, USA) interfaced to a Shimadzu UFLC system consisting of a binary pump, a column oven, and an autosampler. Reversed-phase or HILIC (for ACs) chromatography was applied for separation and the scheduled multiple reaction monitoring (MRM) algorithm was used to ensure robust detection of the large panel of analytes in a single injection. Data were analysed using the Quantitation Wizard within Analyst software (v. 1.6, AB Sciex Ltd., Warrington, Cheshire, UK). In addition, the plasma concentration of FAs was measured using a non-esterified FA (NEFA) assay kit (Merck, Poznan, Poland) according to the manufacturer’s protocol.

### Study population and clinical samples

Patients with an EF < 50% at the time of HF diagnosis were included (*n* = 25) and underwent metabolomic profiling of stored plasma. Among these patients, the analysis was restricted to those with complete echocardiographic/clinical data and information on N-terminal pro-B-type natriuretic peptide (NT-pro-BNP) levels. This group (*n* = 20) was used for testing the association of metabolites with demographic and clinical characteristics. Control subjects (*n* = 18) within a similar distribution of gender, age, and with no reported cardiac phenotype were also enrolled. Informed consent was obtained from all patients. The study was designed and carried out in accordance with the principles of the Declaration of Helsinki and with approval from the Bioethics Committee at the Medical University of Gdansk.

### Statistical analysis

Quantitative data were plotted using GraphPad Prism v. 10.2 with results expressed as the mean ± SD or as the median and interquartile range, as appropriate. The reported data were derived from multiple independent biological replicates with the sample sizes specified in the figure or figure legends. For comparison of two independent groups, the parametric unpaired t-test or Mann-Whitney test (where the distribution of data points failed the normality test) was used. When comparing more than two groups, one-way/two-way ANOVA followed by Tukey’s test for post-hoc analysis or Kruskal-Wallis with Dunn's multiple comparison test was applied. Statistical significance was determined at a threshold of *p* < 0.05.

## Results

### Alterations in systolic cardiac function in HF in Tgαq*44 mice

**Fig. 2:**
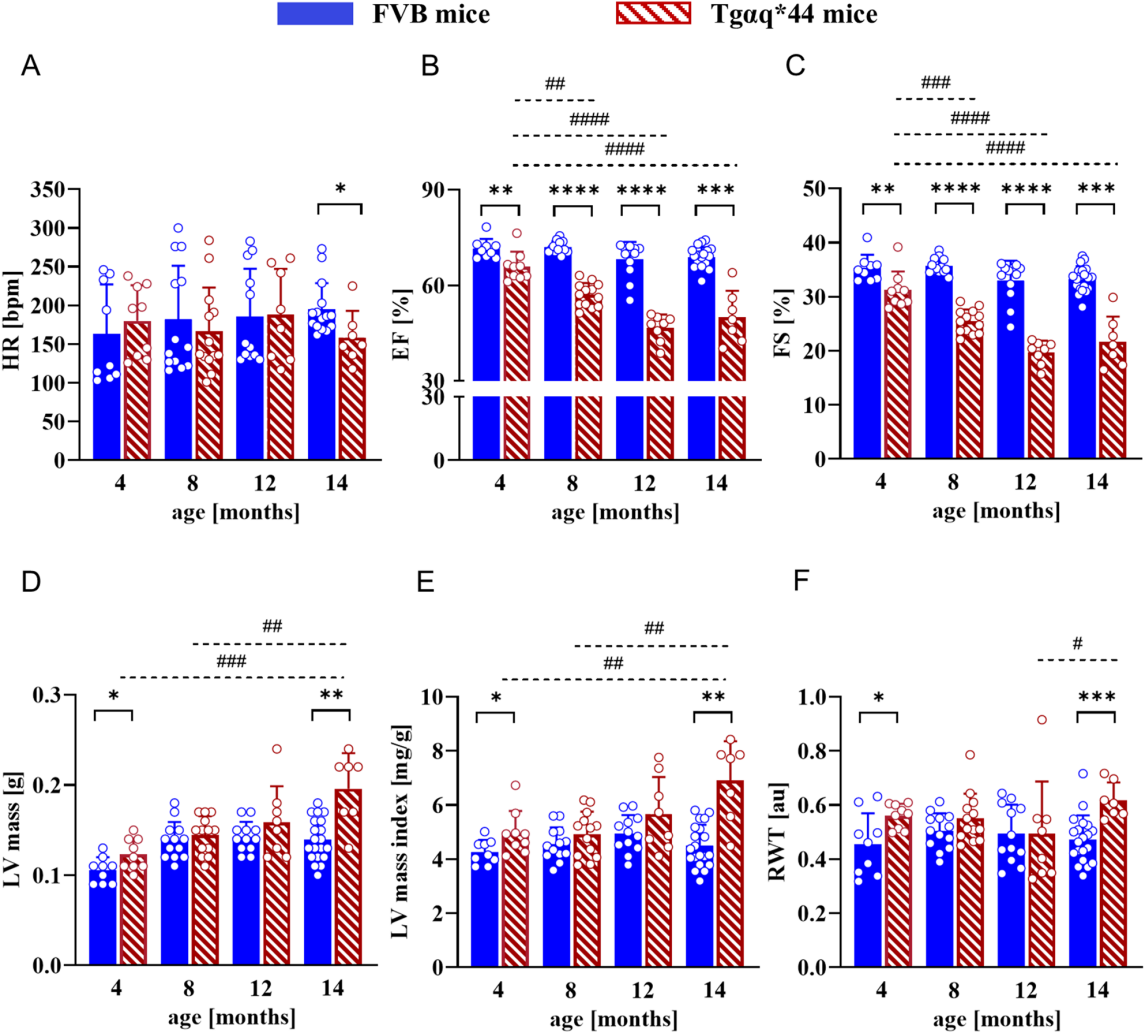
Haemodynamic parameters of cardiac function in 4-, 8-, 12- and 14-month-old FVB and Tgαq*44 mice. The data are presented as the mean ± SD; *n* = 7‒20; * *p* < 0.05, ** *p* < 0.01, *** *p* < 0.001, **** *p* < 0.0001 for Tgαq*44 mice vs. age-matched FVB mice (Student’s t test or Mann–Whitney test); # *p* < 0.05, ## *p* < 0.01, ### *p* < 0.001, #### *p* < 0.0001 for 14-, 12-, 8- vs. 4-month-old Tgαq*44 mice (one-way ANOVA with post hoc Tukey’s test). HR, heart rate; LV, left ventricle; (LV)EF, left ventricular ejection fraction; (LV)FS, left ventricular fractional shortening; RWT, relative wall thickness. LV mass index (LVMI) was calculated by dividing LV mass by body weight, while RWT was computed as 2 times posterior wall thickness (PWT) divided by the LV diastolic diameter

Echocardiographic measurements demonstrated that even in young 4-month-old Tgαq*44 mice, global heart function was impaired in the systolic phase compared to 4-month-old FVB mice (Fig. 2, Sup. Table 1 (Additional file 1)), but these lesions were rather modest. In turn, 12-/14-month-old Tgαq*44 mice displayed severe systolic cardiac function impairment compared to 4-month-old Tgαq*44 mice and age-matched FVB mice, as evidenced by greatly decreased left ventricular ejection fraction (EF) and fractional shortening (FS). In addition, LV wall thickening was found in older (14-month-old) Tgαq*44 mice vs. age-matched FVB mice, as characterised by higher LV mass and relative wall thickness (RWT). Of note, left-sided chronic HF developed by Tgαq*44 mice, as shown in previous work [20, 21, 27] and corroborated in the present study, promoted right-ventricular dysfunction. Precisely, as reported by Wojnar-Lason et al. [29], in 4-month-old Tgαq*44 mice, the RV ejection fraction was significantly attenuated and further deteriorated in 12-month-old Tgαq*44 mice with concomitant impairment in stroke volume and cardiac output. In addition, the impairment of RV function in Tgαq*44 mice was accompanied by evident increase in RV mass for both 4- and 12-month-old Tgαq*44 when compared against age-matched control (FVB) mice. Consistent with the above, macroscopic heart enlargement was observed specifically in older (12‒14-month-old) Tgαq*44 mice but the increased heart mass of Tgαq*44 mice compared to FVB mice may be also caused by a significant increase in atrial mass as evidenced by Berkowicz et al. [27].

### Disturbed high-energy phosphate metabolism occurs prior to the onset of HF

**Fig. 3:**
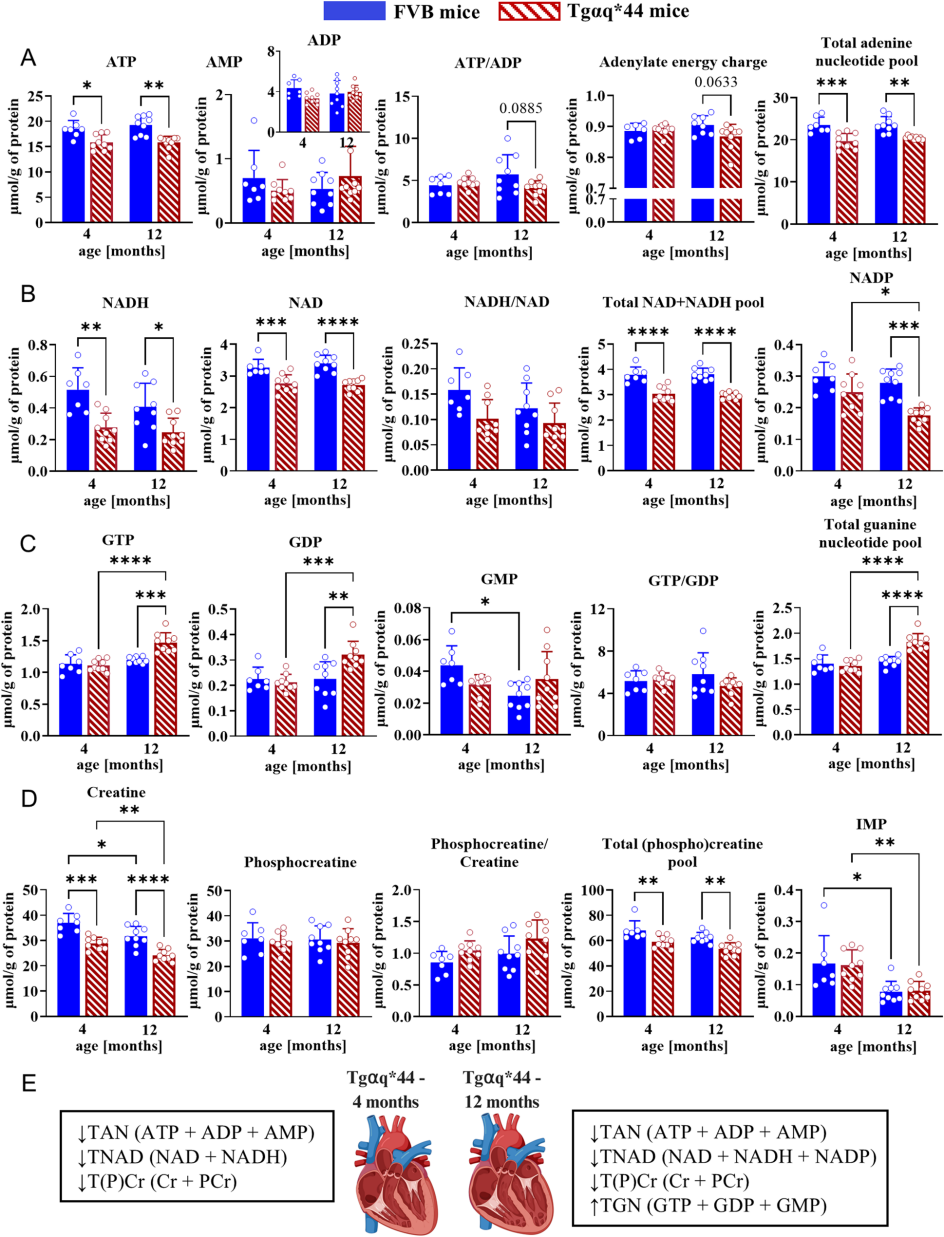
Impaired bioenergetics of the failing heart. (**A‒D**) Absolute concentrations (µmol/g of protein) of high-energy phosphate metabolites in normal and failing murine heart measured with a high performance liquid chromatography-based assay. The data (for *n* = 7‒9 samples in each group) are shown as the mean ± SD and were compared with a 2-way ANOVA with a Tukey post hoc test; * *p* < 0.05, ** *p* < 0.01, *** *p* < 0.001, **** *p* < 0.0001. (**E**) The energetic status of the heart in 4- and 12-month-old Tgαq*44 mice compared to age-matched FVB wild-types. *Abbreviations*: AEC, adenylate energy charge; TAN, total adenine nucleotide (pool); TGN, total guanine nucleotide (pool); T(P)Cr, total (phospho)creatine (pool). The “energy charge” of the adenylate system was defined as (ATP + ½ ADP)/(AMP + ADP + ATP)

Early impairment of cardiac function in 4-month-old Tgαq*44 mice was associated with a decline in cardiac high-energy phosphate metabolism (Fig. 3), manifested by a fall in ATP and total adenine nucleotide pool as well as creatine and total creatine pool. Interestingly, the ATP/ADP ratio and adenylate energy charge were not compromised in 4-month-old Tgαq*44 mice compared with age-matched FVB mice, but both were apparently lower in 12-month-old Tgαq*44 in comparison to FVB mice (although this change fell slightly below *p*-value threshold). Decreased intracellular NAD^+^ availability and total NAD pool were evident in both 4- and 12-month-old Tgαq*44 mice. In contrast, tissue levels of NADP^+^ declined exclusively in 12-month-old Tgαq*44 animals. Intriguingly, an increase in cardiac GTP level was clearly identified in 12-month-old but was not altered in 4-month-old Tgαq*44 mice. This may reflect compensatory synthesis of nucleoside triphosphates other than ATP to boost aberrant energy metabolism and/or to support processes such as protein and nucleic acid synthesis and signalling in end-stage HF. Collectively, significant alterations in the high-energy phosphate pool were observed in both 4- and 12-month-old Tgαq*44 mice, although apparently they were not accompanied by impaired energy equilibrium, particularly in younger mice. In the early phase of HF, the preservation of energetic indicators (of cellular equilibrium) despite reduced adenylate and creatine pools suggests partial compensation, likely maintained at the expense of reduced energetic reserve. These findings suggest that energy transfer via the creatine kinase system remains initially and functionally preserved at this stage. At 12 months, the apparent decline in ATP/ADP ratio and energy charge is more consistent with a transition from compensation toward energetic decompensation in end-stage HF. Continued depletion of creatine pool may further compromise contractile function by obstructing the energy shuttle essential for efficient phosphate transfer.

### Comparative temporal analysis of protein dynamics in cardiac remodelling

**Fig. 4:**
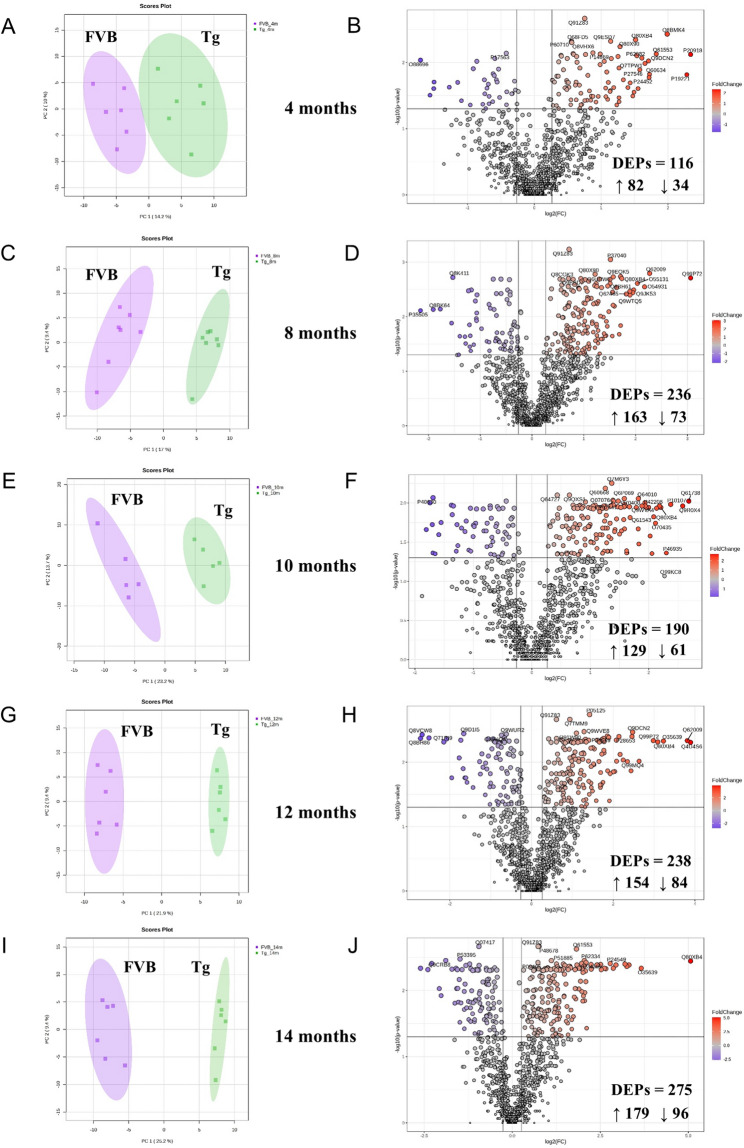
Identification of proteins differentially expressed with age (alongside the trajectory of heart failure) in left ventricle tissue lysates. (**A**, **C**, **E**, **G**, and **I**) PCA score plots of PC1 and PC2, indicating a degree of natural separation between the Tgαq*44 (green) and control (purple) groups, as well as a degree of inter-individual variation within each group. (**B**, **D**, **F**, **H**, and **J**) Differentially expressed proteins (DEPs) between Tgαq*44 and wild-type mice (FVB). Volcano plots presenting the distribution of DEPs between the studied groups: (**B**) 4-month-old, (**D**) 8-month-old, (**F**) 10-month-old, (**H**) 12-month-old, and (**J**) 14-month-old animals. The X-axis represents the log2-transformed FC (fold change), and the Y-axis represents the ‒log10-transformed *p*-value. Significantly upregulated proteins are displayed as red dots and down-regulated proteins as blue dots (*p* value < 0.05, and fold change (Tgαq*44 /Ctrl) ≥ 1.2 or ≤ 0.8). The vertical line represents a boundary between the differentially and non-differentially expressed proteins, and the horizontal line corresponds to a *p*-value equal to 0.05. For comparison of two independent groups (*n* = 6‒7/group), the Mann-Whitney test was used

To characterise longitudinally alterations in the expression pattern of metabolic enzymes in the heart of Tgαq*44 mice (compared with FVB mice), the animals were sacrificed at 4, 8, 10, 12, and 14 months of age representing different stages of HF for in-depth proteomics research. The number of Tgαq*44 and control mice was balanced, with 6–7 samples analysed at each time point (Sup. Figure 1 (Additional file 1)). Principal component analysis (Fig. 4A, C, E, G, and I) of the collected dataset demonstrated that the transgenic group was different from the control group, irrespective of age, and this feature was most pronounced in the end-stage HF. Further, we focused on the identification of up- and down-regulation of spot intensities. To take both statistical significance and biological relevance into consideration, the cut-off for differentially expressed proteins (DEPs) was set at an FDR-adjusted *p*-value (also called the *q*-value) of 0.05, with a fold change ≥1.2 or ≤0.8. DEPs identified with these criteria gradually increased in number with HF development (Fig. 4B, D, F, H, and J; and Sup. Tables 2–6 (Additional file 1)). Specifically, compared with the control group, in the Tgαq*44 mice at 4 months 116 DEPs were identified, while at 8 and 10 months – 236 and 190 DEPs were recognised, respectively (Sup. Tables 2–4 (Additional file 1)). In turn, in the established HF – 238 and 275 DEPs were found at 12 and 14 months, respectively (Sup. Tables 5–6 (Additional file 1)). It is important to note, however, that there were more proteins that displayed an increase than a decrease owing to HF. Protein groups related to structural reorganisation generally presented an upward trend, while those linked to cardiac energy substrate metabolism demonstrated rather a decreasing trend (Fig. 4 and Sup. Tables 2–6 (Additional file 1)).

**Fig. 5:**
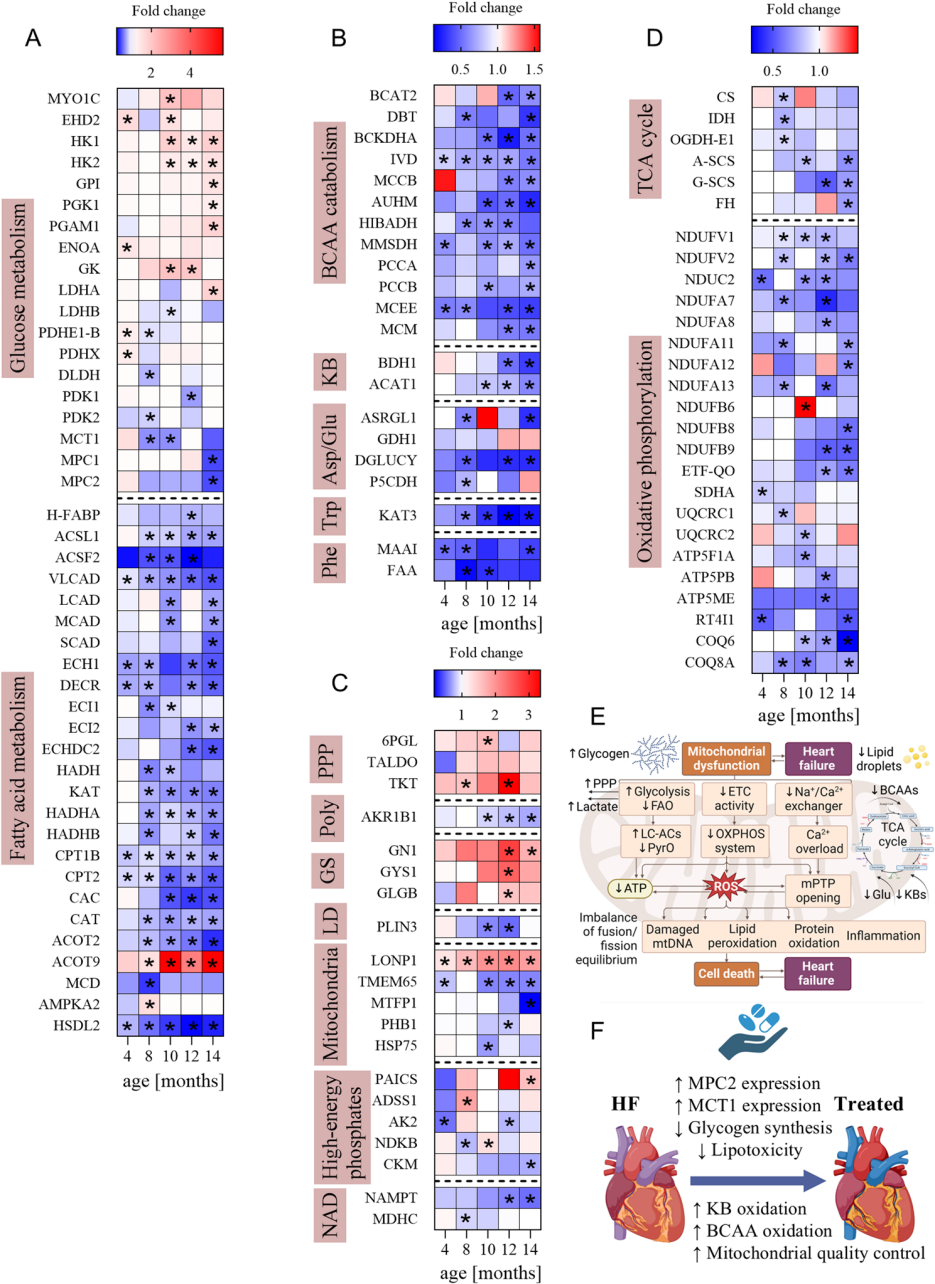
Myocardial tissue-dependent changes in the proteome during advanced HF development. (**A**‒**D**) The heatmaps showing the relative protein expression levels of metabolic proteins differentially expressed between Tgαq*44 and wild-type mice. The colour scheme shows the fold changes related to the average of age-matched control mice. The asterisk denotes a statistically significant difference. (**E**) Major metabolic alterations occurring in cardiac pathologic conditions under maladaptive remodelling of the failing myocardium (in the context of presented results). (**F**) Potential therapeutic approaches for targeting impaired cardiac energy metabolism in HF (suggested based on proteomics results). (**E**‒**F**) The graphics were created with a licensed version of Biorender.com. Abbreviations: Asp, aspartate; BCAA(s), branched-chain amino acid(s); ETC, electron transport chain; FAO, fatty acid oxidation; Glu, glutamate; GS, glycogen synthesis; KBs, ketone bodies; LC-ACs; long-chain acylcarnitines; LD, lipid droplet; mPTP, mitochondrial permeability transition pore; mtDNA, mitochondrial DNA; OXPHOS, oxidative phosphorylation; Phe, phenylalanine; Poly, polyol pathway; PPP, pentose phosphate pathway; PyrO, pyruvate oxidation; Trp, tryptophan

To investigate representative proteins in each HF phase and understand their biological significance, hierarchical clustering of DEPs was performed alongside gene ontology (GO) and Kyoto Encyclopedia of Genes and Genomes (KEGG) pathway analysis (Fig. 5 and Sup. Figure 2 (Additional file 1)). At 4 months of age, the percentage of DEPs in LV-tissue lysates was rather low (8–9%), which may suggest a relatively preserved cardiac proteome in Tgαq*44 compared to FVB mice (Sup. Table 2 (Additional file 1)). At this stage, moderate alterations in cardiac metabolic protein expression were recognised involving FA transport/oxidation and oxidative phosphorylation downregulation along with glucose utilisation amplification (Fig. 5, Sup. Table 2 (Additional files 1 and 2)). In 8–10-month-old Tgαq*44 mice, modulation of actin cytoskeleton, extracellular matrix (ECM)-receptor interaction, endocytosis, adherens junction, tight junction, protein processing in endoplasmic reticulum, phagosome, and focal adhesion were greatly upregulated, while the downregulated proteins mainly modulated the calcium level/signalling pathway, apoptosis, and metabolic pathways, including valine, leucine, and isoleucine degradation, FA transport/oxidation, citrate cycle (TCA cycle), oxidative phosphorylation, and mitochondrial pyruvate transport/oxidation (Fig. 5, Sup. Tables 3–4 (Additional files 1 and 2)). Notably, myocardial proteins related to glucose uptake and glycolysis were concomitantly augmented in Tgαq*44 mice, although this was not accompanied by increased mitochondrial pyruvate carrier (MPC) expression. Instead, it seems that glucose catabolism was partially routed to the pentose phosphate pathway (PPP) as manifested by the overexpression of PPP proteins. Particularly in 10-month-old Tgαq*44 mice, a sharp increase was observed in protein levels of a number of entities engaged in GLUT4 recycling between intracellular compartments and the plasma membrane (unconventional myosin-Ic (MYO1C) and EH domain-containing protein 2 (EHD2)). In parallel, a pronounced overexpression was noticed for multiple enzymes associated with glucose breakdown into pyruvate (hexokinase 1 and 2 (HK1/HK2), glucose-6-phosphate isomerase (GPI), phosphoglycerate mutase 1 (PGAM1), α-enolase (ENOA), and glycerol kinase (GK)). Intriguingly, the expression of pyruvate dehydrogenase complex (PDHc) seemed to be preserved despite the downregulation of MPC2; however, whether this ultimately results in defective mitochondrial flux through PDH warrants further functional measurements. In 12–14-month-old Tgαq*44 mice, up-regulation was observed in proteins mainly involved in modulating cardiac muscle contraction, protein processing in endoplasmic reticulum, ECM-receptor interaction, and adrenergic signalling in cardiomyocytes, or in proteins related to glycolysis and rerouting of pyruvate towards PPP or lactate (Fig. 5, Sup. Tables 5–6 (Additional files 1 and 2)). In contrast, downregulated proteins were closely associated with ketone body (KB) and branched-chain amino acid (BCAA) catabolism, mitochondrial oxidative metabolism, as well as D-glutamate and aromatic acid degradation. Specifically, in 12-month-old Tgαq*44 mice, the D-beta-hydroxybutyrate dehydrogenase (BDH1) and acetyl-CoA acetyltransferase (ACAT1) protein content was decreased by approximately 49% and 24%, respectively compared to FVB mice. This suppression in the KB catabolic process was further augmented at 14 months, resulting in 66% and 41% downregulation of BDH1 and ACAT1 individually. In line with these findings, the transamination of BCAAs was largely reduced in Tgαq*44 mice starting from 12 months of age (by roughly 52%), as was the expression of many other proteins associated with branched-chain alpha-ketoacid (BCKA) degradation, although the under-expression of the latter was already clearly visible at 8–10 months. Finally, it appears that mitochondrial oxidative metabolism could be greatly impaired in advanced HF, as evidenced by the suppression of pathways linked with FA catabolism, canonical TCA cycle, and oxidative phosphorylation. Nonetheless, further functional assays using metabolic flux or respirometry technology would be required to unequivocally confirm this hypothesis. Importantly, structural and metabolic cardiomyocyte remodelling was related to changes in proteins involved in glycogen and lipid droplet (LD) formation. Specifically, the greatest upregulation of enzymes regulating glycogenesis was observed in Tgαq*44 mice at the age of 12 months, and moderate attenuation was noted at 14 months. An opposite trend was found for LDs, where perilipin-3, an LD-coating protein known to regulate lipid storage and utilisation, was diminished in Tgαq*44 mice at 12 months (Fig. 5 and Additional file 2).

### Longitudinal trajectories of cardiac proteome during HF progression

**Fig. 6:**
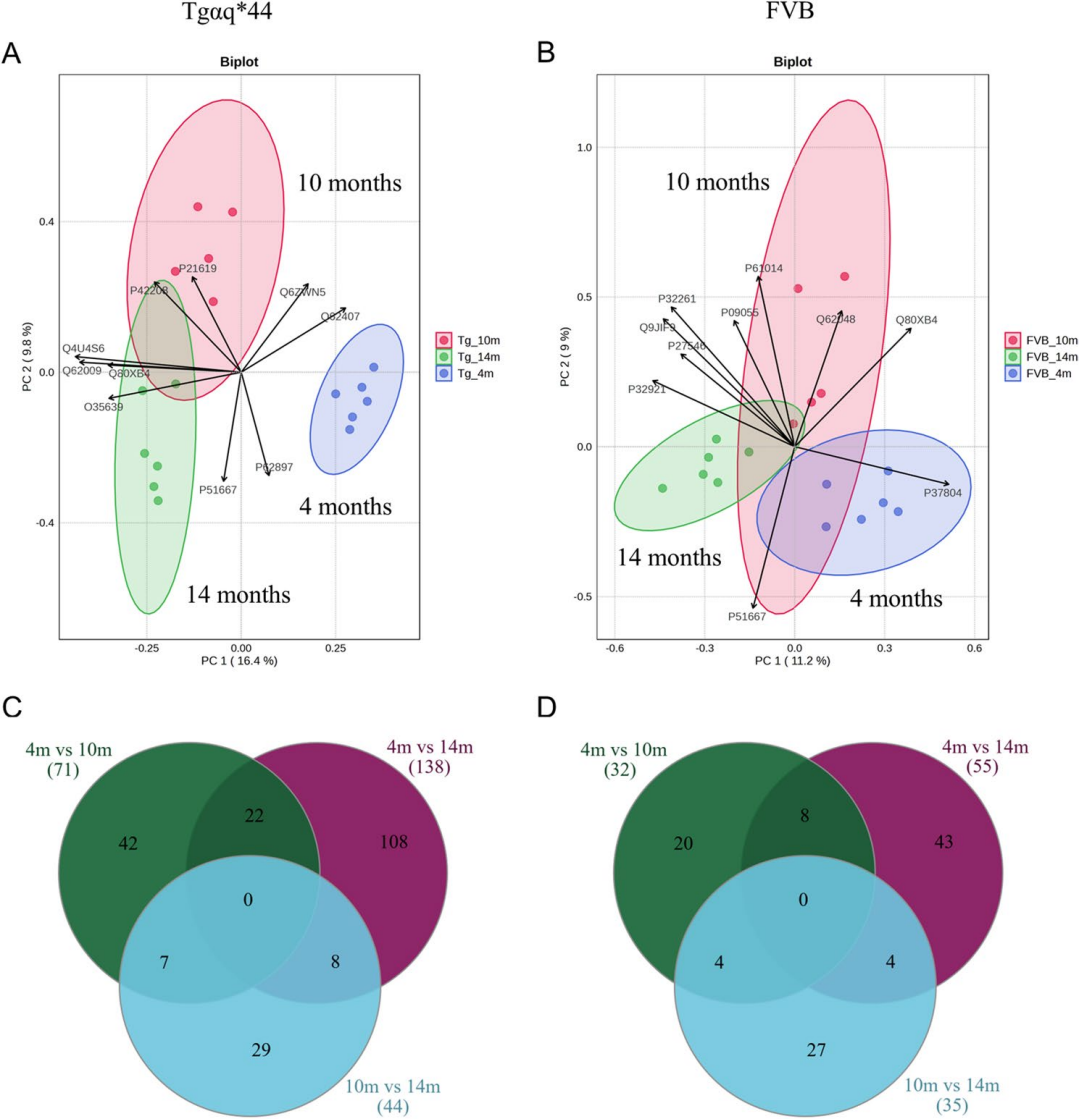
Temporal changes in myocardial protein expression attained across the 4-, 10-, and 14-month time points. PCA biplots showing both PC scores of samples (dots) and loadings of variables (vectors) that were computed for Tgαq*44 (**A**) and FVB (**B**) samples (*n* = 5‒6). Arrows indicate top 10 variables, with length and direction representing loadings. Comparative Venn diagrams to visualise the differences and similarities in the protein profile at different time points in Tgαq*44 (**C**) and FVB (**D**) mice (only statistically meaningful proteins were included)

To track the global proteomic trajectories during HF progression, we analysed data from Tgαq*44 mice with myocardial proteome measurements across three time points (4, 10, and 14 months of age). Principal Component Analysis (PCA) was performed to explore patterns in protein expression profiles across different stages of HF. While the PCA biplot showed sharply defined three clusters (Fig. 6A), 10-month-old Tgαq*44 samples tended to form a partially overlapping but distinguishable group relative to the 14-month-old samples, which were more tightly clustered and positioned away from the 4-month-old samples. In turn, for FVB samples less discernible spatial organisation was found (Fig. 6B). Using the nonparametric Kruskal–Wallis with post-hoc Dunn’s test to identify significant differences in protein abundances, we then recognised 138 and 44 of 1174 proteins that showed statistically significant segregation with end-stage HF phenotype, when compared against early and transition phase, respectively (Fig. 6C, Additional file 3). At the same time, 71 proteins showed different abundance among 10-month-old Tgαq*44 compared to 4-month-old Tgαq*44 samples. Noteworthy, proteomic profiling attained on FVB samples revealed that much smaller fraction of the proteins exhibited significantly differential abundances during the ageing process of the heart (Fig. 6D, Additional file 3).

To further investigate the affected metabolic pathways during HF progression in this time series analysis, we performed gene ontology enrichment analysis, and found that the DEPs were localised mainly in the cellular components of mitochondria (Additional file 3). Consistent with the above, the DEPs were predominantly involved in molecular functions, such as oxidoreductase activity, and biological processes, such as redox homeostasis, FA/BCAA/KB oxidation, oxidative phosphorylation, and mitochondrion organisation. In addition, KEGG pathway enrichment revealed that central carbon metabolism was significantly altered in the LV of Tgαq*44 mice. Precisely, pathways of glycolysis, BCAA catabolism, FA oxidation and oxidative phosphorylation were moderately influenced in the LV from month 4 to month 10, whereas the last time point was dominated by changes in glycolysis, ketolysis, pentose phosphate pathway, lactate biosynthesis and pathways related with mitochondrial oxidative metabolism. Importantly, MPC2 but not MPC1 protein expression was substantially reduced in both 10- and 14-month-old Tgαq*44 mice compared to younger animals. In turn, MPC1 levels increased at 10 months to be further downregulated at 14 months suggesting the ineffective compensation for impaired pyruvate entry into mitochondria. In line with the above, upon progression of HF uncoupling of glycolysis from glucose oxidation might be anticipated that was also manifested by significant suppression of the expression of pyruvate dehydrogenase E1 component subunit beta (PDHB medians: 10.15 vs. 7.53 vs. 7.98 for 4-, 10- and 14-months, respectively) (Additional file 3), despite evident PDK4 (pyruvate dehydrogenase kinase 4) downregulation in 14-month-old animals (Sup. Figure 3 (Additional file 1)).

Importantly, ageing in FVB mice was not associated with the impairment in MPC2 pattern which seemed to be largely preserved (similar to MPC1) (Additional file 3). Instead, improved coupling of glycolysis and glucose oxidation (relative to Tgαq*44 mice) might be expected being further supported by consistent upregulation of PDHA1 (pyruvate dehydrogenase E1 subunit alpha 1) expression and suppression of LDHA (L-lactate dehydrogenase A chain). Nonetheless, it is worth mentioning that the overall glucose oxidation rate could be partially compromised alongside heart ageing given PDK4 overexpression in older (10- and 14-month-old) FVB mice. Adding to the above, in contrast to Tgαq*44 mice, it seems that FA oxidation dominated as the major source of ATP production. Also, it is anticipated that ketone catabolism might be essential for preserving cardiac function during ageing given that aged FVB hearts substantially upregulated ketolytic enzymes (Additional file 3).

Collectively, analysis of temporal progression of metabolic changes during HF development revealed that increased glycolysis not matched by an increase in pyruvate oxidation, due, in part, to MPC2 downregulation, was the earliest substantial metabolic change in HF potentially as the response to a moderate decrease in mitochondrial FA oxidation, occurring before changes in auxiliary fuel (BCAA, KB) oxidation. The progressive reduction in mitochondrial oxidative metabolism and ATP production likely enhanced the compensatory response further stimulating glycolysis and ultimately leading to an increase in lactate and metabolic by-products of PPP secretion as well as glycogen accumulation.

### Loss of metabolic flexibility in the failing heart and greater reliance on glycolysis

To test whether alterations in the expression pattern of metabolic enzymes would translate into shifts in substrate utilisation, energy metabolic rates were measured in 4- and 14-month-old Tgαq*44 and FVB mice in ex vivo isolated hearts perfused according to Langendorff. To profile intermediary metabolites in the coronary effluents, an innovative SPME technology was applied, which both improved enrichment and quantification of analytes in highly diluted samples, and minimised the effects of the challenging matrix on measurement accuracy. As it was proven in numerous previous studies [50–54] and shown here, in contrast to typically used solvent-based sample preparation techniques, SPME provided superior sample clean-up, and favourably affected the sensitivity and accuracy of the applied method. Precisely, SPME enabled the isolation and pre-concentration of metabolites present at even trace levels in complex matrices, while its sample clean-up capabilities prevented cargo (i.e., high salt concentration, macromolecules, cellular debris) from influencing the extraction performance. Collectively, several drawbacks found in conventional extraction techniques might be omitted with SPME ultimately guaranteeing a rapid, cost-effective and robust approach for sample pre-treatment.

**Fig. 7:**
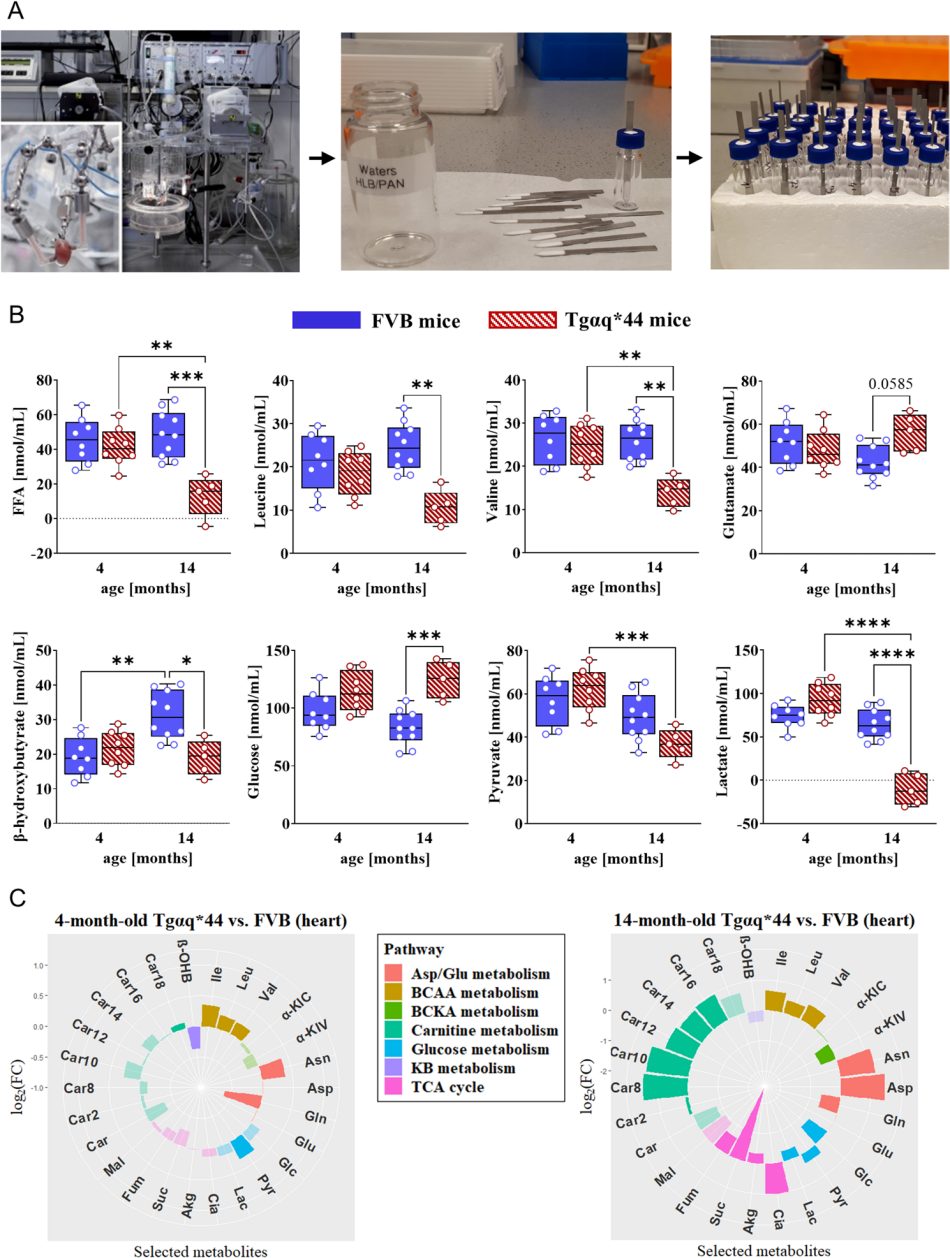
Loss of metabolic flexibility in the failing heart. (**A**) A schematic drawing of the SPME/metabolomics-based experimental design using coronary effluents collected during Langendorff retrograde perfusion of the isolated mouse heart. The Langendorff apparatus is indicated on the left, while SPME microprobes and microextraction are shown in the centre and right of the drawing. (**B**) Myocardial extraction of FFAs, leucine, valine, glutamate, β-hydroxybutyrate and carbohydrates in 4- and 14-month-old FVB and Tgαq*44 mice (*n* = 5‒10/group) measured as transcardiac arteriovenous gradients of relevant metabolites. The changes in the concentration of energy substrates in coronary effluents vs. K-H buffer reflect the cardiac rate of their uptake/utilisation. Coronary effluents were collected at baseline (during the 15-minute stabilisation period before brief ischaemia). Results are expressed as median and range. Statistical significance was determined by a 2-way ANOVA with a Tukey post hoc test; * *p* < 0.05, ** *p* < 0.01, *** *p* < 0.001, **** *p* < 0.0001. (**C**) Polar (flower) plots of myocardial energy metabolites and their downstream catabolites in 4 investigated groups: 4- (*n* = 8) and 14-month-old (*n* = 5‒10) FVB and Tgαq*44 mice. The fold changes (Tgαq*44/FVB) for each metabolite are shown by their distance from the polar plot origin. Statistical differences between each set of group comparisons are denoted by the intensity of the colour (dark colour – the change is statistically meaningful, shaded – non-significant). Data were analysed by unpaired two-tailed Student’s t-test. Polar plots were generated using R (ver. 4.3.1) package ggplot2 (ver. 3.4.3). *Abbreviations*: Akg, α-ketoglutarate; Asn, asparagine; Asp, aspartate; BCAA(s), branched-chain amino acid(s); BCKA(s), branched-chain keto acid(s); Car, carnitine; Car2, acetylcarnitine; Car8, octanoylcarnitine; Car10, decanoylcarnitine; Car12, laurylcarnitine; Car14, myristoylcarnitine; Car16, palmitoylcarnitine; Car18, stearoylcarnitine; Cia, citrate; Fum, fumarate; Glc, glucose; Gln, glutamine; Glu, glutamate; Ile, isoleucine; KB, ketone body; α-KIC, keto(iso)leucine; α-KIV, ketovaline; Lac, lactate; Leu, leucine; Mal, malate; β-OHB, β-hydroxybutyrate; Pyr, pyruvate; Suc, succinate; Val, valine

Cardiac uptake of FAs was fully preserved in 4-month-old Tgαq*44 mice, but significantly reduced in 14-month-old Tgαq*44 as compared to age-matched FVB mice. Transmyocardial arteriovenous differences for the main metabolic substrates in the control and disease groups at rest are summarised in Fig. 7A‒B. Similarly, BCAA (leucine, valine) and KB (β-OHB) extraction was fully maintained in the isolated hearts taken from 4-month-old Tgαq*44 mice but was diminished in 14-month-old Tgαq*44 mice relative to age-matched control animals. There was no significant difference in glutamate uptake in 4-month-old Tgαq*44 but a clear trend towards increased cardiac uptake in 14-month-old Tgαq*44 mice was observed. Of note, myocardial β-OHB uptake seemed to be only slightly reduced when old Tgαq*44 mice were compared against young counterparts. Glucose extraction tended to increase in 4-month-old Tgαq*44 and there was a statistically significant increase in the uptake of glucose in 14-month-old Tgαq*44 mice. Pyruvate and lactate extraction also had a tendency to increase in 4-month-old Tgαq*44 mice but there was a clear-cut decrease in pyruvate and lactate extraction in 14-month-old Tgαq*44 mice. Intriguingly, under Dbn stress, an increase in total substrate consumption was particularly observed in Tgαq*44 mice being largely driven by the preferential uptake of selected energy fuels. Precisely, significantly greater glutamate and β-OHB uptake (~ 30%) was specifically noted in 4-month-old Tgαq*44 mice in stressed vs. rested state. The enhanced uptake of these alternative fuels in the diseased heart might be suggestive of a metabolic adaptation of the myocardium to acute stress. The presented data may also imply that the myocardial capacity to utilise glutamate/β-OHB was increased prior to the development of advanced HF (Sup. Figure 4 (Additional file 1)). In turn, for other metabolic substrates, the difference between the arteriovenous values in the rest and stressed state was not statistically significant, except for lactate, the release of which appeared to increase further (in 14-month-old Tgαq*44 mice).

Consistent with these findings, analysis of metabolite levels in LV tissue samples by LC-MS demonstrated that the failing heart exhibited the concentration profile of energy substrates and metabolic intermediates convergent with an increased reliance on glucose for non-oxidative energy production, lower mitochondrial substrate utilisation, and excessive accumulation of metabolic by-products of incomplete fat or BCAA oxidation (Fig. 7C and Sup. Figure 5 (Additional file 1)). Tissue levels of pyruvate, glycine, and serine were increased in Tgαq*44 hearts, consistent with restricted mitochondrial pyruvate entry and increased flux via the serine biosynthetic pathway (SBP). Notably, the lactate: pyruvate ratio, which reflects the ratio of NADH: NAD^+^ in the cytosol, was unchanged in 4-month-old Tgαq*44 but significantly reduced in 14-month-old Tgαq*44 mice, suggesting that the cytoplasmic redox state became more oxidised in older Tgαq*44 animals. In addition, chronic accumulation of BCAAs, but not BCKAs, was found in Tgαq*44 hearts, accompanied by altered acylcarnitine (AC) metabolism and inflexible mitochondrial fuel utilisation. This clearly suggests that the first step in the catabolism of BCAAs, catalysed by the mitochondrial branched-chain aminotransferase 2 (BCAT2), was affected that may be linked, at least in part, to its diminished expression. Of note, myocardial β-OHB levels were depleted in diseased hearts, which in 14-month-old mice may be, at least partially, linked to defective KB transport (across the plasma membrane) due to reduced monocarboxylate transporter 1 (MCT1) expression. Altogether, an LC-MS–based study on LV tissue samples enabled a comprehensive insight and better mechanistic understanding of the metabolic alterations associated with chronic HF ultimately complementing previous observations found in coronary effluents pointing out profoundly impaired FA, BCAA, and KB oxidation and greater production of non-oxidised glycolytic products in advanced HF.

### Circulating energy-providing substrates and their metabolites are reflective of cardiac dysfunction

**Fig. 8:**
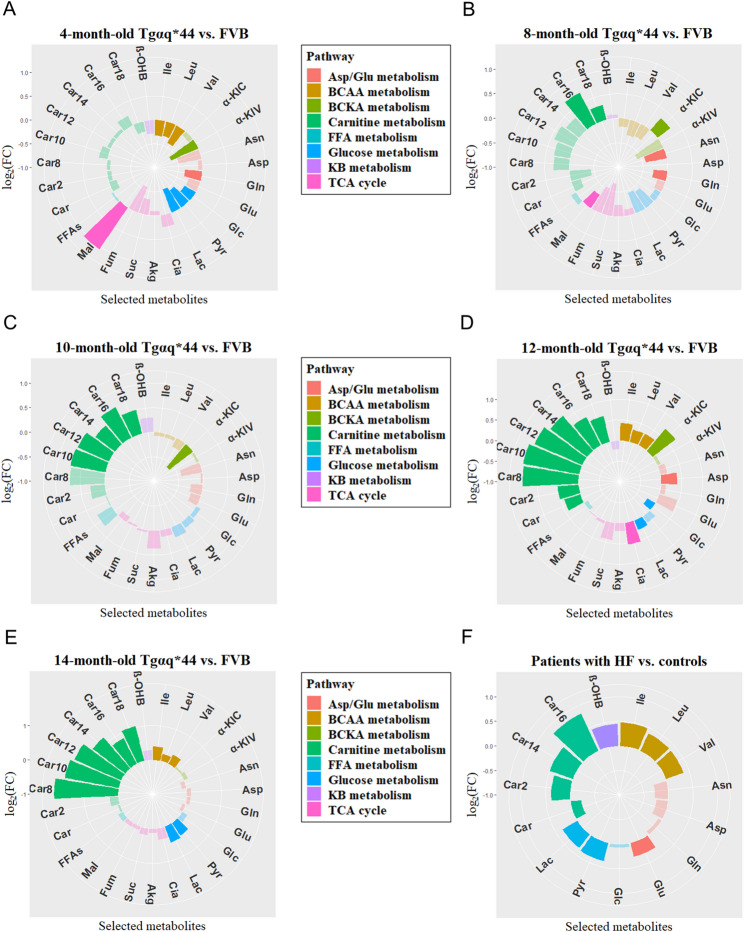
Metabolomic profiling identified circulating biomarkers of cardiac dysfunction. Circulating BCAA, KB, carbohydrate, TCA cycle intermediate and acylcarnitine profile in (**A**‒**E**) murine and (**F**) human heart failure (HF). Polar (flower) plots of plasma energy metabolites and their downstream catabolites in 10 investigated animal groups: 4-, 8-, 10-, 12- and 14-month-old FVB and Tgαq*44 mice (*n* = 7‒10/group) as well as in HF patients and control subjects (*n* = 18‒20/group). The fold changes (HF/Ctrl) for each metabolite are shown by their distance from the polar plot origin. Statistical differences between each set of group comparisons are denoted by the intensity of the colour (dark colour – the change is statistically meaningful, shaded – non-significant). Data were analysed by unpaired two-tailed Student’s t-test. Polar plots were generated using R (ver. 4.3.1) package ggplot2 (ver. 3.4.3). *Abbreviations*: Akg, α-ketoglutarate; Asn, asparagine; Asp, aspartate; BCAA(s), branched-chain amino acid(s); BCKA(s), branched-chain keto acid(s); Car, carnitine; Car2, acetylcarnitine; Car8, octanoylcarnitine; Car10, decanoylcarnitine; Car12, laurylcarnitine; Car14, myristoylcarnitine; Car16, palmitoylcarnitine; Car18, stearoylcarnitine; Cia, citrate; FFA(s), free fatty acid(s); Fum, fumarate; Glc, glucose; Gln, glutamine; Glu, glutamate; Ile, isoleucine; KB, ketone body; α-KIC, keto(iso)leucine; α-KIV, ketovaline; Lac, lactate; Leu, leucine; Mal, malate; β-OHB, β-hydroxybutyrate; Pyr, pyruvate; Suc, succinate; Val, valine

To verify whether altered plasma metabolites can serve as a fingerprint of impaired cardiac energy metabolism along the progression of chronic HF, targeted metabolomic profiling was attained on collected animal and human plasma samples. Plasma levels of BCAAs were elevated in 12- and 14-month-old Tgαq*44 mice compared with age-matched control mice, whereas the BCKA pattern was only slightly affected (Fig. 8A–E). β-OHB increased prior to the development of advanced HF, which may suggest enhanced myocardial capacity to utilise the salutatory fuel. Along with significant disruption of the β-OHB catabolic pathway at 12 months of age, circulating levels of β-OHB were reduced, potentially leading to more severe cardiac dysfunction. A renewed increase in β-OHB observed in plasma at 14 months was not associated with its accelerated extraction/utilisation, reflecting tightly regulated entry into the cardiomyocytes. A similar pattern was documented for FAs, with higher circulating ACs starting at 8 months of age, irrespective of chain length, deliberating defective mitochondrial β-oxidation in Tgαq*44 mice. Increased blood lactate was also prevalent in advanced HF, particularly at 14 months, reflecting accelerated rerouting of pyruvate toward lactate dehydrogenase (LDH)-related conversion. Notably, Tgαq*44 mice displayed marginal changes in plasma levels of TCA-related metabolites or indexes of mitochondrial or cytosolic redox status (Fig. 8A–E and Sup. Figure 6 (Additional file 1)).

Together, through integration of proteomic and metabolomic data as well as those from bioenergetic and functional assays, we tracked cardiac energy metabolism remodelling during development of advanced HF and recognised major affected pathways, including mitochondrial pyruvate import/metabolism, ketolysis, BCAA degradation, carnitine shuttle, and mitochondrial but not peroxisomal FA oxidation. In addition, it seems that alterations in pyruvate utilisation occurred before changes in BCAA and KB oxidation suggesting it may be mechanistically important in the disease progression.

To further examine whether similar systemic perturbations in levels of energy-related metabolites would be found in HF patients, the cohort of HF cases with reduced ejection fraction was investigated and compared against 18 control subjects (Table 1; Fig. 8F). Compared with non-failing controls, HF patients exhibited moderately depressed left ventricular ejection fraction (35.4 ± 9.2%) and ≥ 2 comorbidities as well as demonstrated marked perturbations in routinely measured plasma biochemical and hormonal parameters, including increased NT-pro-BNP (22-fold), triglycerides (1.31-fold), and C-reactive protein (1.93-fold), along with decreased eGFR (0.73-fold). Plasma concentrations of BCAAs, β-OHB, C14:0-AC, and C16:0-AC, and Glu/Gln ratio clearly increased in HF patients in comparison to controls. Furthermore, the by-products of accelerated glycolytic flux (lactate and pyruvate) chronically accumulated in HF blood. Finally, a strong relationship between the severity of dysfunction on the left side of the heart (i.e., LVEF) and the build-up of selected metabolites (i.e., BCAAs, pyruvate, lactate, β-OHB, and palmitoyl-carnitine) was found (Sup. Figure 7 (Additional file 1)).

**Table 1:** Clinical characteristics of study participants

Variables	Control subjects (n = 18)	HF cases (n = 20)	P value
Demographics/anthropometrics:			
Age [y]	57.6±11.2	67.2±11.5	NS
Female sex [%] (n)	44.4 (8)	35 (7)	NS
BMI [kg/m^2^]	27.3±4.8	28.7±5.2	NS
Past medical history, [%] (n):			
Arterial hypertension	44.4 (8)	75 (15)	< .05
Diabetes mellitus	5.56 (1)	35 (7)	< .05
Smoking	16.7 (3)	20 (4)	NS
Obesity	22.2 (4)	35 (7)	NS
Dyslipidaemia	44.4 (8)	85 (17)	< .05
Coronary artery disease	11.1 (2)	60 (12)	< .05
Atrial fibrillation	5.56 (1)	40 (8)	< .05
Chronic kidney disease	11.1 (2)	30 (6)	< .05
Echocardiography:			
LVEF [%]	61.3±5.4	35.4±9.2	< .05
E/E' ratio	6.52 (5.48/7.89)	11.90 (8.14/17.13)	< .05
Laboratory data:			
Fasting blood glucose [mg/dL]	89.0 (84.0/96.0)	102.0 (94.0/112.1)	NS
Total Chol [mg/dL]	210.0 (184.2/240.0)	183.0 (154.0/221.0)	NS
LDL-Chol [mg/dL]	135.8±37.6	115.2±42.2	< .05
HDL-Chol [mg/dL]	57.1±16.1	45.4±13.0	NS
Triglycerides [mg/dL]	98.0 (71.0/136.6)	128.0 (93.0/195.3)	< .05
NT-pro-BNP [pg/mL]	55.0 (35.0/80.0)	1224.5 (526.4/2626.6)	< .05
CRP [mg/L]	1.40 (0.75/2.30)	2.70 (1.34/5.30)	< .05
eGFR [mL/min/1.73 m^2^]	89.15±13.75	65.61±24.21	< .05
Medications (ATC code), [%] (n):			
Antidiabetic agents (A10)	5.56 (1)	25 (5)	< .05
Antithrombotics (B01A)	22.2 (4)	85 (17)	< .05
Diuretics (C03)	5.56 (1)	85 (17)	< .05
Beta-blockers (C07)	16.7 (3)	85 (17)	< .05
Calcium channel blockers (C08)	5.56 (1)	10 (2)	< .05
Agents acting on RAAS (C09)	33.3 (6)	85 (17)	< .05
Lipid-modifying agents (C10)	16.7 (3)	60 (12)	< .05

Note: Data are presented as mean ± SD, median (Q1/Q3), or as relative and absolute frequencies (categorical data). Abbreviations: ATC, anatomical therapeutic chemical classification system; BMI, Body Mass Index; CRP, C-reactive protein; eGFR, estimated glomerular filtration rate; LVEF, left ventricular ejection fraction; NT-pro-BNP, N-terminal pro-brain natriuretic peptide; Q, quartile; RAAS, renin-angiotensin-aldosterone system

## Discussion

**Fig. 9:**
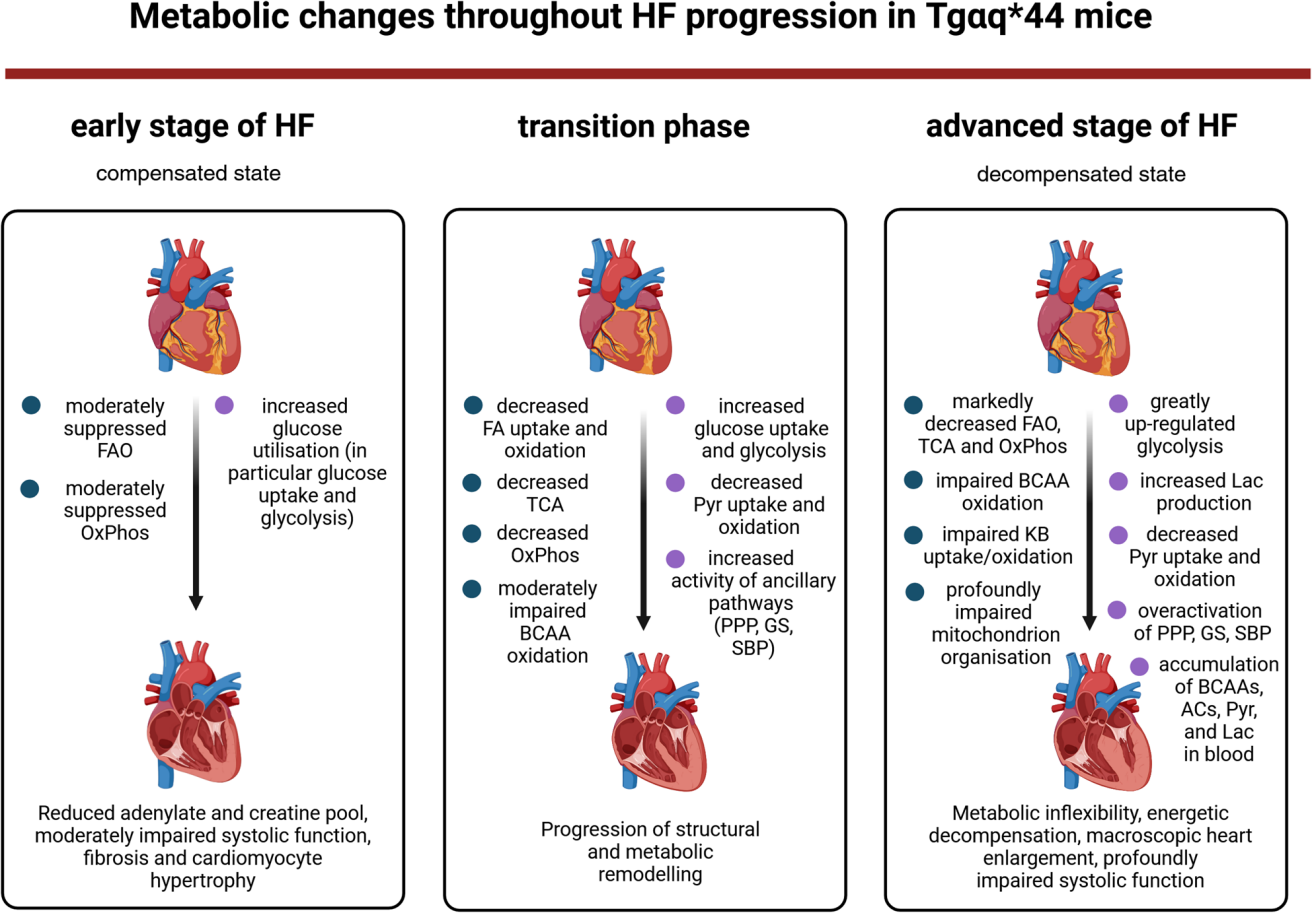
Temporal progression of metabolic changes during HF development in Tgαq*44 mice. Three disease stages (early (compensated), transition, and advanced) show distinct metabolic features characterised by changes in fatty acid and glucose oxidation as well as auxiliary fuel utilisation. ACs, acylcarnitines; BCAA(s), branched-chain amino acid(s); FAO, fatty acid oxidation; GS, glycogen synthesis; KB, ketone body; Lac, lactate; OxPhos, oxidative phosphorylation; PPP, pentose phosphate pathway; Pyr, pyruvate; SBP, serine biosynthetic pathway; TCA, tricarboxylic acid (cycle). The figure was created with a licensed version of BioRender.com

Alterations in cardiac substrate metabolism, in particular of glucose and FAs, are well recognised as important HF mechanisms [3]. However, the role of alternative fuels in modulating this metabolism and its dynamics in the diseased heart have rarely been studied [55–57]. In the present study, we demonstrated that defective catabolism of auxiliary energy fuels, namely BCAAs and KBs, was a prominent feature of metabolic remodelling along the progression of chronic HF which was associated with pyruvate metabolism dysfunction. Specifically, chronic accumulation of BCAAs and impaired KB utilisation were clearly identified in advanced HF, which was accompanied by reduced mitochondrial pyruvate uptake evident as diminished MPC expression. The metabolic rewiring of cardiomyocytes in HF, which involves a decrease in mitochondrial pyruvate oxidation, has been reported in numerous studies [2, 3, 8]. However, molecular mechanisms behind inefficient glucose utilisation due to rerouting of pyruvate to anabolic growth pathways have not been explored previously longitudinally in relation to cardiac function and bioenergetic status of the heart as in the present work. Herein, taking advantage of the unique Tgαq*44 mice characterised by a prolonged course of HF progression [20, 21, 27, 29], and by integrating proteomic, metabolomic, and functional data, we delineated alterations in myocardial protein expression patterns during the progression of HF in relation to energy fuel utilisation and intermediary metabolism deregulation. Figure 9 illustrates the metabolic signatures at various disease stages. This provided new insights into the pathophysiology of HF, which may serve as the basis for future mechanistic research and deliver conceptually new molecular markers to target.

In HF in Tgαq*44 mice, the observed energy metabolic switch was associated with reductions in FA oxidation and a concurrent increase in glucose utilisation, which could be interpreted as reactivation of the foetal gene expression, as described elsewhere [8, 58]. The increase in glucose utilisation was linked with overexpression of proteins related to the recycling/internalisation of glucose transporter 4 (GLUT4) and the upregulation of glycolysis-regulating proteins. Since ATP generation from glucose oxidation can improve oxygen efficiency compared to FA oxidation [3], this metabolic remodelling during the initial stages of HF may be an adaptive response to cope with the extra workload. Conversely, the amount of ATP produced by upregulating glycolytic flux is not sufficient to meet the increase in metabolic demand and heart muscle wall stress. Accordingly, if reduced FA oxidation cannot be fully compensated by increased glucose oxidation, energy starvation and accelerated decline of cardiac function may result. Thus, reverting to a foetal pattern seems to be critical for a short-term adaptation , but it appears to be maladaptive in the long run. In our study, overt HF in Tgαq*44 mice but not early phase of HF was associated with altered substrate utilisation and defects in FA, pyruvate, BCAA, and KB oxidation, alongside prominent elevation of lactate, clearly suggesting a transition from adaptive to maladaptive phase of cardiac metabolism remodelling. Even a moderate compensatory increase in mitochondrial pyruvate transport/oxidation, and likely FA oxidation next to elevated GTP level, we found in 12-month-old Tgαq*44 mice, appeared insufficient to substantially boost impaired energy metabolism. Our findings are consistent with previous results indicating that metabolic processes, including mitochondrial-related pathways, are decreased in severe HF [2, 3, 8, 55]. They are, however, in partial disagreement with the notion of metabolic inflexibility in the failing heart. Intriguingly, Watson et al. demonstrated that in compensated non-ischemic cardiomyopathy with severe LV systolic dysfunction, the heart retained substantial metabolic substrate flexibility, and suggested that promoting FAO might be a beneficial therapeutic intervention challenging the existing doctrine in HF metabolism [59].

Mitochondrial pyruvate uptake/utilisation was clearly impaired in the failing heart in Tgαq*44 mice, as evidenced by downregulation of MPC1/2 and progressive accumulation of myocardial pyruvate, specifically at the stage of advanced HF in this model. In addition, there was increased partitioning of glycolysis-derived metabolites into other cytosolic pathways, such as the PPP and the SBP, along with glycogen synthesis, which, in concert with changes in mitochondrial substrate utilisation, apparently contributed to cardiac dysfunction. Shunting of glucose into alternative pathways and, potentially, tissue acidosis secondary to increased lactate, raises the possibility of cardiotoxicity-related mechanisms being involved in adverse structural and functional remodelling. Our observations are in line with several studies reporting that MPC-mediated mitochondrial pyruvate utilisation is essential for myocardial metabolism and function, and adaptations under stressed conditions [60–63]. Indeed, Fernandez-Caggiano et al. [61] and McCommis et al. [62] demonstrated that MPC is deactivated in failing human and mouse hearts. The loss of MPC in the murine heart resulted in age-related dilated cardiomyopathy, which was associated with redirection of glycolytic intermediates into the pentose phosphate and serine biosynthetic pathways, correlating with adverse LV remodelling [60, 61]. Moreover, it was proven that LV remodelling was reversed or attenuated by increasing the availability of alternative substrates through a ketogenic or high-fat diet or, alternatively, by accelerating pyruvate mobilisation into the TCA by inducing the expression of MPC in an HF model [60, 62].

In addition, it seems that the pyruvate-lactate axis serves as a critical regulatory hub in the metabolism of the failing heart [64]. This node is maintained by a precise regulation of the disposition of pyruvate, including mitochondrial uptake and cellular export as lactate. We identified downregulation of both MPC isoforms and MCT1 in 14-month-old Tgαq*44 mice, implying that this balance was clearly disrupted in advanced HF. Regaining this homeostasis may potentially ameliorate the HF phenotype. Intriguingly, MCT1 and MPC1 were increased in 12-month-old compared to 8–10-month-old Tgαq*44 mice, but, in the context of an acute haemodynamic load, this short-term compensation mechanism was insufficient to rescue Tgαq*44 hearts from decompensation.

Chronic accumulation of BCAAs, but not BCKAs, was consistently found in Tgαq*44 hearts and plasma. Unlike most amino acids, which are mainly degraded in the liver, BCAAs are extensively oxidised in extrahepatic tissue, although the significance of such a metabolic pattern has not been completely clarified [65]. Emerging evidence shows that the heart expresses the highest levels of enzymes engaged in the BCAA catabolic pathway, but it appears that BCAA oxidation contributes to a very small fraction of energy pool production in the heart [57]. Instead, BCAAs play a pivotal role in hormonal secretion/action and intracellular signalling [66, 67]. Interestingly, in several investigations, a distinct link was found between defective BCAA catabolism and impaired glucose oxidation [57, 68, 69]. Specifically, Li et al. revealed that chronic accumulation of BCAAs downregulated the hexosamine biosynthetic pathway (HBP) and inactivated pyruvate dehydrogenase complex (PDH) by reducing the post-translational O-GlcNAc-related modification of the E2/3/3 bp subunits [57]. Notably, even though the above metabolic remodelling was well tolerated under unstressed conditions, it rendered the hearts (defective in BCAA catabolism) vulnerable to ischemic injury. However, either improving BCAA catabolism or normalising glucose utilisation by overexpressing GLUT1 rescued the metabolic and functional outcome. Intriguingly, Uddin et al. have recently shown that an accumulation of BCKAs, but not BCAAs, in the heart primarily mediates insulin resistance, and that manipulating BCKAs may represent an attractive therapeutic strategy to improve insulin signalling, energy metabolism, and contractile function in the heart in the settings of diabetes, obesity, and HF [68, 69]. Thus, BCAA catabolism seems to be essential for the homeostasis of glucose metabolism, although BCAA oxidation appears to be dispensable for energy production. Blood β-OHB was generally elevated in Tgαq*44 mice, though the difference in plasma levels of β-OHB in Tgαq*44 compared to FVB mice was modest. These raised circulating levels of β-OHB may at least partly be attributable to increased FFA mobilisation in response to augmented neurohormonal stimulation [70]. Intriguingly, a parallel fall in blood KB and FFA concentrations was observed in Tgαq*44 mice at 12 months, a depletion which may acutely decrease cardiac work and efficiency as reported in previous investigations [71]. KBs are considered a more efficient energy source than glucose or FFAs, providing more energy per metabolised carbon than glucose and requiring less oxygen than FFAs for ATP generation. Therefore, defects in either the synthetic or oxidative arms of KB metabolism may adversely affect cardiovascular energetic machinery [14, 72]. Indeed, these systemic maladaptations paralleled pathologic cardiac remodelling towards decreased expression of enzymes involved in KB oxidation, and subsequent rapid progression to overt HF. Consistent with these results, our previous study, which demonstrated the importance of bidirectional crosstalk between the liver and heart in HF, found increased ketogenesis in the liver of 4- and 12-month-old Tgαq*44 mice exposed to TG (triglyceride)-rich lipoproteins [29].

It is noteworthy that our conclusions seem to be contradictory to prior findings on the adaptive role of ketone metabolism in HF in the restoration of cardiac function and attenuation of disease progression [9, 14, 15]. In particular, enhanced cardiac ketone utilisation during HF has been consistently attributed to increased KB availability due to systemic ketosis and a cardiac autonomous upregulation of ketolytic enzymes. In contrast, our study suggests that myocardial uptake of KB and regulation of ketolytic enzymes may be more critical in determining the full oxidation capacity. Nonetheless, the possibility cannot be ruled out that the degree of cardiac ketone utilisation in HF may be sensitive to disease aetiology. Consistent with the above, Koay et al. demonstrated the mechanistic importance of the myocardial ketogenic pathway in HFpEF [73]. Precisely, they proved the critical role of the canonical ketogenic enzyme, HMGCS2 (3-hydroxy-3-methylglutaryl-coenzyme A synthase 2), in the pathogenesis and management of HFpEF with NAD+ therapy. Importantly, recent studies have provided key evidence for a reciprocal relationship between KB utilisation and glucose and FA uptake [74, 75]. Specifically, KB infusion in healthy human volunteers was associated with increased myocardial blood flow but decreased myocardial glucose extraction due to interrupted GLUT4 translocation [74]. Aligned with these findings, β-OHB appeared to inhibit myocardial FA uptake through a mechanism independent of malonyl-CoA levels [75]. Nevertheless, the consequences of this inhibitory effect of KBs on myocardial glucose and FA uptake require further characterisation.

There is a widely held concept that in HF the heart ‘switches from FA to glucose metabolism’. However, based on our temporal multi-omic analysis we can propose that this concept might be more fittingly re-worded as the failing heart ‘switches from mitochondrial oxidative phosphorylation to glycolysis for ATP production’. In the early stage of HF, cardiac remodelling characterised by moderately increased glycolysis likely due to slightly suppressed FA oxidation played a compensatory, cardioprotective role. However, upon progression of HF, FA oxidation and overall mitochondrial oxidative activity appeared to be substantially decreased that stimulated the robust compensatory response resulting in increased glucose uptake and glycolytic rates, but this upregulation seemed to be not sufficient to compensate for a drop in ATP production. While glycolysis was up-regulated in the failing heart, the subsequent mitochondrial oxidation of glycolytically derived pyruvate (glucose oxidation) seemed to be acutely decreased that was driven (at least in part) by MPC deficiency. Along with increased rate of cardiac glycolysis and substantial downregulation of MPC2 expression, LV function started to deteriorate suggesting that maintaining myocardial glucose oxidation might be central to cardiac energetics and overall heart function. BCAA and ketone oxidation also diminished with the progression of cardiac dysfunction, despite having a minor contribution to cardiac ATP production. Nonetheless, it is anticipated that specifically BCAAs and BCKAs may be important signalling molecules which accumulation may adversely affect cardiac structure and energy metabolism in HF. Overall, higher flux through glycolysis in HF may initially represent a compensatory mechanism to maintain ATP production; however over time the mismatch between glycolysis and pyruvate utilisation with parallel suppression of mitochondrial oxidative metabolism becomes maladaptive aggravating energy deficit that contributes to the severity of contractile dysfunction. Compromised mitochondrial function, due to reduced mitochondrial biogenesis, increased reactive oxygen species (ROS) production or impairments in mitofission/mitophagy as well as alterations in substrate control or transcriptional regulation may potentially contribute to altered energy metabolism in the setting of HF [3, 5]. In addition, diminished abundance of the cardiac isoform of PFK-2 (phosphofructo-2-kinase), a critical glycolytic regulator, may possibly translate into ancillary pathway activation [76]. However, precise molecular mechanisms warrant further detailed investigation.

While our study provides valuable insights into cardiac intermediary metabolism deregulation in HF, we should acknowledge several limitations. One is that postulated impaired mitochondrial import of pyruvate as evidenced by reduced MPC expression and rerouting of carbon to the anabolic pathways, and causal relationship with HF should be corroborated by 13 C labelled glucose tracing and cardiac-specific deletion of MPC (particularly MPC2) to unambiguously confirm that loss of MPC in the heart causes glycogen and glycolytic metabolite accumulation, and ultimately HF. Similarly, also potentially compromised mitochondrial oxidative metabolism should be verified by functional assays, such as an isotope-based metabolic flux analysis or by measuring OCR (Oxygen Consumption Rate) changes in isolated mitochondria/cardiac slices. In line with the above, given the impairment of mitochondrial function in HF, proved by reduced ATP production, it would be imperative to display mitochondrial structural damage, impaired mitophagy, and increased ROS production next to differential gene expression related to participating mechanisms. Another limitation is that while the study comprehensively describes metabolic and molecular remodelling, it does not directly connect these alterations to structural changes manifested as fibrosis or hypertrophy in the same animals. The authors are aware that incorporating histological assessments of fibrosis and morphometric measurements of cardiac size paired with metabolic/bioenergetic measurements would substantiate this relationship and causality. Nonetheless, histological evaluation of cardiac changes was presented in numerous previous papers, thus, the authors decided to comment on only most important findings coming from these studies. Notably, changes in protein groups related to structural reorganisation were presented in this study alongside metabolic alterations that delivers some insights into structural remodelling throughout the entire course of the disease. Another factor not accounted for in our studies is association of metabolic facets with inter-connected mechanisms, such as mitochondrial calcium handling, inflammatory pathways, or apoptotic mechanisms. Finally, as in previous works using this model, only female mice were used, thus, we cannot be sure whether the findings of this study are also relevant to male Tgαq*44 mice. The potential influence of sex warrants further exploration.

In conclusion, despite evidence for adaptive mechanisms by which predominantly glycolysis-derived carbons fuel energy metabolism in Tgαq*44 hearts, these hearts inexorably progress to HF. This could be at least partially related to defects in auxiliary fuel oxidation and impaired mitochondrial pyruvate transport. Our results demonstrated that MPC-mediated mitochondrial pyruvate utilisation, along with BCAA and KB oxidation, are essential for the partitioning of glucose-derived cytosolic metabolic intermediates, as well as for adequate ATP supply and balanced cellular signalling. Accordingly, our findings suggest that prevention of glycolytic metabolite accumulation by feeding alternative substrates (e.g., ketones or short-chain FAs) or improving MPC-mediated pyruvate utilisation may rescue failing hearts from adverse structural, metabolic, and functional remodelling at the end-stage of HF.

## Supplementary Information

Below is the link to the electronic supplementary material.

Supplementary Material 1

Supplementary Material 2

Supplementary Material 3

## Data Availability

The data that support the findings of this study are available from the corresponding author upon reasonable request.
