# Supplementary material for: Defects in auxiliary fuel oxidation and mitochondrial pyruvate transport mark transition to overt heart failure in Tgαq*44 mice
Source: J Transl Med. 2026 Mar 3;24:485. doi: 10.1186/s12967-026-07883-y (PMC13063859; doi:10.1186/s12967-026-07883-y)
Supplement: Supplementary file 1 — Supplementary Material 1 [file 12967_2026_7883_MOESM1_ESM.docx]

**SUPPLEMENTARY FILE**

**Defects in auxiliary fuel oxidation and mitochondrial pyruvate transport mark transition to overt heart failure in Tgαq*44 mice**

Mariola Olkowicz^1,2*^, Agata Jedrzejewska^3^, Urszula Tyrankiewicz^1^, Filip Fedak^1,4^, Piotr Berkowicz^1^, Grzegorz Kwiatkowski^1^, Hernando Rosales-Solano^5^, Kanchan Sinha Roy^5^, Agnieszka Karas^1,4^, Oliwia Krol^3^, Marta Tomczyk^3^, Ryszard T. Smolenski^3^, Janusz Pawliszyn^5^, Stefan Chlopicki^1,6^

^1^ Jagiellonian Centre for Experimental Therapeutics (JCET), Jagiellonian University, Bobrzynskiego 14, 30-348 Krakow, Poland;

^2^ Department of Biopharmaceutics and Pharmacodynamics, Medical University of Gdansk, Hallera 107, 80-416 Gdansk, Poland;

^3^ Department of Biochemistry, Medical University of Gdansk, Debinki 1, 80-211 Gdansk, Poland;

^4^ Doctoral School of Exact and Natural Sciences, Jagiellonian University, Lojasiewicza 11, 30-348 Krakow, Poland;

^5^ Department of Chemistry, University of Waterloo, 200 University Avenue West, Waterloo, ON, N2L 3G1, Canada;

^6^ Department of Pharmacology, Jagiellonian University Medical College, Grzegorzecka 16, 31-531 Krakow, Poland

* Corresponding author: *E-mail address*: m.olkowicz@gumed.edu.pl (M. Olkowicz).

**Sup. Table 1.** Selected echocardiographic variables in 4-, 8-, 12- and 14-month-old FVB and Tgαq*44 mice.

|  | FVB 4 m | FVB  8 m | FVB  12 m | FVB  14 m | Tgαq*44  4 m | Tgαq*44  8 m | Tgαq*44  12 m | Tgαq*44  14 m |
| --- | --- | --- | --- | --- | --- | --- | --- | --- |
| Body mass [g]  HR [bpm]  EF [%]  FS [%]  LV mass [g]  LVMI [mg/g]  RWT  IVSd [mm]  IVSs [mm]  LVIDd [mm]  LVIDs [mm]  LVPWd [mm]  LVPWs [mm] | 24.80 ± 1.64  163.30 ± 63.71  71.47 ± 3.06  35.27 ± 2.48  0.11 ± 0.01  4.26 ± 0.45  0.45 ± 0.12  0.88 ± 0.15  1.25 ± 0.21  3.60 ± 0.36  2.33 ± 0.26  0.80 ± 0.16  1.19 ± 0.22 | 30.39 ± 4.43  181.80 ± 69.45  71.98 ± 1.83  35.67 ± 1.44  0.14 ± 0.02  4.56 ± 0.61  0.50 ± 0.07  1.03 ± 0.18  1.50 ± 0.26  3.76 ± 0.23  2.42 ± 0.18  0.94 ± 0.10  1.20 ± 0.14 | 28.50 ± 2.16  185.50 ± 61.94  68.14 ± 5.47  32.92 ± 3.72  0.14 ± 0.02  4.94 ± 0.69  0.49 ± 0.11  1.03 ± 0.15  1.37 ± 0.18  3.80 ± 0.32  2.56 ± 0.30  0.93 ± 0.17  1.22 ± 0.21 | 31.50 ± 2.56  195.00 ±  33.77  68.75 ± 3.20  33.25 ± 2.36  0.14 ± 0.02  4.49 ± 0.78  0.47 ± 0.09  1.03 ± 0.17  1.34 ± 0.17  3.79 ± 0.22  2.53 ± 0.16  0.89 ± 0.16  1.19 ± 0.19 | 24.42 ± 2.33  180.00 ± 45.89  65.94 ± 4.63 *****  31.22 ± 3.44 *****  0.12 ± 0.02 *****  4.97 ± 0.80 *****  0.56 ± 0.04 *  1.02 ± 0.18  1.31 ± 0.26  3.43 ± 0.10  2.36 ± 0.14  0.96 ± 0.07 *****  1.22 ± 0.17 | 29.50 ± 2.50 **$**  166.60 ± 56.34  57.00 ± 3.71 ***$**  25.40 ± 2.22 ***$**  0.14 ± 0.02  4.92 ± 0.84  0.55 ± 0.09  1.20 ± 0.23 *****  1.49 ± 0.31  3.52 ± 0.25 *****  2.63 ± 0.22 *****  0.96 ± 0.11  1.15 ± 0.16 | 29.13 ± 3.67 **$**  188.00 ± 59.07  46.62 ± 4.20 ***$#**  19.70 ± 2.15 ***$#**  0.16 ± 0.04  5.67 ± 1.37  0.49 ± 0.19  0.99 ± 0.18  1.21 ± 0.27  4.03 ± 0.21 ***$#**  3.32 ± 0.18 ***$#**  0.98 ± 0.33  1.03 ± 0.25 | 28.30 ± 1.57 *****  158.60 ± 34.25 *****  50.04 ± 8.22 ***$#**  21.67 ± 4.66 ***$**  0.20 ± 0.04 ***$#**  6.91 ± 1.45 ***$#**  0.62 ± 0.07 *****  1.21 ± 0.22  1.56 ± 0.27  3.87 ± 0.50 **$**  3.03 ± 0.41 ***$#**  1.18 ± 0.10 ***#**  1.20 ± 0.29 |

The data are presented as the mean ± SD; *n* = 5–8 (with 2‒3 technical replicates); ******P* < 0.05 for Tgαq*44 mice vs. age-matched FVB mice (Student’s t test or Mann–Whitney test), **$** *P* < 0.05 for 14- 12- or 8- vs. 4-month-old Tgαq*44 mice, **#** *P* < 0.05 for 14- or 12- vs. 8-month-old Tgαq*44 mice (one-way ANOVA with post hoc Tukey’s test or Kruskal–Wallis test with post hoc Dunn’s test). Legend: *HR*, heart rate; *EF*, ejection fraction; *FS*, fractional shortening; *LV*, left ventricle; *LVMI*, left ventricular mass index; *RWT*, relative wall thickness; *IVSd*, interventricular septal thickness in diastole; *IVSs*, interventricular septal thickness in systole; *LVIDd*, left ventricular internal diameter in diastole; *LVIDs*, left ventricular internal diameter in systole; *LVPWd*, left ventricular posterior wall thickness in diastole; *LVPWs*, left ventricular posterior wall thickness in systole.

**Sup. Table 2.** Differentially expressed proteins (DEPs) in the dataset: **4-month-old Tgαq*44 vs. age-matched control FVB mice** (fold change (Tgαq*44 /Control) ≥1.2 or ≤0.8 and adjusted *p* value <0.05); *n* = 6/group.

| No. | Accession  number | Annotation | FC (Tg/Ctrl) | Molecular Function |
| --- | --- | --- | --- | --- |
| 1  2  3  4  5  6  7  8  9  10  11  12  13  14  15  16  17  18  19  20  21  22  23  24  25  26  27  28  29  30  31  32  33  34  35  36  37  38  39  40  41  42  43  44  45  46  47  48  49  50  51  52  53  54  55  56  57  58  59  60  61  62  63  64  65  66  67  68  69  70  71  72  73  74  75  76  77  78  79  80  81  82  83  84  85  86  87  88  89  90  91  92  93  94  95  96  97  98  99  100  101  102  103  104  105  106  107  108  109  110  111  112  113  114  115  116 | Q9DCN2  Q7TPW1  P20918  Q9JK53  P27546  P27773  P51410  P29341  Q80XB4  P62307  Q60605  Q62048  Q9QZ88  P63325  Q91W90  Q9ESD7  Q8R035  P62082  Q61553  O88746  P32261  Q9EQK5  Q9JK92  O70400  P14869  P11276  Q8BMK4  Q8R2Q4  O88587  Q80X90  Q8BG05  Q61147  O35737  Q04857  P09103  Q9QYB1  P15864  P63001  P35979  P62908  P20152  P26039  P62897  Q91Z83  P08113  Q8BH64  P60710  Q8VDD5  P19324  P48678  P62702  Q68FD5  Q61554  Q01853  Q8VHX6  P60335  Q8BZF8  Q00896  O08638  P17182  Q9EQP2  Q1XH17  P14602  P16045  P62962  P62737  Q9JLV1  P80314  P16546  Q8BKZ9  Q9JKS4  P15508  P34884  P50752  Q9D051  Q8VDM4  P58252  P99024  Q8CGK3  P68134  O35206  Q7TMM9  P50544  Q8K2B3  E9Q401  P52825  Q9JHI5  P62259  Q4VAE3  Q924X2  Q91VM9  P17563  Q2TPA8  Q8CC88  Q9CQ62  Q9DCM0  O35459  Q8VCT4  P30416  Q8K4Z3  Q9EQ20  Q8CHS7  Q99LP6  Q8R164  Q9CQ54  Q9D1I5  P56376  Q9WTP6  Q924D0  Q9WVL0  Q78IK2  P03930  P22315  O88696  Q9CQ92  P62281 | NADH-cytochrome b5 reductase 3  Nexilin  Plasminogen  Prolargin  Microtubule-associated protein 4  Protein disulfide-isomerase A3 (PDIA3)  60S ribosomal protein L9  Polyadenylate-binding protein 1  Nebulin-related-anchoring protein  Small nuclear ribonucleoprotein F  Myosin light polypeptide 6  Astrocytic phosphoprotein PEA-15  Vacuolar protein sorting-associated protein 29  40S ribosomal protein S10  Thioredoxin domain-containing protein 5  Dysferlin  Peptidyl-tRNA hydrolase ICT1, mitochondrial  40S ribosomal protein S7  Fascin  Target of Myb1 membrane trafficking protein  Antithrombin-III  Major vault protein  Heat shock protein beta-8  PDZ and LIM domain protein 1  60S acidic ribosomal protein P0  Fibronectin  Cytoskeleton-associated protein 4  Ribosome-releasing factor 2, mitochondrial  Catechol O-methyltransferase  Filamin-B  Heterogeneous nuclear ribonucleoprotein A3  Ceruloplasmin  Heterogeneous nuclear ribonucleoprotein H  Collagen alpha-1(VI) chain  Protein disulfide-isomerase (PDIA1)  Chloride intracellular channel protein 4  Histone H1.2  Ras-related C3 botulinum toxin substrate 1  60S ribosomal protein L12  40S ribosomal protein S3  Vimentin  Talin-1  Cytochrome c, somatic  Myosin-7  Endoplasmin  EH domain-containing protein 2  Actin, cytoplasmic 1  Myosin-9  Serpin H1  Prelamin-A/C  40S ribosomal protein S4, X isoform  Clathrin heavy chain 1  Fibrillin-1  Transitional endoplasmic reticulum ATPase  Filamin-C  Poly(rC)-binding protein 1  Phosphoglucomutase-like protein 5  Alpha-1-antitrypsin 1-3  Myosin-11  Alpha-enolase  EH domain-containing protein 4  Tripartite motif-containing protein 72  Heat shock protein beta-1  Galectin-1  Profilin-1  Actin, aortic smooth muscle  BAG family molecular chaperone regulator 3  T-complex protein 1 subunit beta  Spectrin alpha chain, non-erythrocytic 1  Pyruvate dehydrogenase protein X component, mitochondrial  LIM domain-binding protein 3  Spectrin beta chain, erythrocytic  Macrophage migration inhibitory factor  Troponin T, cardiac muscle  Pyruvate dehydrogenase E1 component subunit beta, mitochondrial  26S proteasome non-ATPase regulatory subunit 2  Elongation factor 2  Tubulin beta-5 chain  Lon protease homolog, mitochondrial  Actin, alpha skeletal muscle  Collagen alpha-1(XV) chain  Tubulin beta-2A chain  Very long-chain specific acyl-CoA dehydrogenase, mitochondrial  Succinate dehydrogenase [ubiquinone] flavoprotein subunit, mitochondrial  Ryanodine receptor 2  Carnitine O-palmitoyltransferase 2, mitochondrial  Isovaleryl-CoA dehydrogenase, mitochondrial  14-3-3 protein epsilon  Transmembrane protein 65  Carnitine O-palmitoyltransferase 1, muscle isoform  Inorganic pyrophosphatase 2, mitochondrial  Methanethiol oxidase  Hydroxysteroid dehydrogenase-like protein 2  von Willebrand factor A domain-containing protein 8  2,4-dienoyl-CoA reductase [(3E)-enoyl-CoA-producing], mitochondrial  Persulfide dioxygenase ETHE1, mitochondrial  Delta(3,5)-Delta(2,4)-dienoyl-CoA isomerase, mitochondrial  Carboxylesterase 1D  Peptidyl-prolyl cis-trans isomerase FKBP4  NAD(P)H-hydrate epimerase  Methylmalonate-semialdehyde dehydrogenase [acylating], mitochondrial  Dehydrogenase/reductase SDR family member 7C  GrpE protein homolog 1, mitochondrial  Valacyclovir hydrolase  NADH dehydrogenase [ubiquinone] 1 subunit C2  Methylmalonyl-CoA epimerase, mitochondrial  Acylphosphatase-1  Adenylate kinase 2, mitochondrial  Reticulon-4-interacting protein 1, mitochondrial  Maleylacetoacetate isomerase  ATP synthase membrane subunit K, mitochondrial  ATP synthase protein 8  Ferrochelatase, mitochondrial  ATP-dependent Clp protease proteolytic subunit, mitochondrial  Mitochondrial fission 1 protein  40S ribosomal protein S11 | 4.85  4.33  3.83  3.4  3.23  3.01  2.93  2.75  2.75  2.52  2.39  2.28  2.27  2.24  2.22  2.22  2.21  2.19  2.12  2.12  2.12  2.11  2.07  2.03  2.02  2  2  2  1.95  1.9  1.87  1.83  1.83  1.82  1.80  1.78  1.78  1.77  1.69  1.68  1.67  1.67  1.66  1.63  1.62  1.61  1.61  1.60  1.55  1.48  1.47  1.46  1.45  1.44  1.44  1.43  1.42  1.42  1.42  1.41  1.40  1.39  1.39  1.39  1.38  1.35  1.35  1.32  1.32  1.31  1.30  1.3  1.29  1.28  1.27  1.26  1.26  1.26  1.24  1.23  1.23  1.22  0.88  0.84  0.84  0.84  0.83  0.81  0.8  0.79  0.75  0.75  0.74  0.73  0.71  0.69  0.68  0.64  0.64  0.63  0.63  0.63  0.61  0.60  0.59  0.55  0.54  0.54  0.54  0.50  0.49  0.49  0.47  0.47  0.44  0.26 | Cholesterol biosynthetic process  Regulation of cell migration  Blood coagulation/Tissue remodelling  Cellular senescence  Microtubule cytoskeleton organization  Protein folding  Cytoplasmic translation  mRNA processing  Actin cytoskeleton organization  mRNA splicing  Muscle contraction  Apoptotic process  Endocytic recycling/protein transport  Cytoplasmic translation  Protein folding  Skeletal muscle tissue regeneration  Mitochondrial translation  Cytoplasmic translation  Actin cytoskeleton organization  Autophagosome-lysosome fusion  Blood coagulation  Regulation of signal transduction  Cellular response to unfolded proteins  Actin cytoskeleton organization  Cytoplasmic translation  Integrin-mediated signalling  Anchoring of the ER to microtubules  Mitochondrial translation  Catecholamine catabolic process  Actin cytoskeleton organization  mRNA splicing  Iron transport across the cell membrane  mRNA processing  Cell adhesion  Protein folding  Transport chloride ions/ angiogenesis  Chromatin organization  Actin cytoskeleton organization  Cytoplasmic translation  Cytoplasmic translation  Intermediate filament organization  Cell-cell adhesion/integrin activation  Intrinsic apoptotic signalling  Muscle contraction/hypertrophy  Processing/transport of secreted proteins  Internalization of GLUT4  Cardiac muscle contraction  Actin cytoskeleton reorganization  Collagen fibril organization  Nuclear assembly/chromatin organization  Cytoplasmic translation  Early autophagosome formation  Regulation of function of elastic fibers  Autophagosome maturation  Sarcomere organization  mRNA processing  Cell adhesion  Response to cytokines  Muscle contraction  Canonical glycolysis  Membrane reorganization/tubulation  Cell membrane repair  Chaperone-mediated protein folding  Apoptotic process  Actin cytoskeleton organization  Vascular associated SM contraction  Negative regulation of apoptotic process  Folding of actin and tubulin  Actin cytoskeleton organization  Acetyl-CoA biosynthetic process from pyruvate  Actin cytoskeleton organization  Actin cytoskeleton organization  Regulation of the macrophages’ function  Actin crosslink formation  Acetyl-CoA biosynthetic process from pyruvate  Degradation of ubiquitinated proteins  Positive regulation of translation  Microtubule cytoskeleton organization  Mitochondrion organization/protein quality control  Skeletal muscle thin filament assembly  Stabilization of microvessels/muscle cells  Microtubule cytoskeleton organization  Fatty acid beta-oxidation  Mitochondrial electron transport, succinate to ubiquinone  Cellular calcium ion homeostasis  Fatty acid transport/beta-oxidation  Leucine catabolic process  Regulation of MAPK cascade  Regulation of mitochondrial respiration and mitochondrial DNA copy number maintenance  Fatty acid transport/beta-oxidation  Regulation of mitochondrial membrane potential  Intra-Golgi protein transport  Regulator of lipid metabolism  ATPase activity/ localized to the mitochondrion  Fatty acid beta-oxidation  Hydrogen sulfide catabolism  Fatty acid beta-oxidation  Acylglycerol catabolic process  Androgen receptor signalling  Epimerization of S-/R-forms of NAD(P)HX  Valine catabolic process  Cellular calcium ion homeostasis  Protein import into mitochondrial matrix  Detoxification processes  Mitochondrial electron transport, NADH to ubiquinone  Breakdown of odd-numbered fatty acids and the amino acids: valine, isoleucine  Production of acetic acid from acetyl phosphate  Adenine nucleotide metabolism  Antioxidant NADPH oxidoreductase supporting oxidative phosphorylation  L-phenylalanine/tyrosine catabolic process  Dimerization of the ATP synthase complex  Proton motive force-driven mitochondrial ATP synthesis  Heme biosynthetic pathway  Protein quality control for misfolded or incompletely synthesized proteins  Regulation of mitochondrion organization  Cytoplasmic translation |

116 statistically significant proteins out of 1203 included in the analysis (82 up-regulated, 34 down-regulated). Proteins related to metabolism are marked in purple. Note: some of the entities were slightly above/below FC threshold but were retained in the dataset due to biological importance.

**Sup. Table 3.** Differentially expressed proteins (DEPs) in the dataset: **8-month-old Tgαq*44 vs. age-matched control FVB mice** (fold change (Tgαq*44 /Control) ≥1.2 or ≤0.8 and adjusted *p* value <0.05); *n* = 7/group.

| No. | Accession  number | Annotation | FC (Tg/Ctrl) | Molecular Function |
| --- | --- | --- | --- | --- |
| 1  2  3  4  5  6  7  8  9  10  11  12  13  14  15  16  17  18  19  20  21  22  23  24  25  26  27  28  29  30  31  32  33  34  35  36  37  38  39  40  41  42  43  44  45  46  47  48  49  50  51  52  53  54  55  56  57  58  59  60  61  62  63  64  65  66  67  68  69  70  71  72  73  74  75  76  77  78  79  80  81  82  83  84  85  86  87  88  89  90  91  92  93  94  95  96  97  98  99  100  101  102  103  104  105  106  107  108  109  110  111  112  113  114  115  116  117  118  119  120  121  122  123  124  125  126  127  128  129  130  131  132  133  134  135  136  137  138  139  140  141  142  143  144  145  146  147  148  149  150  151  152  153  154  155  156  157  158  159  160  161  162  163  164  165  166  167  168  169  170  171  172  173  174  175  176  177  178  179  180  181  182  183  184  185  186  187  188  189  190  191  192  193  194  195  196  197  198  199  200  201  202  203  204  205  206  207  208  209  210  211  212  213  214  215  216  217  218  219  220  221  222  223  224  225  226  227  228  229  230  231  232  233  234  235  236 | P24549  Q8BMK4  Q99P72  Q62465  Q8BH61  P62264  P68037  P60766  O55131  Q9WTQ5  Q8BFW7  P25444  Q9JK53  Q920M5  Q62188  Q9EQK5  P46412  Q8VDJ3  P42208  P37040  Q80XB4  O54931  Q9Z2X1  Q07076  O88544  P58389  Q62009  P80316  P63323  Q9D0R2  P13020  P51885  Q78J03  Q80X90  Q6URW6  Q9D7X3  P20918  P63024  P09103  P05125  Q6PHZ2  Q91VI7  Q8BTM8  Q62448  Q9JII6  O55003  Q9D8Y0  Q61207  P62071  Q922R8  Q8CGC7  P51125  P14602  P48678  Q91VH6  P19324  Q99L04  Q61554  P82347  Q8BKC5  Q9CPV4  Q9R0P5  P14685  Q9EPM5  O55026  Q8CIB5  P40124  Q9WVA4  O35887  Q9QZ08  Q9CZN7  P31428  P28653  Q8BGQ7  Q5XJY5  P46935  Q61292  P32261  P35564  Q8BG05  Q9DCN2  O70400  Q02788  P11276  P28665  Q8BG32  P28650  P26041  P42932  Q3URD3  Q91Z83  P48722  Q9EQP2  Q8BWB1  O08529  Q9ESD7  P14733  P20152  O08677  P61971  O08638  P14211  Q8VDD5  Q9D1L0  Q3UTJ2  Q9WUA3  P23116  P10493  O70373  P31001  P97351  P24527  Q99KC8  Q8CGK3  Q7TPW1  Q65CL1  Q9R1P0  Q99JI4  O09131  Q8C1B7  P27773  Q921I1  Q8BRK8  Q922B2  P01027  Q61316  Q8BZF8  Q9R0X4  P48036  P26039  P40142  P46471  Q8VHX6  P58252  Q9DBG3  P26231  P97447  P09055  Q7TQI3  P11983  Q19LI2  Q7M6Y3  O35639  Q9Z2W0  P61161  P60843  O08547  P07901  Q91X72  P24452  Q3V384  P24270  P15116  Q68FD5  P27546  P11499  P14206  E9Q557  Q61838  Q9QXS1  P20029  P14869  Q9JHU4  Q91YT0  Q60597  Q9D051  P14152  Q924X2  P52825  P09542  Q9CZU6  Q9D6J6  Q9CZ13  Q8CAQ8  P21550  P41216  Q9JK42  Q922B1  P19123  Q01768  P54071  Q9QYR9  P47934  E9Q401  Q9ERS2  Q8CHT0  Q91VD9  O08749  Q9CQ62  P63094  Q8BWT1  O35459  P50544  Q9D8B4  Q8BMS1  Q9JHI5  Q9Z1P6  Q8CC88  P24472  Q60936  O54734  Q9CPU4  Q3UM45  Q99JY0  P53986  O35678  Q9D880  Q62425  P42125  Q8CAK1  Q99L13  P50171  Q9D1I5  Q8C0M9  Q71RI9  P53395  Q2TPA8  Q05BC3  Q8QZR5  Q9DC70  Q8BYM8  Q8BK64  Q9WVL0  Q9JM76  Q8BH86  Q8BWF0  Q8VCW8  Q99J99  Q8VCT4  Q8BW75  P22315  Q99J39  Q8K411  Q8C5H8  Q8R0N6  P35505 | Aldehyde dehydrogenase 1A1  Cytoskeleton-associated protein 4  Reticulon-4  Synaptic vesicle membrane protein VAT-1 homolog  Coagulation factor XIII A chain  40S ribosomal protein S14  Ubiquitin-conjugating enzyme E2 L3  Cell division control protein 42 homolog  Septin-7  A-kinase anchor protein 12  Lipoma-preferred partner homolog  40S ribosomal protein S2  Prolargin  Coronin-6  Dihydropyrimidinase-related protein 3  Major vault protein  Glutathione peroxidase 3  Vigilin  Septin-2  NADPH—cytochrome P450 reductase  Nebulin-related-anchoring protein  A-kinase anchor protein 2  Heterogeneous nuclear ribonucleoprotein F  Annexin A7  COP9 signalosome complex subunit 4  Serine/threonine-protein phosphatase 2A activator  Periostin  T-complex protein 1 subunit epsilon  40S ribosomal protein S12  Threonine—tRNA ligase 1, cytoplasmic  Gelsolin  Lumican  Methionine-R-sulfoxide reductase B2, mitochondrial  Filamin-B  Myosin-14  Dual specificity protein phosphatase 3  Plasminogen  Vesicle-associated membrane protein 3  Protein disulfide-isomerase (PDIA1)  Natriuretic peptides A  Calcium/calmodulin-dependent protein kinase type II subunit delta  Ribonuclease inhibitor  Filamin-A  Eukaryotic translation initiation factor 4 gamma 2 Aldo-keto reductase family 1 member A1 BCL2/adenovirus E1B 19 kDa protein interacting protein 3  EF-hand domain-containing protein D2  Prosaposin  Ras-related protein R-Ras2  Protein disulfide-isomerase A6 (PDIA6)  Bifunctional glutamate/proline—tRNA ligase  Calpastatin  Heat shock protein beta-1  Prelamin-A/C  Protein MEMO1  Serpin H1  Dehydrogenase/reductase SDR family member 1  Fibrillin-1  Delta-sarcoglycan  Importin-5  Glyoxalase domain-containing protein 4  Destrin  26S proteasome non-ATPase regulatory subunit 3  Syncoilin  Ectonucleoside triphosphate diphosphohydrolase 2  Fermitin family homolog 2  Adenylyl cyclase-associated protein 1  Transgelin-2  Calumenin  N-acetyl-D-glucosamine kinase  Serine hydroxymethyltransferase, mitochondrial  Dipeptidase 1  Biglycan  Alanine—tRNA ligase, cytoplasmic  Coatomer subunit delta  E3 ubiquitin-protein ligase NEDD4  Laminin subunit beta-2  Antithrombin-III  Calnexin  Heterogeneous nuclear ribonucleoprotein A3  NADH-cytochrome b5 reductase 3  PDZ and LIM domain protein 1  Collagen alpha-2(VI) chain  Fibronectin  Murinoglobulin-1  26S proteasome non-ATPase regulatory subunit 11  Adenylosuccinate synthetase isozyme 1  Moesin  T-complex protein 1 subunit theta  Sarcolemmal membrane-associated protein  Myosin-7  Heat shock 70 kDa protein 4L  EH domain-containing protein 4  Synaptopodin 2-like protein  Calpain-2 catalytic subunit  Dysferlin  Lamin-B1  Vimentin  Kininogen-1  Nuclear transport factor 2  Myosin-11  Calreticulin  Myosin-9  Coiled-coil-helix-coiled-coil-helix domain-containing protein 2  Sorbin and SH3 domain-containing protein 2  ATP-dependent 6-phosphofructokinase, platelet type  Eukaryotic translation initiation factor 3 subunit A  Nidogen-1  Xin actin-binding repeat-containing protein 1  Desmin  40S ribosomal protein S3a  Leukotriene A-4 hydrolase  von Willebrand factor A domain-containing protein 5A  Lon protease homolog, mitochondrial  Nexilin  Catenin alpha-3  Proteasome subunit alpha type-4  26S proteasome non-ATPase regulatory subunit 6  Glutathione S-transferase omega-1  Septin-11  Protein disulfide-isomerase A3 (PDIA3)  Serotransferrin  5’-AMP-activated protein kinase catalytic subunit alpha-2  Aspartate—tRNA ligase, cytoplasmic  Complement C3  Heat shock 70 kDa protein 4  Phosphoglucomutase-like protein 5  Acyl-coenzyme A thioesterase 9, mitochondrial  Annexin A5  Talin-1  Transketolase  26S proteasome regulatory subunit 7  Filamin-C  Elongation factor 2  AP-2 complex subunit beta  Catenin alpha-1  Four and a half LIM domains protein 1  Integrin beta-1  Ubiquitin thioesterase OTUB1  T-complex protein 1 subunit alpha  Alpha-1B-glycoprotein  Phosphatidylinositol-binding clathrin assembly protein  Annexin A3  Aspartyl aminopeptidase  Actin-related protein 2  Eukaryotic initiation factor 4A-I  Vesicle-trafficking protein SEC22b  Heat shock protein HSP 90-alpha  Hemopexin  Macrophage-capping protein  AFG1-like ATPase  Catalase  Cadherin-2  Clathrin heavy chain 1  Microtubule-associated protein 4  Heat shock protein HSP 90-beta  40S ribosomal protein SA  Desmoplakin  Pregnancy zone protein  Plectin  Endoplasmic reticulum chaperone BiP  60S acidic ribosomal protein P0  Cytoplasmic dynein 1 heavy chain 1  NADH dehydrogenase [ubiquinone] flavoprotein 1, mitochondrial  2-oxoglutarate dehydrogenase complex component E1  Pyruvate dehydrogenase E1 component subunit beta, mitochondrial  Malate dehydrogenase, cytoplasmic  Carnitine O-palmitoyltransferase 1, muscle isoform  Carnitine O-palmitoyltransferase 2, mitochondrial  Myosin light chain 3  Citrate synthase, mitochondrial  NADH dehydrogenase [ubiquinone] flavoprotein 2, mitochondrial  Cytochrome b-c1 complex subunit 1, mitochondrial  MICOS complex subunit Mic60  Beta-enolase  Long-chain-fatty-acid—CoA ligase 1    [Pyruvate dehydrogenase (acetyl-transferring)] kinase isozyme 2, mitochondrial  ADP-ribose glycohydrolase MACROD1  Troponin C, slow skeletal and cardiac muscles  Nucleoside diphosphate kinase B  Isocitrate dehydrogenase [NADP], mitochondrial  Acyl-coenzyme A thioesterase 2, mitochondrial  Carnitine O-acetyltransferase  Ryanodine receptor 2  NADH dehydrogenase [ubiquinone] 1 alpha subcomplex subunit 13  Delta-1-pyrroline-5-carboxylate dehydrogenase, mitochondrial  NADH-ubiquinone oxidoreductase 75 kDa subunit, mitochondrial  Dihydrolipoyl dehydrogenase, mitochondrial  2,4-dienoyl-CoA reductase [(3E)-enoyl-CoA-producing], mitochondrial  Guanine nucleotide-binding protein G(s) subunit alpha isoforms short  3-ketoacyl-CoA thiolase, mitochondrial  Delta(3,5)-Delta(2,4)-dienoyl-CoA isomerase, mitochondrial  Very long-chain specific acyl-CoA dehydrogenase, mitochondrial  NADH dehydrogenase [ubiquinone] 1 alpha subcomplex subunit 11  Trifunctional enzyme subunit alpha, mitochondrial  Isovaleryl-CoA dehydrogenase, mitochondrial  NADH dehydrogenase [ubiquinone] 1 alpha subcomplex subunit 7  von Willebrand factor A domain-containing protein 8  Glutathione S-transferase A4  Atypical kinase COQ8A, mitochondrial  Dolichyl-diphosphooligosaccharide—protein glycosyltransferase 48 kDa subunit  Microsomal glutathione S-transferase 3  Protein phosphatase 1 regulatory subunit 7  Trifunctional enzyme subunit beta, mitochondrial  Monocarboxylate transporter 1  Monoglyceride lipase  Mitochondrial import inner membrane translocase subunit TIM50  Cytochrome c oxidase subunit NDUFA4  Enoyl-CoA delta isomerase 1, mitochondrial  Putative transferase CAF17 homolog, mitochondrial  3-hydroxyisobutyrate dehydrogenase, mitochondrial  (3R)-3-hydroxyacyl-CoA dehydrogenase  Methylmalonyl-CoA epimerase, mitochondrial  Isoaspartyl peptidase/L-asparaginase  Kynurenine—oxoglutarate transaminase 3  Lipoamide acyltransferase component of branched-chain alpha-keto acid dehydrogenase complex, mitochondrial  Hydroxysteroid dehydrogenase-like protein 2  Echinoderm microtubule-associated protein-like 1  Alanine aminotransferase 1  NADH dehydrogenase [ubiquinone] iron-sulfur protein 7, mitochondrial  Probable cysteine--tRNA ligase, mitochondrial  Activator of 90 kDa heat shock protein ATPase homolog 1  Maleylacetoacetate isomerase  Actin-related protein 2/3 complex subunit 3  D-glutamate cyclase, mitochondrial  Succinate-semialdehyde dehydrogenase, mitochondrial  Medium-chain acyl-CoA ligase ACSF2, mitochondrial  3-mercaptopyruvate sulfurtransferase  Carboxylesterase 1D  Amine oxidase [flavin-containing] B  Ferrochelatase, mitochondrial  Malonyl-CoA decarboxylase, mitochondrial  Presequence protease, mitochondrial  NAD kinase 2, mitochondrial  Hydroxyacid-oxoacid transhydrogenase, mitochondrial  Fumarylacetoacetase | 5.87  5.57  5.2  4.8  4.4  3.9  3.85  3.76  3.55  3.5  3.5  3.5  3.4  3.37  3.33  3.26  3.26  3  3  3  2.94  2.92  2.9  2.89  2.85  2.79  2.78  2.74  2.72  2.7  2.56  2.53  2.51  2.5  2.5  2.48  2.47  2.42  2.38  2.32  2.31  2.28  2.27  2.25  2.23  2.23  2.22  2.21  2.21  2.21  2.2  2.2  2.2  2.2  2.19  2.19  2.15  2.14  2.14  2.14  2.14  2.14  2.13  2.12  2.12  2.11  2.11  2.1  2.08  2.08  2.08  2.07  2  2  2  2  2  1.97  1.96  1.93  1.91  1.88  1.87  1.87  1.86  1.83  1.83  1.82  1.81  1.8  1.79  1.78  1.78  1.77  1.76  1.76  1.76  1.76  1.76  1.75  1.75  1.74  1.73  1.72  1.71  1.71  1.69  1.67  1.67  1.64  1.63  1.63  1.62  1.62  1.61  1.61  1.61  1.6  1.6  1.59  1.58  1.58  1.57  1.56  1.56  1.55  1.55  1.55  1.54  1.54  1.54  1.53  1.5  1.5  1.5  1.5  1.47  1.46  1.43  1.42  1.42  1.42  1.41  1.41  1.41  1.38  1.38  1.37  1.37  1.37  1.36  1.36  1.35  1.34  1.31  1.30  1.29  1.29  1.28  1.26  1.26  1.25  1.24  0.91  0.88  0.87  0.86  0.86  0.84  0.83  0.83  0.82  0.82  0.82  0.82  0.82  0.82  0.80  0.79  0.77  0.77  0.76  0.75  0.75  0.74  0.74  0.74  0.74  0.73  0.73  0.73  0.71  0.71  0.7  0.7  0.7  0.69  0.69  0.68  0.68  0.65  0.65  0.64  0.64  0.63  0.62  0.62  0.62  0.62  0.61  0.61  0.6  0.6  0.58  0.58  0.56  0.54  0.5  0.5  0.5  0.48  0.48  0.46  0.45  0.45  0.45  0.44  0.41  0.38  0.37  0.37  0.37  0.35  0.32  0.31  0.16 | Cellular detoxification of aldehydes  Anchoring of the ER to microtubules  Promoting tubular ER production  Negative regulation of mitochondrial fusion  Blood coagulation  Cytoplasmic translation  Regulation of protein ubiquitination  Actin cytoskeleton organization  Sarcomere structural organization  Regulation of protein kinase A/C signalling  Cell-cell adhesion  Cytoplasmic translation  Anchoring of basement membranes to the underlying connective tissue  Actin filament organization  Actin crosslink formation  Regulation of signal transduction through JAK/MAPK  Response to oxidative stress  Protecting cells from over-accumulation of cholesterol  Cytoskeleton-dependent cytokinesis  Detoxification processes  Actin cytoskeleton organization  Cardiac response to stress  mRNA processing  Membrane fusion/involved in exocytosis  Protein deneddylation  Regulation of protein dephosphorylation  Cell adhesion  Chaperone-mediated protein folding  Cytoplasmic translation  tRNA aminoacylation for protein translation  Actin cytoskeleton organization  Collagen cross-linking  Response to oxidative stress  Actin cytoskeleton organization  Actomyosin structure organization  Negative regulation of ERK1/ERK2 cascade  Blood coagulation  Retrograde transport, endosome to Golgi  Protein folding  Cardiac muscle hypertrophy in response to stress  Calcium ion transport/Relaxation of cardiac muscle  Regulation of redox homeostasis  Actin crosslink formation  Regulation of translational initiation  Cellular detoxification of aldehydes  Regulation of autophagy of mitochondrion  Controlling spontaneous apoptosis  Ceramide metabolic process  Ras protein signal transduction  Aggregation of misfolded proteins  Glutamyl/prolyl-tRNA aminoacylation  Protein catabolic process  Chaperone-mediated protein folding  Regulation of cardiac muscle hypertrophy  Regulation of microtubule-based process  Collagen biosynthetic process  Steroid and/or xenobiotic metabolism  Component of calcium-binding microfibrils  Cardiac muscle cell contraction  Protein import into nucleus  Cadherin binding  Actin filament depolymerization  Ubiquitin-dependent protein catabolic process  Intermediate filament-based process  Nucleoside diphosphate catabolic process  Integrin-mediated signalling pathway  Actin cytoskeleton organization  Actin dynamics’ regulation/stabilization actin structures  Calcium-binding protein contributing to ER protein sorting and folding  N-acetylneuraminate catabolic process  Interconversion of serine and glycine  Conversion of leukotriene D4 to E4  Peptide cross-linking via chondroitin 4-sulfate glycosaminoglycan  Alanyl-tRNA aminoacylation  Retrograde vesicle-mediated transport, Golgi to endoplasmic reticulum  Proteasome-mediated ubiquitin-dependent protein catabolic process  Positive regulation of muscle cell differentiation  Blood coagulation  Clathrin-dependent endocytosis  mRNA splicing  Desaturation and elongation of fatty acids, cholesterol biosynthesis  Actin cytoskeleton organization  Cell adhesion  Positive regulation of substrate-dependent cell migration, cell attachment to substrate  Negative regulation of peptidase activity  Ubiquitin-dependent protein catabolic process  'De novo' AMP biosynthetic process  Mediation in connections of cytoskeletal structures to the plasma membrane  Chaperone-mediated protein folding  Protein localization to plasma membrane  Regulation of the force of heart contraction  Protein folding  Endosomal transport  Regulation of actin filament bundle assembly  Proteolysis of substrates involved in cytoskeletal remodelling/signal transduction  Sarcolemma repairing of both skeletal muscle and cardiomyocytes  Nuclear envelope organization  Intermediate filament organization  Blood coagulation/vasodilation  Regulation of protein import into nucleus  Actomyosin structure organization  Promoting protein folding, oligomeric assembly and quality control in the ER  Actin cytoskeleton reorganization  Cellular response to oxidative stress  Maintaining structural integrity in cardiomyocytes  Canonical glycolysis  Formation of cytoplasmic translation initiation complex  Cell-matrix adhesion  Actin filament organization  Intermediate filament organization  Cytoplasmic translation  Leukotriene biosynthetic process  A role in tumorigenesis  Protein quality control for misfolded or incompletely synthesized proteins  Regulation of cytoskeleton organization  Formation of stretch-resistant cell- cell adhesion complexes  Ubiquitin-dependent protein catabolic process  Proteasome-mediated ubiquitin-dependent protein catabolic process  Modulation of critical inflammatory pathways/cellular defence  Cytoskeleton-dependent cytokinesis  Protein folding  Ferroptosis pathway/iron ion transport  Regulation of cellular energy metabolism  Aspartyl-tRNA aminoacylation  Activation of the complement system  Chaperone-mediated protein complex assembly  Cell adhesion  Acyl-CoA metabolic process/major mitochondrial ACOT  Blood coagulation cascade  Connection of major cytoskeletal structures to the plasma membrane  Non-oxidative branch of the pentose phosphate pathway  Proteasome-mediated ubiquitin-dependent protein catabolic process  Maintenance of the structural integrity of the muscle fibers  Translational elongation  Clathrin-dependent endocytosis  Association with the cytoplasmic domain of a variety of cadherins/cell adhesion  Cell differentiation  Cell-cell adhesion  Protein deubiquitination  Chaperone-mediated protein folding  Cell recognition/regulation of cell behaviour  AP2-dependent clathrin-mediated endocytosis  Anti-coagulant activity  Intracellular protein and peptide metabolism  Actin cytoskeleton organization  Cytoplasmic translational initiation  Vesicle-mediated transport (Golgi/ER)  Chaperone-mediated protein complex assembly  Heme metabolic process  Barbed-end actin filament capping  Mitochondrial protein homeostasis  Cellular detoxification of hydrogen peroxide  Calcium-dependent cell-cell adhesion  Clathrin-dependent endocytosis  Microtubule cytoskeleton organization  Chaperone-mediated protein complex assembly  Translation/cell-cell adhesion  Adherens junction organization  Negative regulation of peptidase activity  Intermediate filament cytoskeleton organization  Folding of proteins and degradation of misfolded proteins  Cytoplasmic translation  Cytoplasmic microtubule organization  Mitochondrial electron transport, NADH to ubiquinone  Conversion of 2-oxoglutarate to succinyl-CoA  Acetyl-CoA biosynthetic process from pyruvate  NAD/NADH-dependent interconversion of malate and oxaloacetate  Long-chain fatty acid transport  Long-chain fatty acid transport  Cardiac muscle contraction  Tricarboxylic acid cycle  Mitochondrial electron transport, NADH to ubiquinone  Mitochondrial electron transport, ubiquinol to cytochrome c  Maintenance of the MICOS complex stability and the mitochondrial cristae morphology  Canonical glycolysis  Activation of long-chain fatty acids for degradation via beta-oxidation  Inhibition of pyruvate dehydrogenase activity  Peptidyl-glutamate ADP-deribosylation  Cardiac muscle contraction  Synthesis of nucleoside triphosphates other than ATP  Tricarboxylic acid cycle  Acyl-CoA metabolic process/active on substrates with chains: C14-C20  Interconversion of acetyl-CoA and acetylcarnitine/positive regulator of PDH  Calcium ion transmembrane transport into cytosol  Transfer of electrons from NADH to the respiratory chain  Proline catabolic process to glutamate  Mitochondrial electron transport, NADH to ubiquinone  Regulation of pyruvate dehydrogenase (PDH) complex/pyruvate metabolism  Fatty acid beta-oxidation  Adenylate cyclase-modulating G protein-coupled receptor signalling pathway  Fatty acid beta-oxidation  Fatty acid beta-oxidation  Fatty acid beta-oxidation  Transfer of electrons from NADH to the respiratory chain  Fatty acid beta-oxidation  Leucine catabolic process  Transfer of electrons from NADH to the respiratory chain  ATP hydrolysis  Detoxification of electrophilic compounds by conjugation with glutathione  Biosynthesis of coenzyme Q  Protein N-linked glycosylation via asparagine  Leukotriene biosynthetic process  Regulation of protein dephosphorylation  Fatty acid beta-oxidation  Transport across the plasma membrane of monocarboxylates (lactate, pyruvate, ketones)  Monoacylglycerol catabolic process  Protein import into mitochondrial matrix  Terminal component of the mitochondrial respiratory chain/complex IV maintenance  Fatty acid beta-oxidation  Maturation of mitochondrial 4Fe-4S proteins  Valine catabolic process  Fatty acid biosynthetic process  Degradation of branched amino acids and odd-chain fatty acids  Asparagine catabolic process via L-aspartate  L-kynurenine catabolic process  Branched-chain amino acid catabolic process  Lipid metabolism regulator  Microtubule cytoskeleton organization  Reversible transamination between alanine and 2-oxoglutarate to form pyruvate and glutamate  Mitochondrial electron transport, NADH to ubiquinone  Cysteinyl-tRNA aminoacylation  Activation of the ATPase activity of HSP90AA1  L-phenylalanine/tyrosine catabolic process  Regulation of actin filament polymerization  Conversion of D-glutamate to 5-oxo-D-proline/accumulation of D-glutamate  Gamma-aminobutyric acid catabolic process  Medium-chain fatty acid activation  Hydrogen sulfide biosynthetic process  Acylglycerol catabolic process  Oxidative deamination of biogenic and xenobiotic amines  Heme biosynthetic process  Positive regulation of fatty acid oxidation  Degradation of mitochondrial transit peptides after their cleavage  NADP biosynthetic process  Reversible oxidation of gamma-hydroxybutyrate to acetoacetate  L-phenylalanine/tyrosine catabolic process |

236 statistically significant proteins out of 1324 included in the analysis (163 up-regulated, 73 down-regulated). Proteins related to metabolism are marked in purple. Note: some of the entities were slightly above/below FC threshold but were retained in the dataset due to biological importance.

**Sup. Table 4.** Differentially expressed proteins (DEPs) in the dataset: **10-month-old Tgαq*44 vs. age-matched control FVB mice** (fold change (Tgαq*44 /Control) ≥1.2 or ≤0.8 and adjusted *p* value <0.05); *n* = 5/group.

| No. | Accession  number | Annotation | FC (Tg/Ctrl) | Molecular Function |
| --- | --- | --- | --- | --- |
| 1  2  3  4  5  6  7  8  9  10  11  12  13  14  15  16  17  18  19  20  21  22  23  24  25  26  27  28  29  30  31  32  33  34  35  36  37  38  39  40  41  42  43  44  45  46  47  48  49  50  51  52  53  54  55  56  57  58  59  60  61  62  63  64  65  66  67  68  69  70  71  72  73  74  75  76  77  78  79  80  81  82  83  84  85  86  87  88  89  90  91  92  93  94  95  96  97  98  99  100  101  102  103  104  105  106  107  108  109  110  111  112  113  114  115  116  117  118  119  120  121  122  123  124  125  126  127  128  129  130  131  132  133  134  135  136  137  138  139  140  141  142  143  144  145  146  147  148  149  150  151  152  153  154  155  156  157  158  159  160  161  162  163  164  165  166  167  168  169  170  171  172  173  174  175  176  177  178  179  180  181  182  183  184  185  186  187  188  189  190 | P42208  Q9R0X4  P19324  O70435  Q99JY9  Q61738  Q9JMH6  Q80XB4  Q8BWB1  Q99KC8  Q9DBG3  P21619  Q9D8Y0  Q99LD8  P10107  Q8CDN6  Q61543  O88487  Q9EQK5  P14602  Q8BSY0  Q9EQP2  P63276  Q9CR42  Q9DCN2  Q9WVB0  Q9JK53  P17742  Q07076  P97447  Q3URD3  Q7TPW1  P40124  Q9WVA4  O70373  Q3TXS7  Q80X90  Q9ET54  P20918  P26039  O70400  Q8CGC7  Q9ESD7  P08752  Q7TMM9  Q8BTM8  P56395  P62830  Q9CQB5  Q8K4L3  Q62448  Q61292  Q8VDD5  Q6P069  Q9CQW2  O55131  Q9D1H6  P62082  P35564  Q9Z0P5  Q7M6Y3  A2AUC9  Q64010  P27773  Q61554  O08529  P09103  Q9D0E1  P62334  P42932  Q9WTI7  Q68FD5  Q64516  P24369  P48774  Q8CGK3  P26041  Q02788  P60843  P60335  P28654  Q9WUA3  Q91Z83  P51885  Q61207  P17710  Q02053  Q04447  P99024  Q3UTJ2  P26231  P60766  Q8VHX6  Q9D1X0  Q60668  Q9CQ60  Q61147  O08600  Q9JMC3  P23116  P20152  P68372  Q3UH68  Q71LX4  P48678  Q04857  O88342  Q8BH64  Q9QXS1  P27546  O08553  Q9Z2U1  P80316  Q9JLV1  Q3UIU2  Q9D6U8  Q64727  P62962  P16381  O08528  Q9R0P3  Q8CI51  Q99020  Q05793  P57780  P16546  Q01768  Q62261  P63101  P16125  P45376  P42125  Q8VDN2  Q8BMS1  Q91YT0  P41216  Q9Z2I9  Q9DB77  Q8QZT1  Q8R1S0  Q61941  P50171  Q924X2  Q03265  Q9CQ54  P17563  P24472  Q99MN9  Q9JHI5  Q9CQN1  P47934  Q9QYR9  P51174  P45952  Q8BWT1  P50544  Q60936  P53986  Q8K0E8  Q9EQ20  Q99L13  Q9DBG5  Q4VAE3  P58771  E9Q401  Q9WV98  Q9CQ91  P50136  P52825  Q922B1  O55234  Q8CC88  Q9WUZ7  Q9Z2Z6  Q8VCW8  Q811I0  Q99M87  P40630  Q60675  Q8BW75  P62869  Q9JLZ3  Q2TPA8  Q9DCM2  Q9JJI8  O35215  P70414  Q9Z1J3  Q71RI9  P35505 | Septin-2  Acyl-coenzyme A thioesterase 9, mitochondrial  Serpin H1  Proteasome subunit alpha type-3  Actin-related protein 3  Integrin alpha-7  Thioredoxin reductase 1, cytoplasmic  Nebulin-related-anchoring protein  Synaptopodin 2-like protein  von Willebrand factor A domain-containing protein 5A  AP-2 complex subunit beta  Lamin-B2  EF-hand domain-containing protein D2  N(G),N(G)-dimethylarginine dimethylaminohydrolase 2  Annexin A1  Thioredoxin-like protein 1  Golgi apparatus protein 1  Cytoplasmic dynein 1 intermediate chain 2  Major vault protein  Heat shock protein beta-1  Aspartyl/asparaginyl beta-hydroxylase  EH domain-containing protein 4  40S ribosomal protein S17  Ankyrin repeat domain-containing protein 1  NADH-cytochrome b5 reductase 3  RNA-binding protein with multiple splicing  Prolargin  Peptidyl-prolyl cis-trans isomerase A  Annexin A7  Four and a half LIM domains protein 1  Sarcolemmal membrane-associated protein  Nexilin  Adenylyl cyclase-associated protein 1  Transgelin-2  Xin actin-binding repeat-containing protein 1  26S proteasome non-ATPase regulatory subunit 1  Filamin-B  Palladin  Plasminogen  Talin-1  PDZ and LIM domain protein 1  Bifunctional glutamate/proline--tRNA ligase  Dysferlin  Guanine nucleotide-binding protein G(i) subunit alpha-2  Tubulin beta-2A chain  Filamin-A  Cytochrome b5  60S ribosomal protein L23  CDGSH iron-sulfur domain-containing protein 2  Supervillin  Eukaryotic translation initiation factor 4 gamma 2  Laminin subunit beta-2  Myosin-9  Sorcin  ADP-ribosylation factor-like protein 8B  Septin-7  NADH dehydrogenase [ubiquinone] 1 alpha subcomplex assembly factor 4  40S ribosomal protein S7  Calnexin  Twinfilin-2  Phosphatidylinositol-binding clathrin assembly protein  Kelch-like protein 41  Adapter molecule crk  Protein disulfide-isomerase A3 (PDIA3)  Fibrillin-1  Calpain-2 catalytic subunit  Protein disulfide-isomerase (PDIA1)  Heterogeneous nuclear ribonucleoprotein M  26S proteasome regulatory subunit 10B  T-complex protein 1 subunit theta  Unconventional myosin-Ic  Clathrin heavy chain 1  Glycerol kinase  Peptidyl-prolyl cis-trans isomerase B  Glutathione S-transferase Mu 5  Lon protease homolog, mitochondrial  Moesin  Collagen alpha-2(VI) chain  Eukaryotic initiation factor 4A-I  Poly(rC)-binding protein 1  Decorin  ATP-dependent 6-phosphofructokinase, platelet type  Myosin-7  Lumican  Prosaposin  Hexokinase-1  Ubiquitin-like modifier-activating enzyme 1  Creatine kinase B-type  Tubulin beta-5 chain  Sorbin and SH3 domain-containing protein 2  Catenin alpha-1  Cell division control protein 42 homolog  Filamin-C  Nucleolar protein 3  Heterogeneous nuclear ribonucleoprotein D0  6-phosphogluconolactonase  Ceruloplasmin  Endonuclease G, mitochondrial  DnaJ homolog subfamily A member 4  Eukaryotic translation initiation factor 3 subunit A  Vimentin  Tubulin beta-4B chain  LIM and calponin homology domains-containing protein 1  Talin-2  Prelamin-A/C  Collagen alpha-1(VI) chain  WD repeat-containing protein 1  EH domain-containing protein 2  Plectin  Microtubule-associated protein 4  Dihydropyrimidinase-related protein 2  Proteasome subunit alpha type-5  T-complex protein 1 subunit epsilon  BAG family molecular chaperone regulator 3  NADH dehydrogenase [ubiquinone] 1 beta subcomplex subunit 6  Protein FAM162A  Vinculin  Profilin-1  Putative ATP-dependent RNA helicase Pl10  Hexokinase-2  S-formylglutathione hydrolase  PDZ and LIM domain protein 5  Heterogeneous nuclear ribonucleoprotein A/B  Basement membrane-specific heparan sulfate proteoglycan core protein  Alpha-actinin-4  Spectrin alpha chain, non-erythrocytic 1  Nucleoside diphosphate kinase B  Spectrin beta chain, non-erythrocytic 1  14-3-3 protein zeta/delta  L-lactate dehydrogenase B chain  Aldo-keto reductase family 1 member B1  Enoyl-CoA delta isomerase 1, mitochondrial  Sodium/potassium-transporting ATPase subunit alpha-1  Trifunctional enzyme subunit alpha, mitochondrial  NADH dehydrogenase [ubiquinone] flavoprotein 1, mitochondrial  Long-chain-fatty-acid--CoA ligase 1  Succinate--CoA ligase [ADP-forming] subunit beta, mitochondrial  Cytochrome b-c1 complex subunit 2, mitochondrial  Acetyl-CoA acetyltransferase, mitochondrial  Ubiquinone biosynthesis monooxygenase COQ6, mitochondrial  NAD(P) transhydrogenase, mitochondrial  (3R)-3-hydroxyacyl-CoA dehydrogenase  Carnitine O-palmitoyltransferase 1, muscle isoform  ATP synthase subunit alpha, mitochondrial  NADH dehydrogenase [ubiquinone] 1 subunit C2  Methanethiol oxidase  Glutathione S-transferase A4  Propionyl-CoA carboxylase beta chain, mitochondrial  Isovaleryl-CoA dehydrogenase, mitochondrial  Heat shock protein 75 kDa, mitochondrial  Carnitine O-acetyltransferase  Acyl-coenzyme A thioesterase 2, mitochondrial  Long-chain specific acyl-CoA dehydrogenase, mitochondrial  Medium-chain specific acyl-CoA dehydrogenase, mitochondrial  3-ketoacyl-CoA thiolase, mitochondrial  Very long-chain specific acyl-CoA dehydrogenase, mitochondrial  Atypical kinase COQ8A, mitochondrial  Monocarboxylate transporter 1  Fibrinogen beta chain  Methylmalonate-semialdehyde dehydrogenase [acylating], mitochondrial  3-hydroxyisobutyrate dehydrogenase, mitochondrial  Perilipin-3  Transmembrane protein 65  Tropomyosin alpha-1 chain  Ryanodine receptor 2  Mitochondrial import inner membrane translocase subunit Tim9  NADH dehydrogenase [ubiquinone] 1 alpha subcomplex subunit 3  2-oxoisovalerate dehydrogenase subunit alpha, mitochondrial  Carnitine O-palmitoyltransferase 2, mitochondrial  ADP-ribose glycohydrolase MACROD1  Proteasome subunit beta type-5  von Willebrand factor A domain-containing protein 8  SH3 domain-binding glutamic acid-rich protein  Mitochondrial carnitine/acylcarnitine carrier protein  Medium-chain acyl-CoA ligase ACSF2, mitochondrial  ATP synthase mitochondrial F1 complex assembly factor 1  DnaJ homolog subfamily A member 3, mitochondrial  Transcription factor A, mitochondrial  Laminin subunit alpha-2  Amine oxidase [flavin-containing] B  Elongin-B  Methylglutaconyl-CoA hydratase, mitochondrial  Hydroxysteroid dehydrogenase-like protein 2  Glutathione S-transferase kappa 1  60S ribosomal protein L38  D-dopachrome decarboxylase  Sodium/calcium exchanger 1  Cysteine desulfurase, mitochondrial  Kynurenine--oxoglutarate transaminase 3  Fumarylacetoacetase | 6  5.67  5.52  5  4.67  4.67  4.67  4.36  4.33  3.7  3.67  3.54  3.53  3.47  3.45  3.36  3.33  3.33  3.26  3.23  3.2  3.13  3.11  3.04  2.99  2.95  2.95  2.90  2.89  2.86  2.84  2.83  2.78  2.76  2.72  2.62  2.6  2.5  2.47  2.42  2.4  2.37  2.36  2.36  2.34  2.33  2.32  2.31  2.29  2.25  2.25  2.25  2.24  2.24  2.24  2.22  2.22  2.19  2.19  2.18  2.17  2.15  2.15  2.15  2.12  2.12  2.12  2.12  2.1  2.09  2.09  2.08  2.07  2.04  2.01  1.98  1.96  1.95  1.93  1.9  1.86  1.86  1.86  1.85  1.82  1.81  1.79  1.77  1.74  1.71  1.71  1.68  1.68  1.65  1.62  1.61  1.61  1.6  1.6  1.6  1.58  1.58  1.57  1.55  1.55  1.52  1.51  1.5  1.5  1.48  1.47  1.44  1.43  1.41  1.41  1.38  1.38  1.37  1.37  1.36  1.35  1.35  1.35  1.34  1.34  1.33  1.3  1.25  1.25  0.88  0.86  0.85  0.85  0.84  0.83  0.82  0.81  0.8  0.8  0.79  0.79  0.76  0.76  0.73  0.7  0.69  0.69  0.68  0.68  0.67  0.67  0.66  0.65  0.65  0.65  0.64  0.63  0.63  0.62  0.62  0.62  0.61  0.6  0.6  0.56  0.53  0.52  0.51  0.5  0.47  0.47  0.47  0.46  0.46  0.46  0.46  0.46  0.46  0.45  0.45  0.42  0.41  0.38  0.38  0.38  0.35  0.33  0.32  0.3  0.29 | Organization of the actin cytoskeleton  Hydrolysis of acyl-CoAs to the free fatty acid and coenzyme A  Collagen biosynthetic process  Ubiquitin-dependent protein catabolic process  Regulation of actin filament polymerization  Maintenance of the myofibers cytoarchitecture/functional integrity  Cell redox homeostasis  Actin cytoskeleton organization  Regulation of actin filament bundle assembly  A role in tumorigenesis  Clathrin-dependent endocytosis  Nuclear envelope organization  Negative regulation of the canonical NF-kappa-B-activating branch  Positive regulation of nitric oxide biosynthetic process  Down-regulation of the early phase of the inflammatory response  Disulfide reduction activity/chaperone properties  Mediating the binding of neutrophils  Transport along microtubule  Negative regulation of protein tyrosine kinase activity  Chaperone-mediated protein folding  Hydroxylation of an Asp or Asn residue in certain epidermal growth factor-like domains  Endosomal transport  Cytoplasmic translation  Endothelial cell activation  Desaturation and elongation of fatty acids, cholesterol biosynthesis  Positive regulation of SMAD protein signal transduction  Anchoring of basement membranes to the underlying connective tissue  Protein folding  Membrane fusion/exocytosis  Regulation of myosin filament formation and sarcomere assembly  Protein localization to plasma membrane  Regulation of cell migration through association with the actin cytoskeleton  Actin cytoskeleton organization  Stabilization of actin structures/participation in actin-associated signalling pathways  Protection of actin filaments from depolymerization  Proteasome-mediated ubiquitin-dependent protein catabolic process  Actin cytoskeleton organization  Actin cytoskeleton organization  Blood coagulation/tissue remodelling  Connection of major cytoskeletal structures to the plasma membrane  Actin cytoskeleton organization  Glutamyl/prolyl-tRNA aminoacylation  Resealing of membranes disrupted by mechanical stress  Adenylate cyclase-modulating G protein-coupled receptor signalling  Microtubule cytoskeleton organization  Regulation of actin filament bundle assembly/actin crosslink formation  An electron carrier for several membrane-bound oxygenases  Cytoplasmic translation  Regulation of autophagy  Formation of a high-affinity link between the actin cytoskeleton and the membrane  Regulation of translational initiation  Mediation of the attachment, migration and organization of cells into tissues  Actin cytoskeleton reorganization  Regulation of cardiac muscle cell contraction  Protein transport/chromosome segregation  Muscle regeneration following muscle injury  Cell proliferation and survival  Cytoplasmic translation  Quality control of the ER by the retention of incorrectly folded proteins  Inhibition of actin polymerization  AP2-dependent clathrin-mediated endocytosis  Regulation of proliferation and differentiation of myoblasts  Cell chemotaxis/migration/cell population proliferation  Protein folding  Structural component of the microfibrils of the extracellular matrix  Proteolysis of substrates involved in cytoskeletal remodelling and signal transduction  Protein folding  Alternative mRNA splicing  Proteasome-mediated ubiquitin-dependent protein catabolic process  Chaperone-mediated protein folding  Glucose transporter recycling/movement of intracellular GLUT4-containing vesicles to the plasma membrane  Clathrin-dependent endocytosis  Triglyceride metabolic process  Protein folding  Cellular detoxification of nitrogen compound  Degradation of misfolded, unassembled or oxidatively damaged polypeptides  Connection of major cytoskeletal structures to the plasma membrane  Cell adhesion  Cytoplasmic translational initiation  mRNA processing  A structural component of connective tissues/fibrils formation  Canonical glycolysis  Cardiac muscle contraction  Cardiac remodelling in response to pressure overload  Lysosomal degradation of sphingolipids  Canonical glycolysis  Protein ubiquitination  Phosphocreatine biosynthetic process  Microtubule cytoskeleton organization  Adapter protein serving as a link between ABL kinases and actin cytoskeleton  Mediation in the linkage of cadherins to the actin cytoskeleton at adherens junctions  Actin cytoskeleton organization  Maintaining the structural integrity of the muscle fibers  Inhibition of extrinsic/intrinsic apoptotic pathways  3'-UTR-mediated mRNA destabilization  Pentose-phosphate shunt, oxidative branch  Iron transport across the cell membrane  Mitochondrial DNA catabolic process  Protein refolding  Formation of cytoplasmic translation initiation complex  Intermediate filament organization  Microtubule cytoskeleton organization  Actomyosin structure organization  Cell-cell adhesion  Nuclear envelope organization  Cell adhesion  Actin filament fragmentation  Internalization of GLUT4/membrane trafficking between the plasma membrane and endosomes  Cross-linking and stabilization of cytoskeletal intermediate filaments network  Microtubule cytoskeleton organization  Cytoskeleton organization/endocytosis  Proteasomal protein catabolic process  Chaperone-mediated protein folding  Negative regulation of apoptotic process  Mitochondrial electron transport, NADH to ubiquinone  Regulation of apoptosis/positive regulation of release of cytochrome c from mitochondria  Regulation of cell migration/adhesion  Actin cytoskeleton organization  Cell differentiation  Glycolysis/maintaining the integrity of the outer mitochondrial membrane  Detoxification of formaldehyde  Regulation of cardiomyocyte expansion/ promoting the development of hypertrophy  mRNA modification  Stabilization of the heart wall after cellular injury  Anchoring of actin to a variety of intracellular structures  Actin cytoskeleton organization  Synthesis of nucleoside triphosphates other than ATP  Actin cytoskeleton organization  Regulation of signal transduction  Lactate biosynthetic process from pyruvate  Glucose flux through polyol pathway  Fatty acid beta-oxidation  Exchange of sodium and potassium ions across the plasma membrane  Fatty acid beta-oxidation  Mitochondrial electron transport, NADH to ubiquinone  Fatty acid transport  Tricarboxylic acid cycle  Mitochondrial electron transport, ubiquinol to cytochrome c  Ketone body catabolic process  Ubiquinone biosynthetic process  Cell redox homeostasis  Fatty acid biosynthetic process  Fatty acid transport/beta-oxidation  ATP biosynthetic process  Mitochondrial electron transport, NADH to ubiquinone  Intra-Golgi protein transport  Xenobiotic metabolic process  Catabolism of odd chain fatty acids, branched-chain amino acids  Leucine catabolic process  Maintaining mitochondrial function and polarization/regulation of mitochondrial respiration  Short-/medium-chain fatty acid metabolic process  Regulation of intracellular levels of acyl-CoAs, free fatty acids and CoASH  Long-chain fatty acid catabolic process  Medium-chain fatty acid catabolic process  Fatty acid beta-oxidation  Long-chain and very long chain fatty acid catabolic process  Ubiquinone biosynthetic process  Transport across the plasma membrane of monocarboxylates (i.e. lactate, pyruvate, branched-chain oxo acids, ketone bodies)  Blood coagulation, fibrin clot formation  Valine catabolic process  Valine catabolic process  Formation and maintenance of lipid storage droplets  Regulation of mitochondrial respiration and mitochondrial DNA copy number maintenance  Calcium dependent regulation of striated muscle contraction  Cellular calcium ion homeostasis  Protein insertion into mitochondrial inner membrane  Transfer of electrons from NADH to the respiratory chain  Branched-chain amino acid catabolic process  Fatty acid transport/beta-oxidation  Protein de-ADP-ribosylation  ATP-dependent degradation of ubiquitinated proteins  ATP binding/hydrolysis  Cytoskeleton organization  Transport of acylcarnitines of different length across the mitochondrial inner membrane  Fatty acid metabolic process/activation  Mitochondrial proton-transporting ATP synthase complex assembly  Modulation of apoptotic signal transduction within the mitochondrial matrix  Mitochondrial transcription regulation/ maintenance of normal levels of mitochondrial DNA  Mediation in the attachment, migration and organization of cells into tissues  Metabolism of vasoactive amines  Transcription elongation by RNA polymerase II promoter  Leucine catabolic process/detoxification of itaconate  Short-chain oxidoreductase activity/regulation of lipid metabolism  Glutathione metabolic process  Regulation of translation  Positive regulation of inflammatory response/melanin biosynthetic process  Calcium ion homeostasis  Removal of elemental sulfur from cysteine to produce alanine  L-kynurenine catabolic process  L-phenylalanine/tyrosine catabolic process/ acetoacetate and fumarate formation |

190 statistically significant proteins out of 1134 included in the analysis (129 up-regulated, 61 down-regulated). Proteins related to metabolism are marked in purple. Note: some of the entities were slightly above/below FC threshold but were retained in the dataset due to biological importance.

**Sup. Table 5.** Differentially expressed proteins (DEPs) in the dataset: **12-month-old Tgαq*44 vs. age-matched control FVB mice** (fold change (Tgαq*44 /Control) ≥1.2 or ≤0.8 and adjusted *p* value <0.05); *n* = 6/group.

| No. | Accession  number | Annotation | FC (Tg/Ctrl) | Molecular Function |
| --- | --- | --- | --- | --- |
| 1  2  3  4  5  6  7  8  9  10  11  12  13  14  15  16  17  18  19  20  21  22  23  24  25  26  27  28  29  30  31  32  33  34  35  36  37  38  39  40  41  42  43  44  45  46  47  48  49  50  51  52  53  54  55  56  57  58  59  60  61  62  63  64  65  66  67  68  69  70  71  72  73  74  75  76  77  78  79  80  81  82  83  84  85  86  87  88  89  90  91  92  93  94  95  96  97  98  99  100  101  102  103  104  105  106  107  108  109  110  111  112  113  114  115  116  117  118  119  120  121  122  123  124  125  126  127  128  129  130  131  132  133  134  135  136  137  138  139  140  141  142  143  144  145  146  147  148  149  150  151  152  153  154  155  156  157  158  159  160  161  162  163  164  165  166  167  168  169  170  171  172  173  174  175  176  177  178  179  180  181  182  183  184  185  186  187  188  189  190  191  192  193  194  195  196  197  198  199  200  201  202  203  204  205  206  207  208  209  210  211  212  213  214  215  216  217  218  219  220  221  222  223  224  225  226  227  228  229  230  231  232  233  234  235  236  237  238 | Q4U4S6  Q62009  Q9CR42  Q80XB4  P28653  Q7TPW1  O35639  Q9R0P9  P24549  P62245  Q99P72  P10107  O35887  P24369  Q9WVA4  Q9DCN2  P35385  Q99MQ4  Q9EQK5  Q8BH61  A2AUC9  P35293  P19324  Q9DB73  P40142  Q9R0X4  Q8K4L3  P23506  O08547  Q62188  Q9ESD7  Q61554  P97447  P24452  Q61738  Q02788  Q8BWB1  Q04857  Q3UH68  Q9R062  P14602  Q9CQ19  O70373  P26041  P20152  Q60854  Q9EQP2  Q3URD3  P20918  O55131  P05125  P09103  P42208  Q61768  Q9CPV4  P49722  Q61543  Q9D1H6  P26039  Q61147  P17742  Q9WUA3  Q8VDD5  Q9R1P3  P09055  Q7M6Y3  Q91VI7  Q9Z204  O08553  Q9QZ06  Q6URW6  Q61937  Q9JK53  Q8CGK3  P27773  P13020  P53994  Q8BML9  P46935  Q6NZJ6  Q8BU30  Q64516  Q71LX4  Q68FD5  Q8BZF8  P08113  P63101  P62908  P16381  Q9JHU4  P28654  O08638  Q9DBG3  P07901  P48678  P11983  P51150  Q8C129  P14211  P07356  P48036  Q3U7R1  Q8BG05  Q7TMM9  Q9Z1N5  P63325  Q8BTM8  Q01853  Q8VEK3  P51885  P14869  Q64727  Q9D1X0  P24270  P60710  Q6PHZ2  P97384  Q9WVK4  Q9R0P3  P27546  Q9WVE8  Q04447  Q91W90  Q9D6Y9  Q9DBG6  P26231  P61161  O70400  Q921I1  Q91Z83  Q3UTJ2  O55222  Q9D6U8  P68372  P20029  P10126  Q61598  Q9QXS1  P31001  P17710  Q9QUI0  Q8VHX6  Q80X90  Q62261  P49312  P57780  P12382  P99027  P16546  P26043  Q7TPR4  E9Q557  Q62417  O08528  P67778  Q61941  Q7TQ48  Q99JY0  Q08857  P45376  Q91YT0  Q924X2  Q9D6J6  Q9WTP6  Q0II04  Q8QZT1  P04117  Q9CR21  Q8BMS1  P11404  O88844  Q921G7  P47934  Q8R1S0  Q9EQ20  P41216  Q9DCJ5  P97443  Q9JHI5  Q8VDN2  Q9CQ54  P49813  P17563  O35658  Q9CQQ7  Q8BFP9  P10649  Q8R086  Q9R112  Q9WUR2  Q8R0F8  Q9CQ62  Q8BGK2  P15626  Q8BWT1  P47911  P52825  Q99KQ4  Q4VAE3  Q9ET78  P09671  Q9ERS2  Q06185  Q99L13  Q3ULD5  P16332  Q9QYR9  Q99LP6  Q9CQJ8  O35459  Q9Z2I8  E9Q401  Q922B1  Q9DBG5  P50544  P23242  Q3TLP5  Q80XN0  Q9JLZ3  Q8CC88  O35855  A3KMP2  Q9Z1E4  O35943  Q8BW75  P09541  Q9D1I5  Q9Z1P6  Q9Z2Z6  O35215  Q9DCM2  P70414  Q64669  Q8BH86  Q2TPA8  P50136  Q8VCW8  Q71RI9 | Xin actin-binding repeat-containing protein 2  Periostin  Ankyrin repeat domain-containing protein 1  Nebulin-related-anchoring protein  Biglycan  Nexilin  Annexin A3  Ubiquitin carboxyl-terminal hydrolase isozyme L1  Aldehyde dehydrogenase 1A1  40S ribosomal protein S15a  Reticulon-4  Annexin A1  Calumenin  Peptidyl-prolyl cis-trans isomerase B  Transgelin-2  NADH-cytochrome b5 reductase 3  Heat shock protein beta-7  Asporin  Major vault protein  Coagulation factor XIII A chain  Kelch-like protein 41  Ras-related protein Rab-18  Serpin H1  NADH-cytochrome b5 reductase 1  Transketolase  Acyl-coenzyme A thioesterase 9, mitochondrial  Supervillin  Protein-L-isoaspartate (D-aspartate) O-methyltransferase  Vesicle-trafficking protein SEC22b  Dihydropyrimidinase-related protein 3  Dysferlin  Fibrillin-1  Four and a half LIM domains protein 1  Macrophage-capping protein  Integrin alpha-7  Collagen alpha-2(VI) chain  Synaptopodin 2-like protein  Collagen alpha-1(VI) chain  LIM and calponin homology domains-containing protein 1  Glycogenin-1  Heat shock protein beta-1  Myosin regulatory light polypeptide 9  Xin actin-binding repeat-containing protein 1  Moesin  Vimentin  Serpin B6  EH domain-containing protein 4  Sarcolemmal membrane-associated protein  Plasminogen  Septin-7  Natriuretic peptides A  Protein disulfide-isomerase (PDIA1)  Septin-2  Kinesin-1 heavy chain  Glyoxalase domain-containing protein 4  Proteasome subunit alpha type-2  Golgi apparatus protein 1  NADH dehydrogenase [ubiquinone] 1 alpha subcomplex assembly factor 4  Talin-1  Ceruloplasmin  Peptidyl-prolyl cis-trans isomerase A  ATP-dependent 6-phosphofructokinase, platelet type  Myosin-9  Proteasome subunit beta type-2  Integrin beta-1  Phosphatidylinositol-binding clathrin assembly protein  Ribonuclease inhibitor  Heterogeneous nuclear ribonucleoproteins C1/C2  Dihydropyrimidinase-related protein 2  Toll-interacting protein  Myosin-14  Nucleophosmin  Prolargin  Lon protease homolog, mitochondrial  Protein disulfide-isomerase A3 (PDIA3)  Gelsolin  Ras-related protein Rab-2A  Glutamine--tRNA ligase  E3 ubiquitin-protein ligase NEDD4  Eukaryotic translation initiation factor 4 gamma 1  Isoleucine--tRNA ligase, cytoplasmic  Glycerol kinase  Talin-2  Clathrin heavy chain 1  Phosphoglucomutase-like protein 5  Endoplasmin  14-3-3 protein zeta/delta  40S ribosomal protein S3  Putative ATP-dependent RNA helicase Pl10  Cytoplasmic dynein 1 heavy chain 1  Decorin  Myosin-11  AP-2 complex subunit beta  Heat shock protein HSP 90-alpha  Prelamin-A/C  T-complex protein 1 subunit alpha  Ras-related protein Rab-7a  Leucyl-cystinyl aminopeptidase  Calreticulin  Annexin A2  Annexin A5  Extended synaptotagmin-1  Heterogeneous nuclear ribonucleoprotein A3  Tubulin beta-2A chain  Spliceosome RNA helicase Ddx39b  40S ribosomal protein S10  Filamin-A  Transitional endoplasmic reticulum ATPase  Heterogeneous nuclear ribonucleoprotein U  Lumican  60S acidic ribosomal protein P0  Vinculin  Nucleolar protein 3  Catalase  Actin, cytoplasmic 1  Calcium/calmodulin-dependent protein kinase type II subunit delta  Annexin A11  EH domain-containing protein 1  S-formylglutathione hydrolase  Microtubule-associated protein 4  Protein kinase C and casein kinase substrate in neurons protein 2  Creatine kinase B-type  Thioredoxin domain-containing protein 5  1,4-alpha-glucan-branching enzyme  Dolichyl-diphosphooligosaccharide--protein glycosyltransferase subunit 2  Catenin alpha-1  Actin-related protein 2  PDZ and LIM domain protein 1  Serotransferrin  Myosin-7  Sorbin and SH3 domain-containing protein 2  Integrin-linked protein kinase  Protein FAM162A  Tubulin beta-4B chain  Endoplasmic reticulum chaperone BiP  Elongation factor 1-alpha 1  Rab GDP dissociation inhibitor beta  Plectin  Desmin  Hexokinase-1  Transforming protein RhoA  Filamin-C  Filamin-B  Spectrin beta chain, non-erythrocytic 1  Heterogeneous nuclear ribonucleoprotein A1  Alpha-actinin-4  ATP-dependent 6-phosphofructokinase, liver type  60S acidic ribosomal protein P2  Spectrin alpha chain, non-erythrocytic 1  Radixin  Alpha-actinin-1  Desmoplakin  Sorbin and SH3 domain-containing protein 1  Hexokinase-2  Prohibitin 1  NAD(P) transhydrogenase, mitochondrial  Sarcalumenin  Trifunctional enzyme subunit beta, mitochondrial  Platelet glycoprotein 4  Aldo-keto reductase family 1 member B1  NADH dehydrogenase [ubiquinone] flavoprotein 1, mitochondrial  Carnitine O-palmitoyltransferase 1, muscle isoform  NADH dehydrogenase [ubiquinone] flavoprotein 2, mitochondrial  Adenylate kinase 2, mitochondrial  Nebulette  Acetyl-CoA acetyltransferase, mitochondrial  Fatty acid-binding protein, adipocyte  Acyl carrier protein, mitochondrial  Trifunctional enzyme subunit alpha, mitochondrial  Fatty acid-binding protein, heart  Isocitrate dehydrogenase [NADP] cytoplasmic  Electron transfer flavoprotein-ubiquinone oxidoreductase, mitochondrial  Carnitine O-acetyltransferase  Ubiquinone biosynthesis monooxygenase COQ6, mitochondrial  Methylmalonate-semialdehyde dehydrogenase [acylating], mitochondrial  Long-chain-fatty-acid--CoA ligase 1  NADH dehydrogenase [ubiquinone] 1 alpha subcomplex subunit 8  Histone-lysine N-methyltransferase Smyd1  Isovaleryl-CoA dehydrogenase, mitochondrial  Sodium/potassium-transporting ATPase subunit alpha-1  NADH dehydrogenase [ubiquinone] 1 subunit C2  Tropomodulin-1  Methanethiol oxidase  Complement component 1 Q subcomponent-binding protein, mitochondrial  ATP synthase F(0) complex subunit B1, mitochondrial  [Pyruvate dehydrogenase (acetyl-transferring)] kinase isozyme 1, mitochondrial  Glutathione S-transferase Mu 1  Sulfide oxidase, mitochondrial  Sulfide:quinone oxidoreductase, mitochondrial  Enoyl-CoA delta isomerase 2  Acylpyruvase FAHD1, mitochondrial  2,4-dienoyl-CoA reductase [(3E)-enoyl-CoA-producing], mitochondrial  Inactive ADP-ribosyltransferase ARH2  Glutathione S-transferase Mu 2  3-ketoacyl-CoA thiolase, mitochondrial  60S ribosomal protein L6  Carnitine O-palmitoyltransferase 2, mitochondrial  Nicotinamide phosphoribosyltransferase  Transmembrane protein 65  Junctophilin-2  Superoxide dismutase [Mn], mitochondrial  NADH dehydrogenase [ubiquinone] 1 alpha subcomplex subunit 13  ATP synthase subunit e, mitochondrial  3-hydroxyisobutyrate dehydrogenase, mitochondrial  Methylcrotonoyl-CoA carboxylase beta chain, mitochondrial  Methylmalonyl-CoA mutase, mitochondrial  Acyl-coenzyme A thioesterase 2, mitochondrial  GrpE protein homolog 1, mitochondrial  NADH dehydrogenase [ubiquinone] 1 beta subcomplex subunit 9  Delta(3,5)-Delta(2,4)-dienoyl-CoA isomerase, mitochondrial  Succinate--CoA ligase [GDP-forming] subunit beta, mitochondrial  Ryanodine receptor 2  ADP-ribose glycohydrolase MACROD1  Perilipin-3  Very long-chain specific acyl-CoA dehydrogenase, mitochondrial  Gap junction alpha-1 protein  Enoyl-CoA hydratase domain-containing protein 2, mitochondrial  D-beta-hydroxybutyrate dehydrogenase, mitochondrial  Methylglutaconyl-CoA hydratase, mitochondrial  von Willebrand factor A domain-containing protein 8  Branched-chain-amino-acid aminotransferase, mitochondrial  Tetratricopeptide repeat protein 38  Glycogen [starch] synthase, muscle  Frataxin, mitochondrial  Amine oxidase [flavin-containing] B  Myosin light chain 4  Methylmalonyl-CoA epimerase, mitochondrial  NADH dehydrogenase [ubiquinone] 1 alpha subcomplex subunit 7  Mitochondrial carnitine/acylcarnitine carrier protein  D-dopachrome decarboxylase  Glutathione S-transferase kappa 1  Sodium/calcium exchanger 1  NAD(P)H dehydrogenase [quinone] 1  D-glutamate cyclase, mitochondrial  Hydroxysteroid dehydrogenase-like protein 2  2-oxoisovalerate dehydrogenase subunit alpha, mitochondrial  Medium-chain acyl-CoA ligase ACSF2, mitochondrial  Kynurenine--oxoglutarate transaminase 3 | 13  10  8.33  6.5  5.91  5.67  5.29  5.22  5.19  5.17  5  4.91  4.75  4.62  4.59  4.57  4.36  4.1  3.93  3.8  3.79  3.55  3.4  3.38  3.33  3.33  3.25  3.07  2.94  2.89  2.89  2.87  2.86  2.82  2.8  2.8  2.77  2.76  2.75  2.74  2.72  2.52  2.45  2.44  2.42  2.41  2.38  2.37  2.33  2.32  2.32  2.31  2.27  2.26  2.26  2.23  2.22  2.22  2.21  2.21  2.2  2.2  2.2  2.2  2.17  2.17  2.17  2.15  2.14  2.13  2.12  2.07  2.06  2.06  2.02  2.02  2.01  2  2  2  2  1.98  1.97  1.97  1.95  1.92  1.91  1.89  1.87  1.87  1.86  1.86  1.86  1.85  1.85  1.83  1.83  1.82  1.78  1.78  1.76  1.75  1.73  1.73  1.71  1.71  1.71  1.71  1.68  1.67  1.67  1.66  1.65  1.64  1.62  1.6  1.6  1.59  1.58  1.57  1.56  1.55  1.55  1.54  1.54  1.54  1.52  1.52  1.52  1.5  1.44  1.43  1.43  1.42  1.42  1.41  1.41  1.41  1.4  1.4  1.39  1.39  1.37  1.35  1.35  1.34  1.34  1.32  1.31  1.31  1.28  1.27  1.25  1.22  0.81  0.8  0.8  0.8  0.79  0.79  0.78  0.78  0.77  0.76  0.76  0.76  0.75  0.75  0.74  0.74  0.74  0.73  0.73  0.71  0.71  0.7  0.7  0.69  0.69  0.69  0.68  0.68  0.68  0.67  0.67  0.67  0.65  0.65  0.64  0.64  0.63  0.63  0.63  0.62  0.62  0.62  0.62  0.6  0.6  0.6  0.6  0.59  0.59  0.57  0.56  0.56  0.56  0.55  0.54  0.54  0.53  0.52  0.52  0.52  0.52  0.52  0.52  0.51  0.49  0.49  0.48  0.46  0.45  0.45  0.45  0.4  0.39  0.38  0.38  0.35  0.35  0.33  0.32  0.28  0.23  0.22  0.21  0.13 | Protecting actin filaments from depolymerization  Induction of cell attachment and spreading  Endothelial cell activation  Actin cytoskeleton organization  Involved in collagen fiber assembly  Regulation of cell migration through association with the actin cytoskeleton  Regulation of cellular growth/signal transduction pathways/anti-coagulant properties  Processing of ubiquitin precursors and ubiquitinated proteins  Cellular detoxification of aldehydes  Cytoplasmic translation  Endoplasmic reticulum tubular network organization  Glucocorticoid-mediated down-regulation of the early phase of the inflammatory response  Calcium-binding protein localized in the ER involved in protein folding and sorting  Protein folding  Epithelial cell differentiation  Cholesterol biosynthetic process  Heart development/regulation of heart contraction  Calcium-binding protein playing a role in osteoblast-driven collagen biomineralization activity  Protein activation cascade  Blood coagulation/fibrin clot formation  Regulation of proliferation and differentiation of myoblasts  Transport between the plasma membrane and early endosomes  Collagen biosynthetic process  Desaturation and elongation of fatty acids, cholesterol biosynthesis  Pentose-phosphate shunt, non-oxidative branch  Regulation of intracellular levels of acyl-CoAs, free fatty acids and CoASH  Formation of a high-affinity link between the actin cytoskeleton and the membrane  Repair and/or degradation of damaged proteins  Endoplasmic reticulum to Golgi vesicle-mediated transport  Actin crosslink formation  A role in the sarcolemma repair mechanism of both skeletal muscle and cardiomyocytes  Structural component of microfibrils of the extracellular matrix  Cell differentiation  Barbed-end actin filament capping  Maintenance of the myofibers cytoarchitecture/regulation of its anchoring, viability and functional integrity  A cell-binding protein  Actin-associated protein playing a role in modulating actin-based shape  A cell-binding protein  Actomyosin structure organization  Glycogen biosynthetic process  Chaperone-mediated protein folding  Regulation of both smooth muscle and non-muscle cell contractile activity via its phosphorylation  Protecting actin filaments from depolymerization  Connection of major cytoskeletal structures to the plasma membrane  Intermediate filament organization  Regulation of endopeptidase activity, e.g. cathepsin G, kallikrein-8 and thrombin  Controlling membrane reorganization/ tubulation upon ATP hydrolysis/endocytic recycling  A role during myoblast fusion  Blood coagulation  Skeletal muscle architecture and function maintaining  Regulation of natriuresis, diuresis, and vasodilation  Protein folding  Cytoskeleton-dependent cytokinesis  Transport of cargo along microtubules  Cadherin binding activity  Ubiquitin-dependent protein catabolic process  Mediating in the binding of neutrophils  Negative regulation of apoptotic process  Connection of major cytoskeletal structures to the plasma membrane  Iron transport across the cell membrane  Protein folding  Canonical glycolysis  Actin cytoskeleton reorganization  ATP-dependent degradation of ubiquitinated proteins  Cell adhesion  AP2-dependent clathrin-mediated endocytosis at the neuromuscular junction  Regulation of redox homeostasis  3'-UTR-mediated mRNA stabilization  Cytoskeleton organization/endocytosis  Autophagic clearance of ubiquitin conjugates  Cytokinesis/Regulation of cell shape  Ribosome biogenesis/centrosome duplication/protein chaperoning  Anchoring of basement membranes to the underlying connective tissue Degradation of misfolded, unassembled or oxidatively damaged polypeptides in the mitochondrial matrix  Protein folding  Actin filament capping  Vesicle-mediated protein transport  Glutaminyl-tRNA aminoacylation  Protein ubiquitination  Translational initiation  Isoleucyl-tRNA aminoacylation  Glycerol-3-phosphate biosynthetic process/glycolysis  Cell-cell adhesion  Early autophagosome formation  Component of adherens-type cell-cell and cell-matrix junctions  Processing and transport of secreted proteins  Regulation of signal transduction  Cytoplasmic translation/DNA repair  Cell differentiation  Regulation of intracellular retrograde motility of vesicles and organelles along microtubules  Regulation of the rate of fibrils formation  Actomyosin structure organization  Clathrin-dependent endocytosis  Chaperone-mediated protein complex assembly  Nuclear envelope organization  Chaperone-mediated protein folding  Intracellular protein transport  Peptide catabolic process  Promoting folding, oligomeric assembly and quality control in the endoplasmic reticulum  Regulation of exocytosis  Negative regulation of blood coagulation  Intermembrane lipid transfer  Cytoplasmic trafficking of RNA/mRNA splicing  Microtubule cytoskeleton organization  Nuclear export of spliced and unspliced mRNA  Cytoplasmic translation  Anchoring of various transmembrane proteins to the actin cytoskeleton  Fragmentation of Golgi stacks during mitosis/their reassembly after mitosis  Nuclear chromatin organization/telomere-length regulation/transcription/mRNA alternative splicing and stability  Regulation of collagen fibril structure  Cytoplasmic translation  Regulation of protein localization to adherens junction  Negative regulation of apoptotic process  Protecting cells from the toxic effects of hydrogen peroxide  An essential part of the contractile apparatus in muscle  Regulation of release of sequestered calcium ion into cytosol by sarcoplasmic reticulum  Cytokinesis  Membrane reorganization/tubulation upon ATP hydrolysis  Detoxification of formaldehyde  Microtubule cytoskeleton organization  Intracellular vesicle-mediated transport  Phosphocreatine biosynthetic process  Protein folding  Glycogen accumulation  Protein N-linked glycosylation  Mediating in the linkage of cadherins to the actin cytoskeleton at adherens junctions  Actin cytoskeleton organization  Establishment or maintenance of actin cytoskeleton polarity  Regulation of iron ion transport  Cardiac muscle contraction  Assembling of signalling complexes  Regulation of integrin-mediated signal transduction  Regulation of apoptosis  Microtubule cytoskeleton organization  Folding of proteins and degradation of misfolded proteins  Translational elongation  Vesicle-mediated protein transport  Cross-linking and stabilization of cytoskeletal intermediate filaments network/regulation of its dynamics  Intermediate filament organization  Canonical glycolysis  Regulation of a signal transduction  Maintaining the structural integrity of the muscle fibers  Anchoring of various transmembrane proteins to the actin cytoskeleton  Calcium-dependent movement of the cytoskeleton at the membrane  mRNA splicing/mRNA transport  Anchoring of actin to a variety of intracellular structures  Canonical glycolysis  Cytoplasmic translational elongation  Calcium-dependent movement of the cytoskeleton at the membrane  Binding of the barbed end of actin filaments to the plasma membrane  Anchoring of actin to a variety of intracellular structures  Anchoring of intermediate filaments to the desmosomes  Positive regulation of glucose import  Glycolysis/negative regulation of mitochondrial membrane permeability  Mitochondrion organization  Cell redox homeostasis  Regulation of calcium transport  Fatty acid beta-oxidation  Cell surface receptor signalling/long-chain fatty acid import across plasma membrane  Cellular response to hydrogen peroxide  Mitochondrial electron transport, NADH to ubiquinone  Long-chain fatty acid transport  Mitochondrial electron transport, NADH to ubiquinone  Adenine nucleotide metabolism/AMP de novo biosynthesis pathway  Cardiac muscle thin filament assembly  Ketone body metabolism  Lipid transport  Transfer of electrons from NADH to the respiratory chain  Fatty acid beta-oxidation  Intracellular transport of long-chain fatty acids and their acyl-CoA esters  Generation of NADPH  Accepting of electrons from ETF and reducing of ubiquinone  Transport of acetyl-CoA into mitochondria  Ubiquinone biosynthetic process  Valine catabolic process  Activation of long-chain fatty acids for both synthesis of cellular lipids, and degradation via beta-oxidation  Mitochondrial electron transport, NADH to ubiquinone  Regulation of myoblast differentiation  Leucine catabolic process  Exchange of sodium and potassium ions across the plasma membrane  Mitochondrial electron transport, NADH to ubiquinone  Blocking of the elongation and depolymerization of the actin filaments  Intra-Golgi protein transport  Ribosome biogenesis/protein synthesis in mitochondria/regulation of apoptosis  Proton motive force-driven mitochondrial ATP synthesis  Inhibition of pyruvate dehydrogenase activity  Cellular detoxification of nitrogen compound  Sulfur compound metabolic process  Hydrogen sulfide metabolic process  Fatty acid beta-oxidation  Hydrolysing of acetylpyruvate and fumarylpyruvate  Fatty acid beta-oxidation  Protein de-ADP-ribosylation  Cellular detoxification of nitrogen compound  Fatty acid beta-oxidation/Abolishing of BNIP3-mediated apoptosis and mitochondrial damage  Cytoplasmic translation  Long-chain fatty acid transport/beta-oxidation  NAD biosynthesis  Regulation of mitochondrial respiration and mitochondrial DNA copy number maintenance  Regulation of ryanodine-sensitive calcium-release channel activity  Removal of superoxide anion radicals  Transfer of electrons from NADH to the respiratory chain  Proton motive force-driven mitochondrial ATP synthesis  Valine catabolic process  Leucine catabolic process  Branched-chain amino acid catabolism/succinyl-CoA biosynthesis  Hydrolysis of acyl-CoAs to the free fatty acid and coenzyme A (CoASH)  Protein import into mitochondrial matrix  Mitochondrial electron transport, NADH to ubiquinone  Fatty acid beta-oxidation  Tricarboxylic acid cycle  Calcium channel that mediates the release of Ca^(2+)^ from the sarcoplasmic reticulum into the cytoplasm  Protein de-ADP-ribosylation  Lipid storage/regulation of sequestering of triglycerides  Fatty acid beta-oxidation  Cell communication  Fatty acid beta-oxidation  Ketone body metabolism  Leucine catabolic process/Detoxification of itaconate − a vitamin B12-poisoning metabolite  ATPase activity  Branched-chain amino acid catabolic process  Mediating in protein-protein interactions  Glycogen biosynthetic process  Protection against iron-catalyzed oxidative stress  Oxidative deamination of biogenic and xenobiotic amines  Regulation of the force of heart contraction  Degradation of branched amino acids and odd-chain fatty acids/conversion of propionyl-CoA to succinyl-CoA  Mitochondrial electron transport, NADH to ubiquinone  Transport of acylcarnitines across the mitochondrial inner membrane  Regulation of inflammatory response  Cellular detoxification  Regulation of cytoplasmic Ca^(2+)^ levels and Ca^(2+)-^dependent cellular processes  Cell redox homeostasis  Conversion of D-glutamate to 5-oxo- D-proline  Lipid metabolism regulator  Branched-chain amino acid catabolic process  Fatty acid metabolic process  L-kynurenine catabolic process |

238 statistically significant proteins out of 1213 included in the analysis (154 up-regulated, 84 down-regulated). Proteins related to metabolism are marked in purple. Note: some of the entities were slightly above/below FC threshold but were retained in the dataset due to biological importance.

**Sup. Table 6.** Differentially expressed proteins (DEPs) in the dataset: **14-month-old Tgαq*44 vs. age-matched control FVB mice** (fold change (Tgαq*44 /Control) ≥1.2 or ≤0.8 and adjusted *p* value <0.05); *n* = 6/group.

| No. | Accession  number | Annotation | FC (Tg/Ctrl) | Molecular Function |
| --- | --- | --- | --- | --- |
| 1  2  3  4  5  6  7  8  9  10  11  12  13  14  15  16  17  18  19  20  21  22  23  24  25  26  27  28  29  30  31  32  33  34  35  36  37  38  39  40  41  42  43  44  45  46  47  48  49  50  51  52  53  54  55  56  57  58  59  60  61  62  63  64  65  66  67  68  69  70  71  72  73  74  75  76  77  78  79  80  81  82  83  84  85  86  87  88  89  90  91  92  93  94  95  96  97  98  99  100  101  102  103  104  105  106  107  108  109  110  111  112  113  114  115  116  117  118  119  120  121  122  123  124  125  126  127  128  129  130  131  132  133  134  135  136  137  138  139  140  141  142  143  144  145  146  147  148  149  150  151  152  153  154  155  156  157  158  159  160  161  162  163  164  165  166  167  168  169  170  171  172  173  174  175  176  177  178  179  180  181  182  183  184  185  186  187  188  189  190  191  192  193  194  195  196  197  198  199  200  201  202  203  204  205  206  207  208  209  210  211  212  213  214  215  216  217  218  219  220  221  222  223  224  225  226  227  228  229  230  231  232  233  234  235  236  237  238  239  240  241  242  243  244  245  246  247  248  249  250  251  252  253  254  255  256  257  258  259  260  261  262  263  264  265  266  267  268  269  270  271  272  273  274  275 | Q80XB4  Q99MQ4  Q8BMK4  Q9CR42  Q9WVA4  P28653  P24549  O35639  Q9DCN2  Q9R0X4  Q61292  Q9R0P9  P14685  Q7TPW1  Q9EQK5  P24452  P26041  Q62465  Q60854  Q62048  P97447  Q9JMC3  Q9EQP2  P20918  P37804  Q8BWB1  Q6NZJ6  P60766  Q9DB73  P24369  P14602  Q91YQ5  O70373  Q8K4L3  O55131  Q9JK53  P62334  P01027  P46935  Q3UH68  Q91VI7  Q6PHZ2  Q9ESD7  P19324  P62192  O55222  P21614  P35385  P80314  Q61147  P62245  Q8BG32  Q9WUA3  P51150  Q7M6Y3  P27773  Q8BTM8  Q921I1  Q9R0P5  A2AUC9  Q922R8  P20152  Q61554  O35206  P14211  P08113  Q3URD3  P02301  P09103  Q91Z53  Q6URW6  Q8VDD5  P97493  Q8R5J9  Q60590  O88456  Q9R1P0  Q811U4  Q02788  Q9D892  Q9CQE8  Q8R1G2  P80315  Q11011  Q8BGD9  P29341  P19221  P11276  P40124  Q76MZ3  Q8BZF8  O55234  Q61553  Q7TMM9  P46471  Q68FD5  P51885  P23506  Q07076  P07356  P27546  Q9JHU4  Q8VIJ6  Q99PT1  P51125  P42932  P08752  P42208  Q3UIZ8  Q9CYT6  Q99KJ8  Q8CGK3  Q01853  Q3UTJ2  P31001  P17710  Q80X90  O08553  P48678  Q99PR8  P61205  Q91Z83  P06151  Q60605  P07759  Q9DBJ1  P14206  Q9R062  O70400  Q9Z2U1  P17742  P26039  Q9QUI0  Q91X72  P99024  P10126  Q8CIB5  Q9WV55  Q8VHX6  Q04857  P05977  P80318  Q9DCL9  Q7TPR4  P14869  P68372  P28271  Q8CC35  P48036  O88685  P58252  Q02053  O08528  Q71LX4  P60710  P57780  Q8CI51  P28654  O08638  P09405  P06745  P20029  P07901  O88342  P10649  Q8BG05  P80313  E9Q557  Q9D6U8  P68369  Q9R0P3  P50247  Q6ZQ38  P45591  P09411  Q9QXS1  Q64727  P11499  Q9JJZ2  Q99NB1  Q60936  Q9Z0X1  Q9WUR2  P07310  P34914  P45376  Q9D8B4  P97807  Q61941  Q9Z2I8  Q99MN9  Q9JJW5  Q99J99  P51174  P47934  P41216  Q9Z2I9  Q924X2  Q7TMF3  P45952  Q91ZA3  Q8BMS1  Q8K4G5  Q8VDN2  P56375  P17563  Q3ULJ0  Q9D6J6  A3KMP2  P11152  Q921G7  P97443  Q9D7J9  Q8BGK2  Q3ULD5  O35855  Q4VAE3  Q9D6J5  Q8BWT1  Q8QZT1  P14094  Q80Y14  Q8BLF1  Q8CC88  Q99JY0  O35857  Q9CQJ8  Q9JHI5  P16332  Q9DCV4  Q99KQ4  Q924D0  Q9EQ20  P50136  Q9CQ62  P70414  Q65CL1  Q07417  P52825  P22315  E9Q401  O35459  Q9JKL4  Q3TLP5  Q8BWF0  P47802  Q60766  P50544  Q9Z2Z6  Q9D023  P62270  Q922B1  P63030  Q9JIF9  P62075  O70622  Q8BW75  Q9WVL0  Q9D6Y7  Q9D1I5  P85094  Q80XN0  Q9QYR9  Q3TC72  P53395  Q2TPA8  P01897  Q8C0M9  Q8BH86  P23242  Q8R1S0  Q9DCM2  Q9JLZ3  Q71RI9  Q9CRB8 | Nebulin-related-anchoring protein  Asporin  Cytoskeleton-associated protein 4  Ankyrin repeat domain-containing protein 1  Transgelin-2  Biglycan  Aldehyde dehydrogenase 1A1  Annexin A3  NADH-cytochrome b5 reductase 3  Acyl-coenzyme A thioesterase 9, mitochondrial  Laminin subunit beta-2  Ubiquitin carboxyl-terminal hydrolase isozyme L1  26S proteasome non-ATPase regulatory subunit 3  Nexilin  Major vault protein  Macrophage-capping protein  Moesin  Synaptic vesicle membrane protein VAT-1 homolog  Serpin B6  Astrocytic phosphoprotein PEA-15  Four and a half LIM domains protein 1  DnaJ homolog subfamily A member 4  EH domain-containing protein 4  Plasminogen  Transgelin  Synaptopodin 2-like protein  Eukaryotic translation initiation factor 4 gamma 1  Cell division control protein 42 homolog  NADH-cytochrome b5 reductase 1  Peptidyl-prolyl cis-trans isomerase B  Heat shock protein beta-1  Dolichyl-diphosphooligosaccharide--protein glycosyltransferase subunit 1  Xin actin-binding repeat-containing protein 1  Supervillin  Septin-7  Prolargin  26S proteasome regulatory subunit 10B  Complement C3  E3 ubiquitin-protein ligase NEDD4  LIM and calponin homology domains-containing protein 1  Ribonuclease inhibitor  Calcium/calmodulin-dependent protein kinase type II subunit delta  Dysferlin  Serpin H1  26S proteasome regulatory subunit 4  Integrin-linked protein kinase  Vitamin D-binding protein  Heat shock protein beta-7  T-complex protein 1 subunit beta  Ceruloplasmin  40S ribosomal protein S15a  26S proteasome non-ATPase regulatory subunit 11  ATP-dependent 6-phosphofructokinase, platelet type  Ras-related protein Rab-7a  Phosphatidylinositol-binding clathrin assembly protein  Protein disulfide-isomerase A3 (PDIA3)  Filamin-A  Serotransferrin  Destrin  Kelch-like protein 41  Protein disulfide-isomerase A6 (PDIA6)  Vimentin  Fibrillin-1  Collagen alpha-1(XV) chain  Calreticulin  Endoplasmin  Sarcolemmal membrane-associated protein  Histone H3.3C  Protein disulfide-isomerase (PDIA1)  Glyoxylate reductase/hydroxypyruvate reductase  Myosin-14  Myosin-9  Thioredoxin, mitochondrial  PRA1 family protein 3  Alpha-1-acid glycoprotein 1  Calpain small subunit 1  Proteasome subunit alpha type-4  Mitofusin-1  Collagen alpha-2(VI) chain  Inosine triphosphate pyrophosphatase  RNA transcription, translation and transport factor protein  Carboxymethylenebutenolidase homolog  T-complex protein 1 subunit delta  Puromycin-sensitive aminopeptidase  Eukaryotic translation initiation factor 4B  Polyadenylate-binding protein 1  Prothrombin  Fibronectin  Adenylyl cyclase-associated protein 1  Serine/threonine-protein phosphatase 2A 65 kDa regulatory subunit A alpha isoform  Phosphoglucomutase-like protein 5  Proteasome subunit beta type-5  Fascin  Tubulin beta-2A chain  26S proteasome regulatory subunit 7  Clathrin heavy chain 1  Lumican  Protein-L-isoaspartate(D-aspartate) O-methyltransferase  Annexin A7  Annexin A2  Microtubule-associated protein 4  Cytoplasmic dynein 1 heavy chain 1  Splicing factor, proline- and glutamine-rich  Rho GDP-dissociation inhibitor 1  Calpastatin  T-complex protein 1 subunit theta  Guanine nucleotide-binding protein G(i) subunit alpha-2  Septin-2  Myosin light chain kinase 3  Adenylyl cyclase-associated protein 2  Dynactin subunit 2  Lon protease homolog, mitochondrial  Transitional endoplasmic reticulum ATPase  Sorbin and SH3 domain-containing protein 2  Desmin  Hexokinase-1  Filamin-B  Dihydropyrimidinase-related protein 2  Prelamin-A/C  Heat shock protein beta-2  ADP-ribosylation factor 3  Myosin-7  L-lactate dehydrogenase A chain  Myosin light polypeptide 6  Serine protease inhibitor A3K  Phosphoglycerate mutase 1  40S ribosomal protein SA  Glycogenin-1  PDZ and LIM domain protein 1  Proteasome subunit alpha type-5  Peptidyl-prolyl cis-trans isomerase A  Talin-1  Transforming protein RhoA  Hemopexin  Tubulin beta-5 chain  Elongation factor 1-alpha 1  Fermitin family homolog 2  Vesicle-associated membrane protein-associated protein A  Filamin-C  Collagen alpha-1(VI) chain  Myosin light chain 1/3, skeletal muscle isoform  T-complex protein 1 subunit gamma  Bifunctional phosphoribosylaminoimidazole carboxylase/phosphoribosylaminoimidazole succinocarboxamide synthetase  Alpha-actinin-1  60S acidic ribosomal protein P0  Tubulin beta-4B chain  Cytoplasmic aconitate hydratase  Synaptopodin  Annexin A5  26S proteasome regulatory subunit 6A  Elongation factor 2  Ubiquitin-like modifier-activating enzyme 1  Hexokinase-2  Talin-2  Actin, cytoplasmic 1  Alpha-actinin-4  PDZ and LIM domain protein 5  Decorin  Myosin-11  Nucleolin  Glucose-6-phosphate isomerase  Endoplasmic reticulum chaperone BiP  Heat shock protein HSP 90-alpha  WD repeat-containing protein 1  Glutathione S-transferase Mu 1  Heterogeneous nuclear ribonucleoprotein A3  T-complex protein 1 subunit eta  Desmoplakin  Protein FAM162A  Tubulin alpha-1A chain  S-formylglutathione hydrolase  Adenosylhomocysteinase  Cullin-associated NEDD8-dissociated protein 1  Cofilin-2  Phosphoglycerate kinase 1  Plectin  Vinculin  Heat shock protein HSP 90-beta  Tubulin alpha-8 chain  Acetyl-coenzyme A synthetase 2-like, mitochondrial  Atypical kinase COQ8A, mitochondrial  Apoptosis-inducing factor 1, mitochondrial  Enoyl-CoA delta isomerase 2  Creatine kinase M-type  Bifunctional epoxide hydrolase 2  Aldo-keto reductase family 1 member B1  NADH dehydrogenase [ubiquinone] 1 alpha subcomplex subunit 11  Fumarate hydratase, mitochondrial  NAD(P) transhydrogenase, mitochondrial  Succinate--CoA ligase [GDP-forming] subunit beta, mitochondrial  Propionyl-CoA carboxylase beta chain, mitochondrial  Myozenin-2  3-mercaptopyruvate sulfurtransferase  Long-chain specific acyl-CoA dehydrogenase, mitochondrial  Carnitine O-acetyltransferase  Long-chain-fatty-acid--CoA ligase 1  Succinate--CoA ligase [ADP-forming] subunit beta, mitochondrial  Carnitine O-palmitoyltransferase 1, muscle isoform  NADH dehydrogenase [ubiquinone] 1 alpha subcomplex subunit 12  Medium-chain specific acyl-CoA dehydrogenase, mitochondrial  Propionyl-CoA carboxylase alpha chain, mitochondrial  Trifunctional enzyme subunit alpha, mitochondrial  Actin-binding LIM protein 1  Sodium/potassium-transporting ATPase subunit alpha-1  Acylphosphatase-2  Methanethiol oxidase  Glycerol-3-phosphate dehydrogenase 1-like protein  NADH dehydrogenase [ubiquinone] flavoprotein 2, mitochondrial  Tetratricopeptide repeat protein 38  Lipoprotein lipase  Electron transfer flavoprotein-ubiquinone oxidoreductase, mitochondrial  Histone-lysine N-methyltransferase Smyd1  Enoyl-CoA hydratase domain-containing protein 3, mitochondrial  Inactive ADP-ribosyltransferase ARH2  Methylcrotonoyl-CoA carboxylase beta chain, mitochondrial  Branched-chain-amino-acid aminotransferase, mitochondrial  Transmembrane protein 65  NADH dehydrogenase [ubiquinone] 1 beta subcomplex subunit 8, mitochondrial  3-ketoacyl-CoA thiolase, mitochondrial  Acetyl-CoA acetyltransferase, mitochondrial  Sodium/potassium-transporting ATPase subunit beta-1  Glutaredoxin-related protein 5, mitochondrial  Neutral cholesterol ester hydrolase 1  von Willebrand factor A domain-containing protein 8  Trifunctional enzyme subunit beta, mitochondrial  Mitochondrial import inner membrane translocase subunit TIM44  NADH dehydrogenase [ubiquinone] 1 beta subcomplex subunit 9  Isovaleryl-CoA dehydrogenase, mitochondrial  Methylmalonyl-CoA mutase, mitochondrial  Regulator of microtubule dynamics protein 1  Nicotinamide phosphoribosyltransferase  Reticulon-4-interacting protein 1, mitochondrial  Methylmalonate-semialdehyde dehydrogenase [acylating], mitochondrial  2-oxoisovalerate dehydrogenase subunit alpha, mitochondrial  2,4-dienoyl-CoA reductase [(3E)-enoyl-CoA-producing], mitochondrial  Sodium/calcium exchanger 1  Catenin alpha-3  Short-chain specific acyl-CoA dehydrogenase, mitochondrial  Carnitine O-palmitoyltransferase 2, mitochondrial  Ferrochelatase, mitochondrial  Ryanodine receptor 2  Delta(3,5)-Delta(2,4)-dienoyl-CoA isomerase, mitochondrial  NADH dehydrogenase [ubiquinone] 1 alpha subcomplex assembly factor 3  Enoyl-CoA hydratase domain-containing protein 2, mitochondrial  Succinate-semialdehyde dehydrogenase, mitochondrial  Metaxin-1  Immunity-related GTPase family M protein 1  Very long-chain specific acyl-CoA dehydrogenase, mitochondrial  Mitochondrial carnitine/acylcarnitine carrier protein  Mitochondrial pyruvate carrier 2  40S ribosomal protein S18  ADP-ribose glycohydrolase MACROD1  Mitochondrial pyruvate carrier 1  Myotilin  Mitochondrial import inner membrane translocase subunit Tim13  Reticulon-2  Amine oxidase [flavin-containing] B  Maleylacetoacetate isomerase  Mitochondrial peptide methionine sulfoxide reductase  Methylmalonyl-CoA epimerase, mitochondrial  Isochorismatase domain-containing protein 2A  D-beta-hydroxybutyrate dehydrogenase, mitochondrial  Acyl-coenzyme A thioesterase 2, mitochondrial  Fumarylacetoacetate hydrolase domain-containing protein 2A  Lipoamide acyltransferase component of branched-chain alpha-keto acid dehydrogenase complex, mitochondrial  Hydroxysteroid dehydrogenase-like protein 2  H-2 class I histocompatibility antigen, L-D alpha chain  Isoaspartyl peptidase/L-asparaginase  D-glutamate cyclase, mitochondrial  Gap junction alpha-1 protein  Ubiquinone biosynthesis monooxygenase COQ6, mitochondrial  Glutathione S-transferase kappa 1  Methylglutaconyl-CoA hydratase, mitochondrial  Kynurenine--oxoglutarate transaminase 3  Mitochondrial fission process protein 1 | 27  7.15  7  6.5  6  5.91  5.87  5.81  5.67  5.67  5.5  5.22  5.21  5  4.96  4.82  4.34  4.1  4.07  4  3.88  3.85  3.79  3.78  3.7  3.69  3.5  3.44  3.38  3.34  3.34  3.33  3.33  3.33  3.33  3.33  3.3  3.25  3.25  3.25  3.17  3.02  3  2.99  2.93  2.92  2.76  2.75  2.75  2.74  2.73  2.72  2.71  2.69  2.67  2.65  2.62  2.54  2.54  2.53  2.53  2.51  2.45  2.42  2.39  2.35  2.34  2.32  2.31  2.31  2.3  2.29  2.28  2.24  2.21  2.2  2.2  2.2  2.19  2.19  2.19  2.19  2.17  2.17  2.17  2.17  2.17  2.17  2.14  2.14  2.13  2.12  2.12  2.11  2.11  2.08  2.07  2.04  2.03  2.03  2  2  2  2  2  1.97  1.95  1.93  1.9  1.88  1.88  1.88  1.87  1.87  1.86  1.84  1.83  1.82  1.81  1.8  1.79  1.78  1.78  1.77  1.76  1.76  1.74  1.73  1.71  1.7  1.68  1.67  1.66  1.66  1.65  1.64  1.64  1.63  1.62  1.6  1.6  1.59  1.58  1.57  1.57  1.57  1.55  1.55  1.54  1.54  1.53  1.52  1.52  1.52  1.52  1.51  1.51  1.51  1.5  1.49  1.49  1.48  1.45  1.41  1.41  1.39  1.39  1.38  1.38  1.36  1.35  1.35  1.33  1.31  1.31  1.31  1.31  1.28  1.27  0.83  0.74  0.73  0.73  0.73  0.73  0.72  0.72  0.72  0.72  0.71  0.71  0.71  0.7  0.69  0.69  0.69  0.68  0.68  0.67  0.66  0.65  0.65  0.65  0.65  0.64  0.64  0.64  0.63  0.63  0.63  0.62  0.62  0.62  0.61  0.61  0.61  0.6  0.59  0.59  0.59  0.59  0.58  0.58  0.57  0.56  0.54  0.54  0.54  0.54  0.53  0.53  0.52  0.51  0.51  0.5  0.5  0.49  0.49  0.48  0.47  0.47  0.46  0.46  0.46  0.46  0.45  0.45  0.45  0.44  0.43  0.43  0.42  0.42  0.41  0.4  0.39  0.36  0.35  0.34  0.34  0.34  0.34  0.34  0.33  0.31  0.31  0.3  0.29  0.28  0.28  0.27  0.26  0.26  0.21  0.17 | Actin cytoskeleton organization  Osteoblast-driven collagen biomineralization  Stabilization of the structure of endoplasmic reticulum  Endothelial cell activation/negative regulation of the expression of cardiac genes  Epithelial cell differentiation  Collagen fiber assembly  Cellular detoxification of aldehydes  Inhibition of phospholipase A2/anti- coagulant activity  Desaturation and elongation of fatty acids/cholesterol biosynthesis  Hydrolysis of long-chain acyl-CoAs to the free fatty acid and coenzyme A (CoASH)  Mediation in the attachment, migration and organization of cells into tissues  Processing of ubiquitin precursors and of ubiquitinated proteins  Ubiquitin-dependent protein catabolic process  Regulation of cell migration through association with the actin cytoskeleton  Involvement in protein activation cascades  Regulation of macrophage function  Connection of major cytoskeletal structures to the plasma membrane  Negative regulation of mitochondrial fusion  Negative regulation of endopeptidase activity  Mediation in the signal transduction/ regulation of extrinsic apoptotic signalling  Cytoskeleton organization/signal transduction/cell differentiation  Regulation of endothelial cell migration/ protein refolding  Membrane reorganization/tubulation upon ATP hydrolysis/early endosomal transport  Regulation of cardiac repair after injury  Epithelial cell differentiation/actin cross-linking protein  Modulating actin-based shape  Regulation of translational initiation  Epithelial cell polarization  Desaturation and elongation of fatty acids/cholesterol biosynthesis  Chaperone-mediated protein folding  Chaperone-mediated protein folding/actin organization  Protein N-linked glycosylation  Actin filament organization  Creation a link between the actin cytoskeleton and the membrane  Cytoskeleton-dependent cytokinesis  Anchoring of basement membranes to the underlying connective tissue  ATP-dependent degradation of ubiquitinated proteins  Complement activation/blood coagulation  Protein ubiquitination/receptor internalization and degradation in lysosomes  Actomyosin structure organization  Redox homeostasis  Regulation of Ca^(2+)^ homeostatis  Resealing of membranes disrupted by mechanical stress  Biosynthetic pathway of collagen  Proteasome-mediated ubiquitin-dependent protein catabolic process  Integrin-mediated signal transduction  Vitamin D transport and storage  Regulation of heart contraction  Chaperone-mediated protein folding  Iron transport across the cell membrane  Cytoplasmic translation  Ubiquitin-dependent protein catabolic process  Canonical glycolysis  Endocytosis/intracellular protein transport  AP2-dependent clathrin-mediated endocytosis  Protein folding  Anchoring of various transmembrane proteins to the actin cytoskeleton  Iron ion transport  Actin filament depolymerization  Regulation of myoblast differentiation/ proliferation  Inhibition of aggregation of misfolded proteins/platelet aggregation and activation  Intermediate filament organization  A component of microfibrils found extracellularly in connective tissues  Stabilization of microvessels and muscle cells, both in heart and in skeletal muscle  Promoting of folding, oligomeric assembly and quality control in the endoplasmic reticulum  Processing and transport of secreted proteins  Myoblast fusion  Nucleosome assembly  Formation, breakage and rearrangement of disulfide bonds  Glyoxylate metabolic process  Cytokinesis/regulation of cell shape  Cytoskeleton reorganization/cytokinesis/ regulation of cell shape Controlling of mitochondrial reactive oxygen species homeostasis/apoptosis regulation and cell viability  Regulation of intracellular concentrations of taurine and glutamate  Modulating the activity of the immune system  Protein catabolic process  ATP-dependent degradation of ubiquitinated proteins  Mediating in mitochondrial clustering and fusion  Cell adhesion  ITP catabolic process  Modulation of mRNA transcription by Polymerase II/RNA transport  Cleavage of cyclic esters  Folding of proteins upon ATP hydrolysis  Involved in proteolytic events essential for cell growth and viability  Formation of translation preinitiation complex  Pre-mRNA splicing/mRNA turnover  Blood coagulation/ inflammation/wound healing  Cell adhesion/cell motility/wound healing  Actin cytoskeleton organization  Chromosome segregation  Cell adhesion  Proteasome-mediated ubiquitin-dependent protein catabolic process  Organization of actin filament bundles  Microtubule cytoskeleton organization  ATP-dependent degradation of ubiquitinated proteins  Early autophagosome formation  Regulation of collagen fibrillogenesis in connective tissues  Protein methylation  Promoting of membrane fusion/exocytosis  Exocytosis of intracellular proteins  Microtubule cytoskeleton organization  Intracellular retrograde motility of vesicles and organelles along microtubules  Alternative mRNA splicing, via spliceosome  Controlling of Rho proteins homeostasis/Rho protein signal transduction  Inhibition of calpain (calcium-dependent cysteine protease)/muscle protein degradation  Folding of proteins upon ATP hydrolysis  Modulation of various transmembrane signalling systems  Cytoskeleton-dependent cytokinesis  Promoting of sarcomere formation in cardiomyocytes and increasing of cardiomyocyte contractility  cAMP-mediated signalling  Anchoring of microtubules to centrosomes Degradation of misfolded, unassembled or oxidatively damaged polypeptides in the mitochondrial matrix  Fragmentation of Golgi stacks during mitosis/their reassembly after mitosis  Assembling of signalling complexes (i.e. ABL kinases) in actin cytoskeleton  Intermediate filament organization  Canonical glycolysis  Anchoring of various transmembrane proteins to the actin cytoskeleton  Endocytosis  Nuclear assembly/chromatin organization/nuclear membrane and telomere dynamics’ regulation  Protein refolding  Vesicle-mediated protein transport  Cardiac muscle contraction/cardiac muscle hypertrophy in response to stress  Lactate biosynthetic process from pyruvate  Muscle contraction  Negative regulation of endopeptidase activity  Canonical glycolysis  Translation/cell adhesion to the basement membrane  Biosynthesis of glycogen  An adapter bringing other proteins (like kinases) to the cytoskeleton  Proteasome-mediated ubiquitin-dependent protein catabolic process  Accelerating of the folding of proteins  Connection of major cytoskeletal structures to the plasma membrane  Regulation of a signal transduction  Cellular iron ion homeostasis  Microtubule cytoskeleton organization  Translational elongation  Integrin-mediated signalling  Attenuating of integrin beta-1 activation at the cell surface  Reorganizing of the actin cytoskeleton in response to signalling events  Cell adhesion  Cardiac muscle contraction  Chaperone-mediated protein folding  'De novo' AMP/IMP/XMP biosynthetic process/GMP biosynthesis/purine nucleobase biosynthesis  Anchoring of actin to a variety of intracellular structures  Cytoplasmic translation  Microtubule cytoskeleton organization  Cellular iron ion homeostasis  Regulation of stress fiber assembly  Negative regulation of blood coagulation  Proteasome-mediated ubiquitin-dependent protein catabolic process  Translational elongation  Protein ubiquitination/ubiquitin-dependent protein catabolic process  Canonical glycolysis  Cell-cell adhesion  Cell motility  Anchoring of actin to a variety of intracellular structures  Promoting of the development of heart hypertrophy  Modulation of the rate of fibrils formation  Actomyosin structure organization  Pre-rRNA transcription/ribosome assembly  Canonical glycolysis  Folding of proteins/degradation of misfolded proteins  Chaperone-mediated protein complex assembly  Myocardium sarcomere organization  Cellular detoxification of nitrogen compound  mRNA splicing/mRNA transport  Chaperone-mediated protein folding  Anchoring of intermediate filaments to the desmosomes  Regulation of apoptosis  Microtubule cytoskeleton organization  Detoxification of formaldehyde  Controlling of methylations/regulation of the intracellular concentration of adenosylhomocysteine  Protein ubiquitination/SCF complex assembly  Actin filament organization  Canonical glycolysis  Cross-linking and stabilization of cytoskeletal intermediate filaments network/regulation of its dynamics  Cell-matrix adhesion/cell-cell adhesion  Chaperone-mediated protein complex assembly  Microtubule cytoskeleton organization  Acetyl-CoA biosynthetic process from acetate  Biosynthesis of coenzyme Q  Regulation of apoptosis  Fatty acid beta-oxidation  Phosphocreatine biosynthetic process  Cholesterol homeostasis  Polyol pathway of sugar metabolism  Transfer of electrons from NADH to the respiratory chain  Tricarboxylic acid cycle  Cell redox homeostasis  Tricarboxylic acid cycle  Catabolism of odd chain fatty acids and branched-chain amino acids (isoleucine, valine)  Modulation of calcineurin signalling  Hydrogen sulfide biosynthetic process  Long-chain fatty acid catabolic process  Synthesis of short- and branched-chain acylcarnitines/metabolism of short(medium)-chain fatty acids  Long-chain fatty acid metabolic process  Tricarboxylic acid cycle  Long-chain fatty acid transport  Transfer of electrons from NADH to the respiratory chain  Medium-chain fatty acid catabolic process  Catabolism of odd chain fatty acids, branched-chain amino acids (isoleucine, valine)  Fatty acid beta-oxidation  Cytoskeleton organization  Exchange of sodium and potassium ions across the plasma membrane  Phosphate-containing compound metabolic process  Sensing of reactive xenobiotics in the cytoplasm/intra-Golgi protein transport  Regulating of cardiac sodium current  Transfer of electrons from NADH to the respiratory chain  Mediating in protein-protein interactions  Hydrolysis of triglycerides of circulating chylomicrons and very low density lipoproteins (VLDL)  Electron transport chain/Accepting of electrons from ETF and reducing of ubiquinone  Histone lysine methylation  Fatty acid metabolic process  Protein de-ADP-ribosylation  Leucine catabolic process  Branched-chain amino acid catabolic process  Regulation of mitochondrial respiration and mitochondrial DNA copy number maintenance  Mitochondrial electron transport, NADH to ubiquinone  Fatty acid beta-oxidation  Ketone body catabolic process  Exchange of Na(+) and K(+) ions across the plasma membrane  Iron homeostasis regulation/protecting cells against apoptosis  Cholesterol ester hydrolysis in macrophages  ATPase activity  Fatty acid beta-oxidation  Protein import into mitochondrial matrix  Mitochondrial electron transport, NADH to ubiquinone  Leucine catabolic process Isomerization of methylmalonyl-CoA to succinyl-CoA/linking the catabolic pathway of BCAAs to the Krebs cycle  Microtubule binding activity  NAD biosynthesis  Coenzyme Q (CoQ) biosynthesis/supporting oxidative phosphorylation activity  Valine catabolic process  Branched-chain amino acid catabolic process  Fatty acid beta-oxidation  Regulation of cytoplasmic Ca(2+) levels and Ca(2+)-dependent cellular processes  Formation of stretch-resistant cell- cell adhesion complexes  Short-chain fatty acid beta-oxidation/butyrate catabolic process  Long-chain fatty acid transport/metabolism  Heme biosynthesis  Cellular calcium ion homeostasis  Fatty acid beta-oxidation  Mitochondrial respiratory chain complex I assembly  Fatty acid beta-oxidation  Gamma-aminobutyric acid catabolic process  Transport of proteins into the mitochondrion  Regulation of autophagy  Fatty acid beta-oxidation  Transport of acylcarnitines of different length across the mitochondrial inner membrane  Mitochondrial pyruvate transmembrane transport  Cytoplasmic translation  Protein de-ADP-ribosylation  Mitochondrial pyruvate transmembrane transport  Controlling of myofibril assembly and stability  Protein insertion into mitochondrial inner membrane  Intracellular protein transmembrane transport  Oxidative deamination of biogenic and xenobiotic amines  Degradation of phenylalanine/tyrosine  Repairing of proteins that have been inactivated by oxidation  Short-chain fatty acid catabolic process/degradation of branched chain amino acids (valine, isoleucine)  Protein destabilization  Ketone body catabolic process  Hydrolysis of acyl-CoAs to the free fatty acid and coenzyme A (CoASH)  Hydrolase activity/tyrosine catabolism  Branched-chain amino acid catabolic process  Lipid metabolism regulator  Defence response/immune response  Asparagine catabolic process via L-aspartate  Conversion of D-glutamate to 5-oxo-D-proline  Regulation of synchronized contraction of the heart  Ubiquinone biosynthesis  Protection against oxidative stress  Detoxification of itaconate  L-kynurenine catabolic process  Mitochondrial fission |

275 statistically significant proteins out of 1162 included in the analysis (179 up-regulated, 96 down-regulated). Proteins related to metabolism are marked in purple. Note: some of the entities were slightly above/below FC threshold but were retained in the dataset due to biological importance.

**Sup. Fig. 1.** *The number of proteins detected and quantified by proteomic mass spectrometry analysis using a label-free approach and an Orbitrap Exploris 480 mass spectrometer.* Results are expressed as mean ± SD (*n* = 5-7/group).


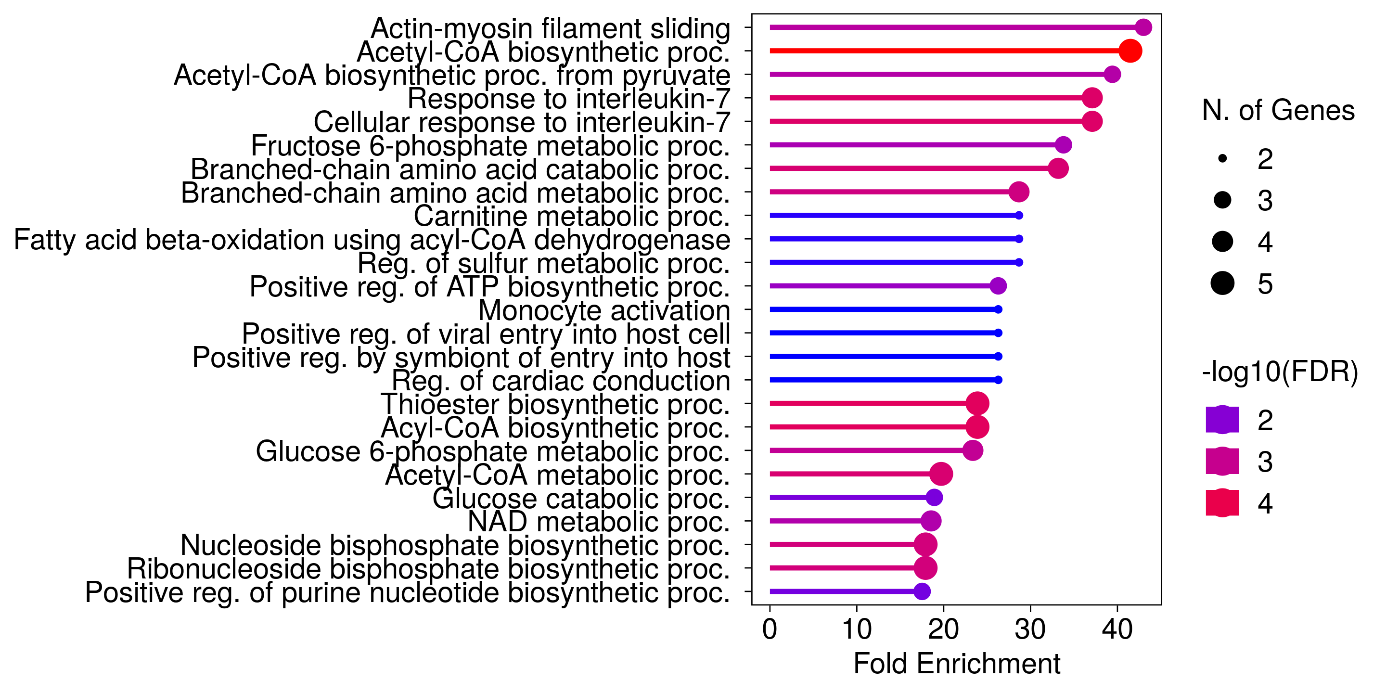


**A**


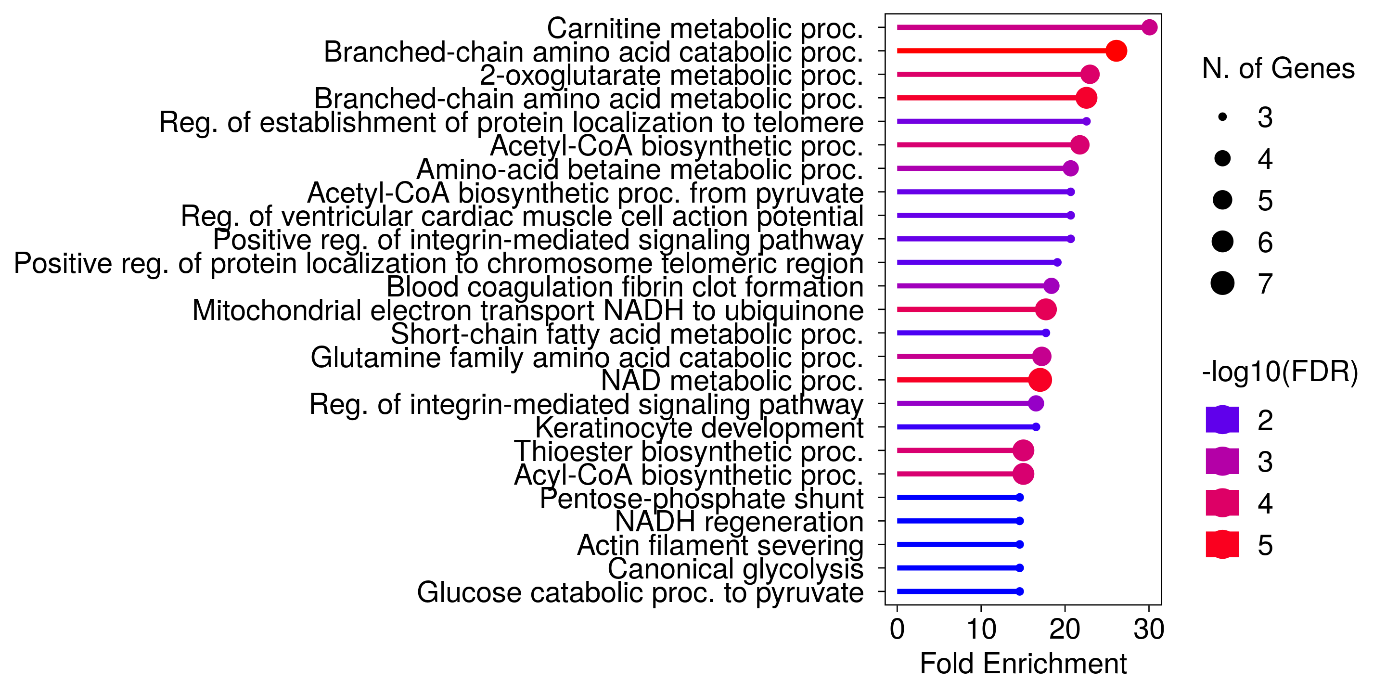


**C**

**B**


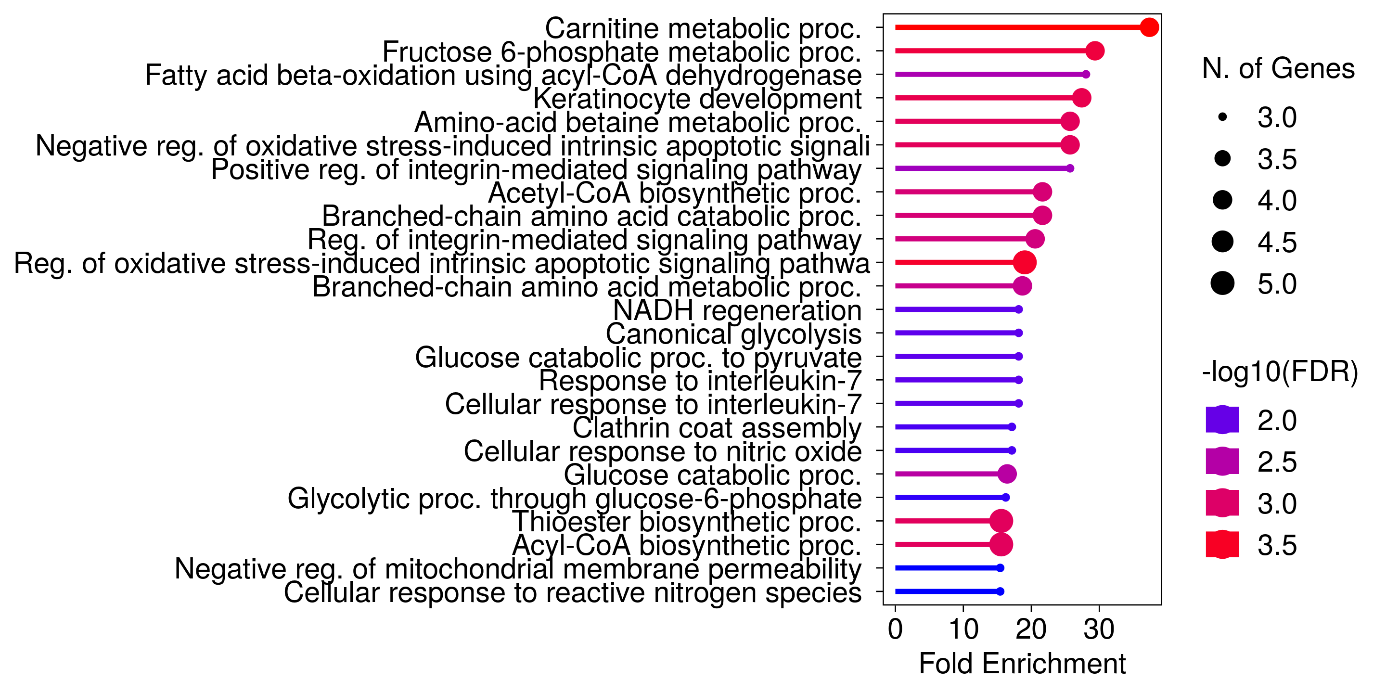


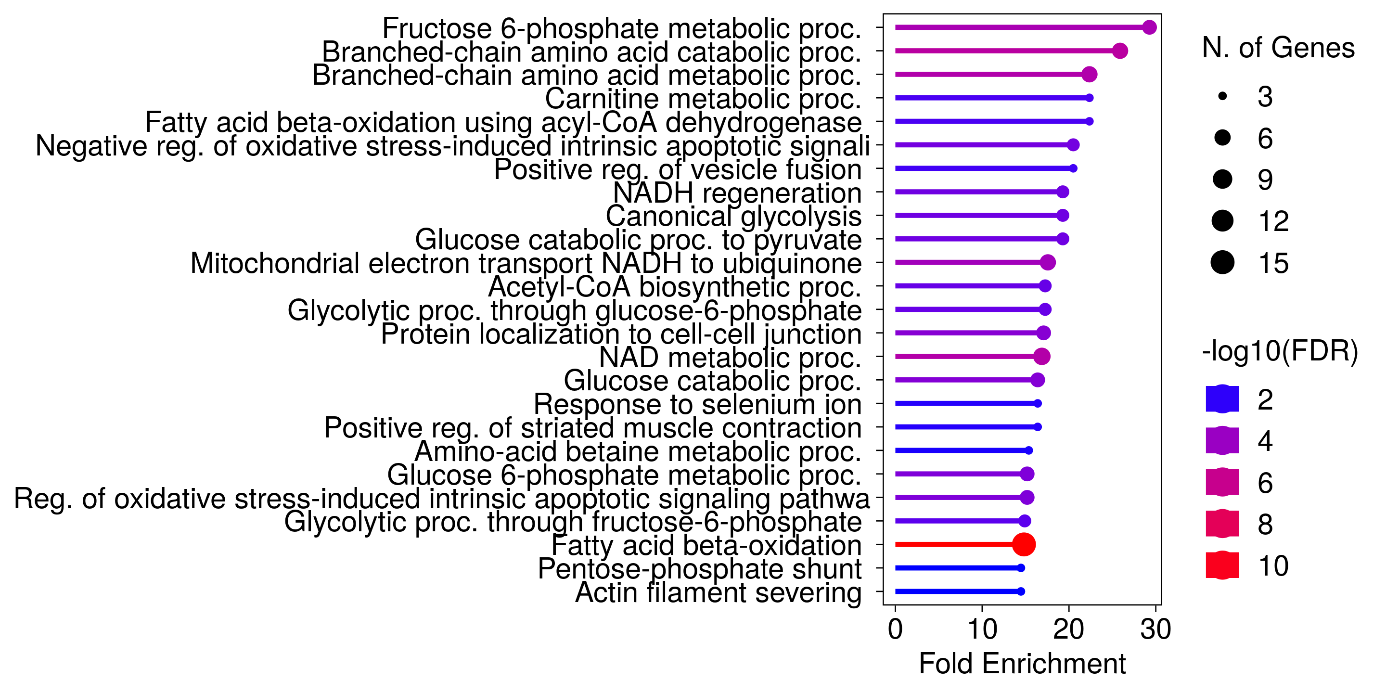


**E**

**D**


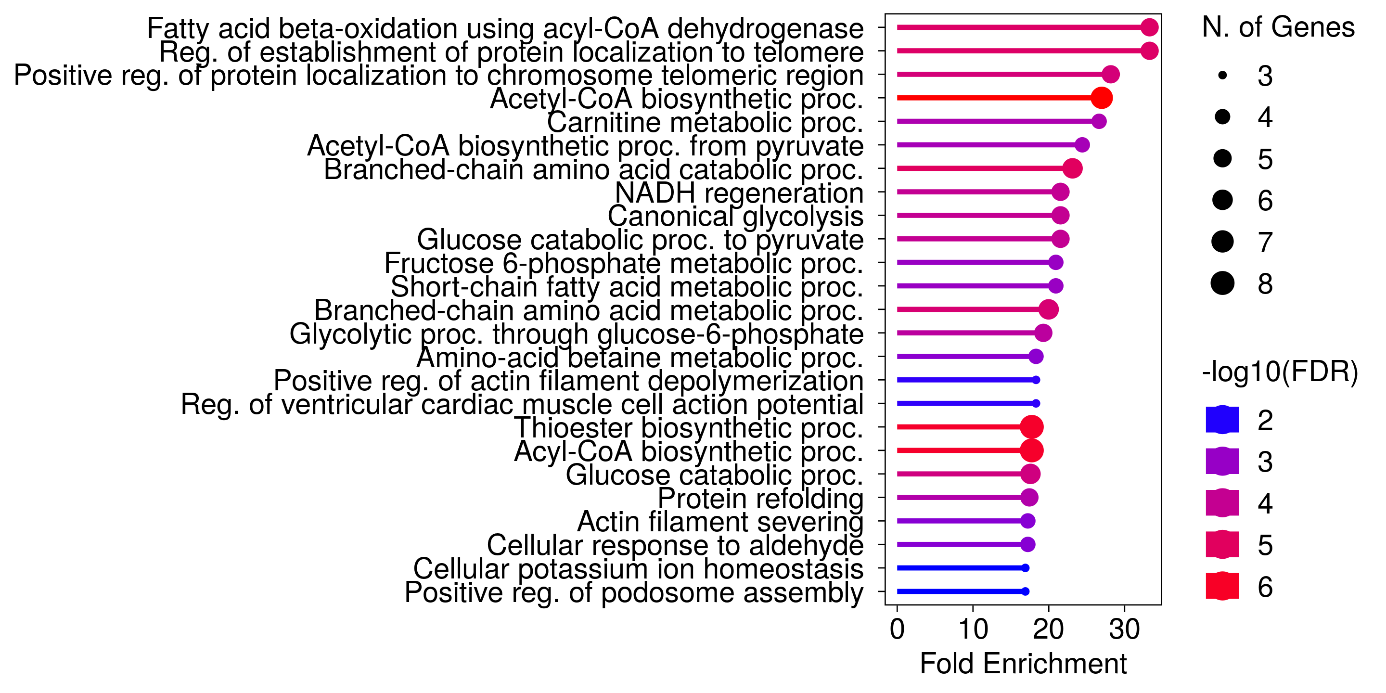


**Sup. Fig. 2.** *Protein Enrichment Analysis based on the number of proteins in each dysregulated pathway for (****A****) 4-, (****B****) 8-, (****C****) 10-, (****D****) 12-, and (****E****) 14-month-old animals.*

**Sup. Fig. 3.** *The repertoire of PDK isoforms in Tgαq*44 mice that were semi-quantified using the exponentially modified protein abundance index (emPAI).* Results are expressed as median and range. Median values are depicted above relevant box-and-whisker plots. PDK1, PDK2, PDK4, pyruvate dehydrogenase kinase isoform 1, 2 or 4; PDP1, pyruvate dehydrogenase phosphatase 1.

**FVB mice**

**Tgαq*44 mice**

**Sup. Fig. 4.** *Myocardial extraction of FFAs, leucine, valine, glutamate, β-hydroxybutyrate and carbohydrates under dobutamine (Dbn) stress in 4- and 14-month-old FVB and Tgαq*44 mice (n = 5−10/group) measured as transcardiac arteriovenous gradients of relevant metabolites.* Coronary effluent samples were collected immediately before and after injection of the bolus of Dbn. Results are expressed as median and range. Statistical significance was determined by a 2-way ANOVA with a Tukey post-hoc test; * *p* < 0.05, ** *p* < 0.01, *** *p* < 0.001, **** *p* < 0.0001.


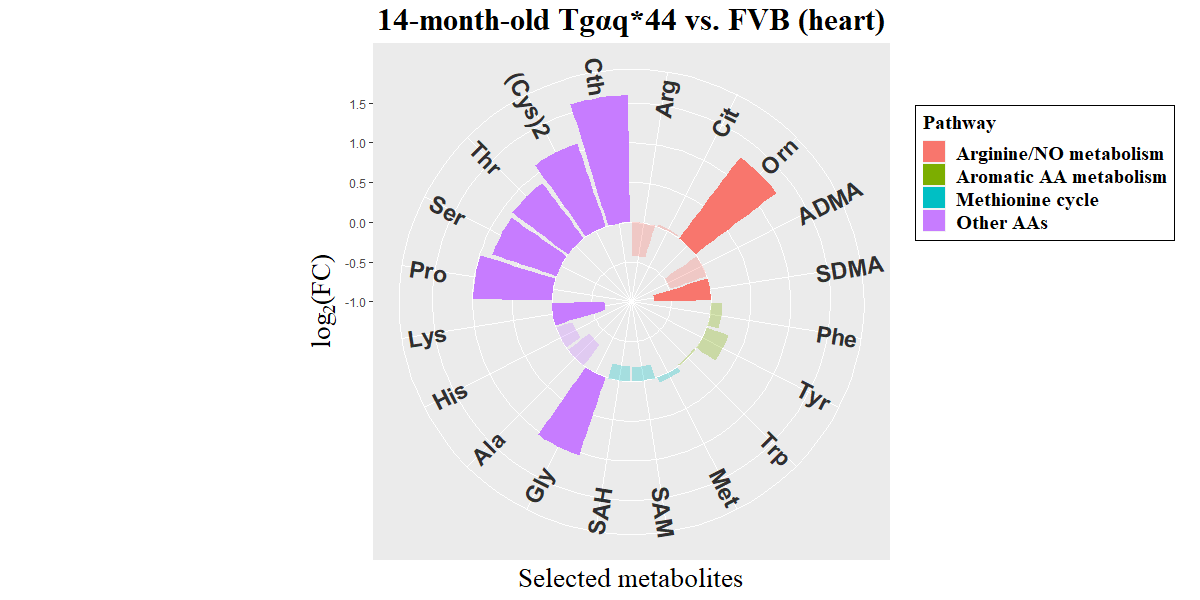

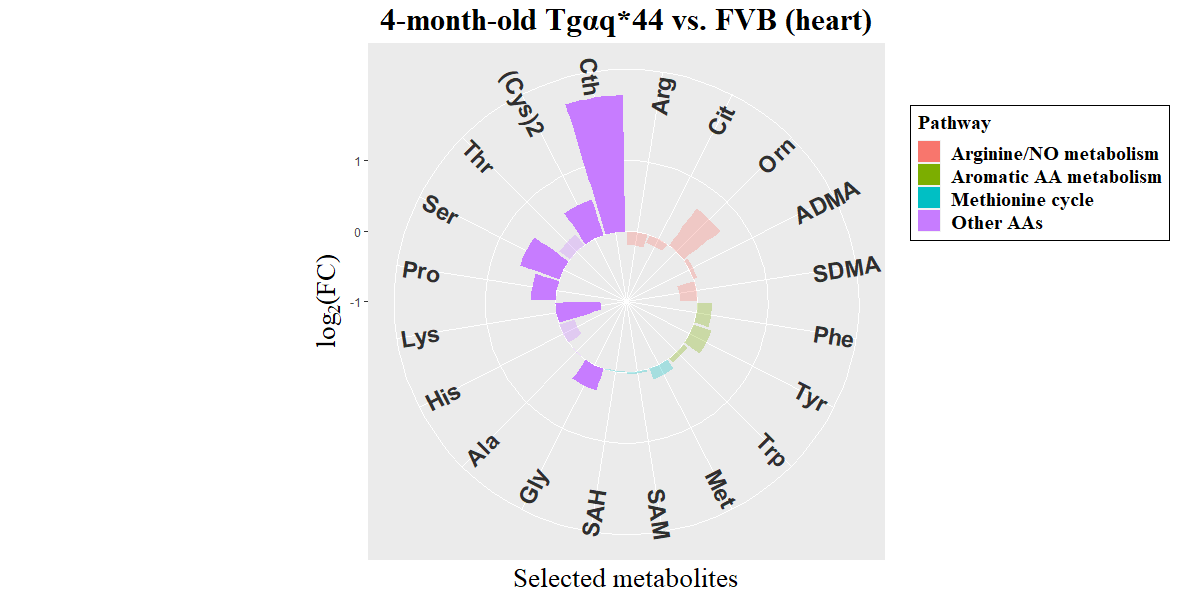

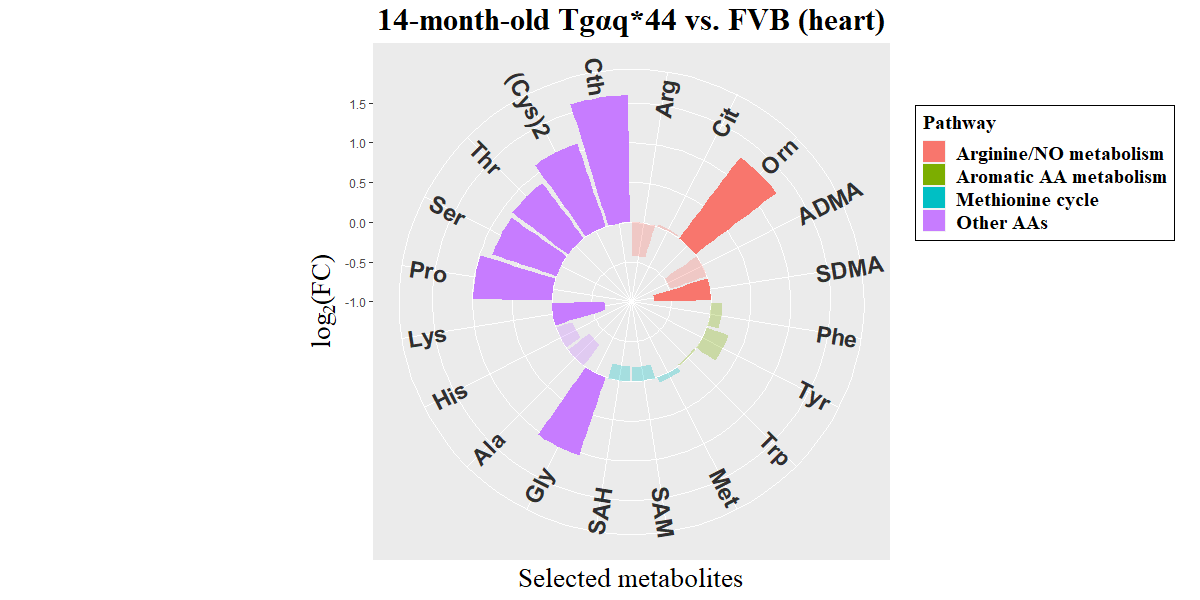


**Sup. Fig. 5.** *Myocardial metabolomic signatures in a mouse model of chronic HF.* Polar (flower) plots of cardiac metabolites in 4 investigated groups: 4- (*n* = 8) and 14-month-old (*n* = 5‒10) FVB and Tgαq*44 mice. The fold changes (Tgαq*44 /FVB) for each metabolite are shown by their distance from the polar plot origin. Statistical differences between each set of group comparisons are denoted by the intensity of the colour (dark colour – the change statistically meaningful, shaded – non-significant). Data were analysed by unpaired two-tailed Student’s t-test. Polar plots were generated using R (ver. 4.3.1) package ggplot2 (ver. 3.4.3). Abbreviations: AAs, amino acids; ADMA, asymmetric dimethylarginine; Ala, alanine; Arg, arginine; Cit, citrulline; Cth, cystathionine; (Cys)_2_, cystine; Gly, glycine; His, histidine; Lys, lysine; Met, methionine; NO, nitric oxide; Orn, ornithine; Phe, phenylalanine; Pro, proline; SAM, S-adenosylmethionine; SAH, S-adenosylhomocysteine; SDMA, symmetric dimethylarginine; Ser, serine; Thr, threonine; Trp, tryptophan; Tyr, tyrosine.


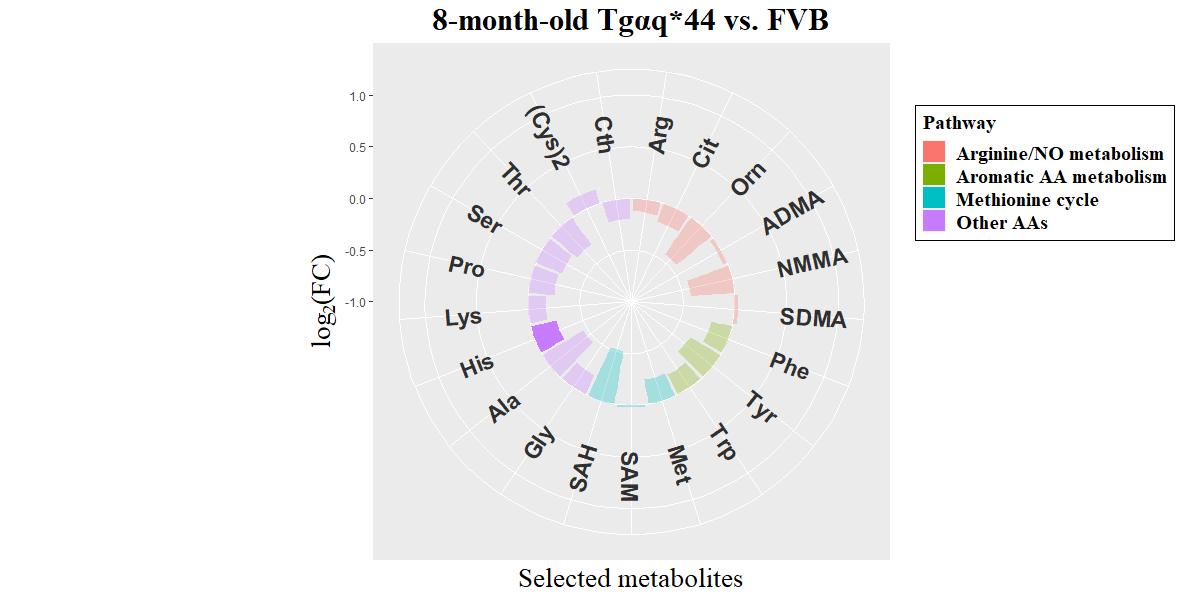

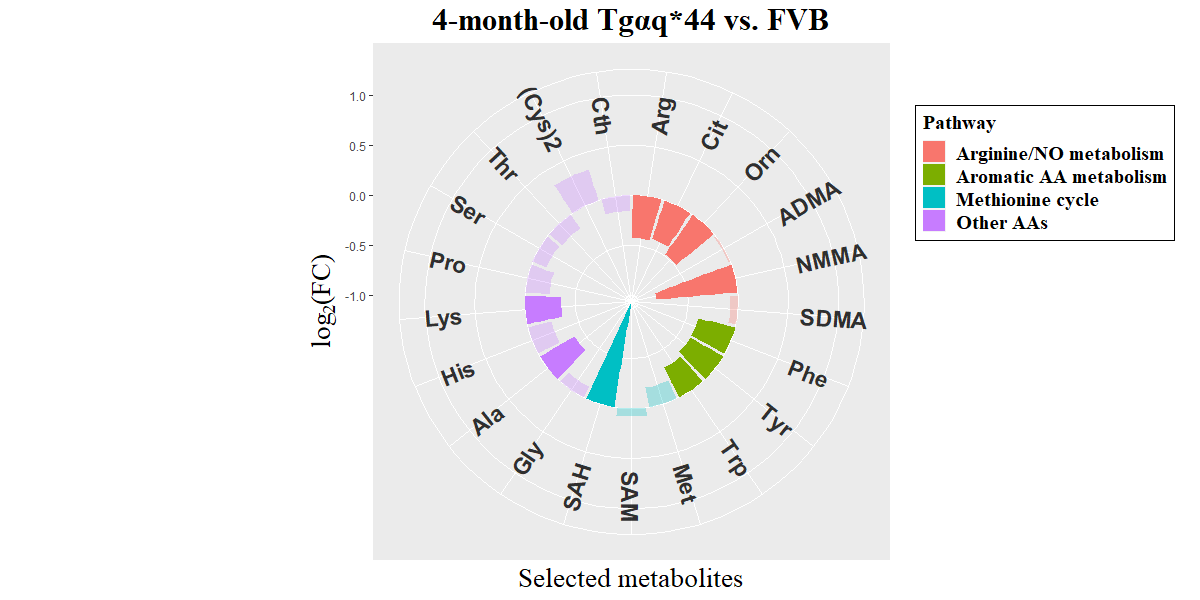

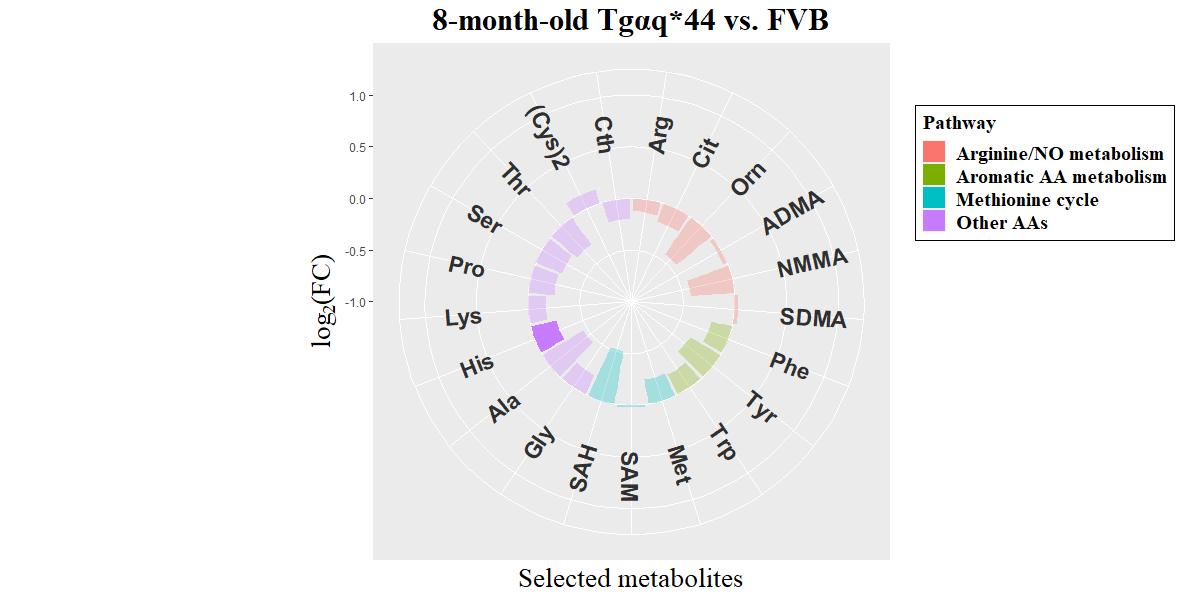


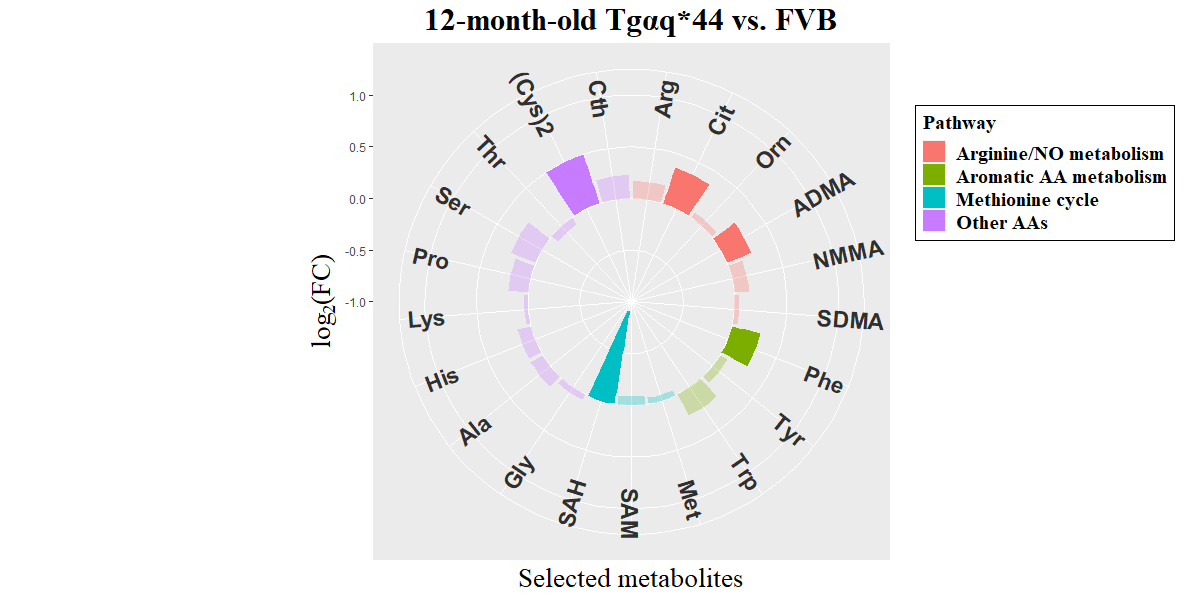

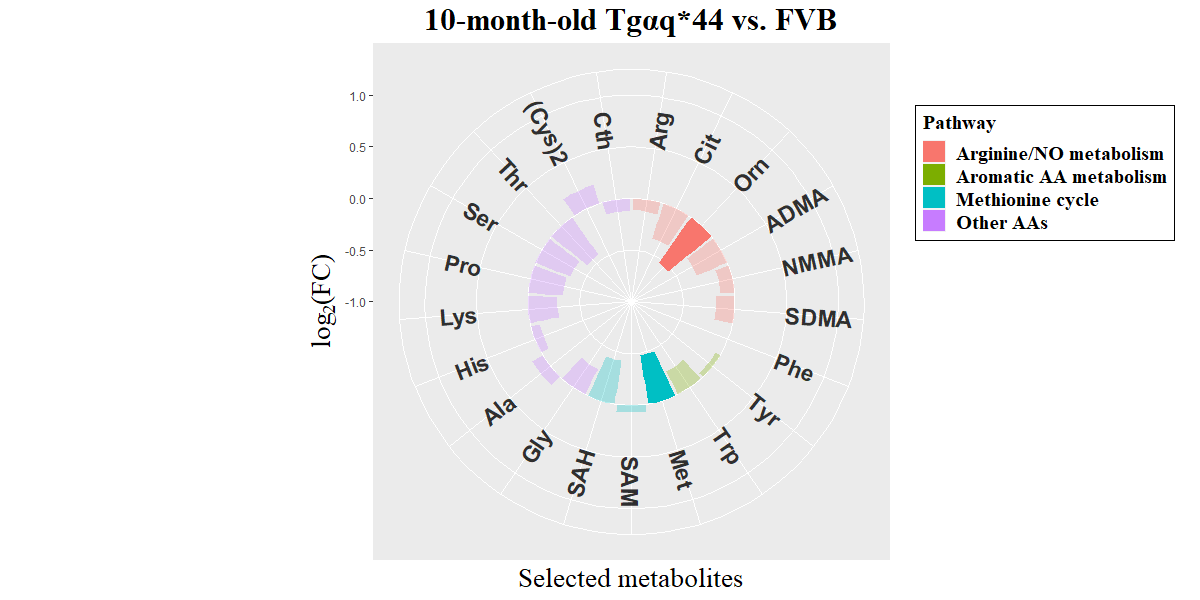

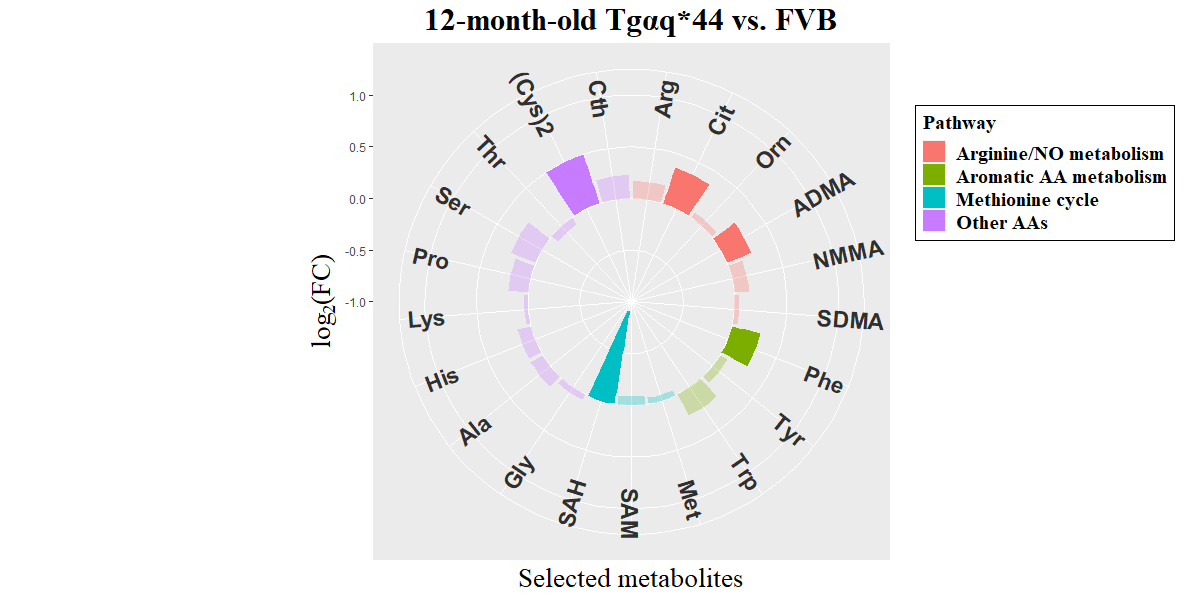


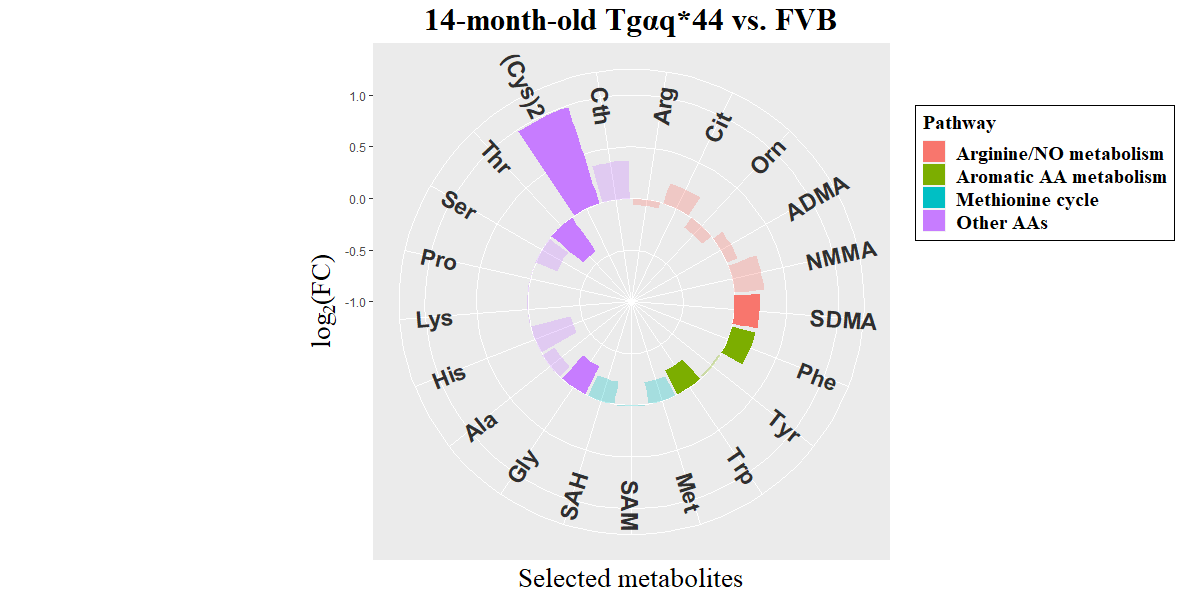

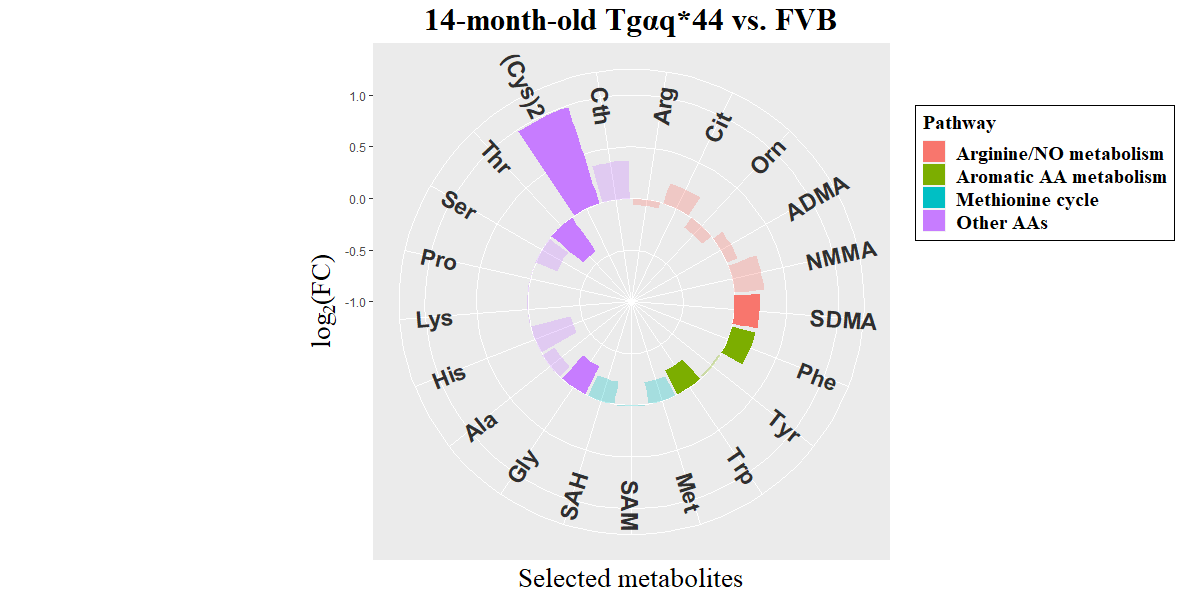

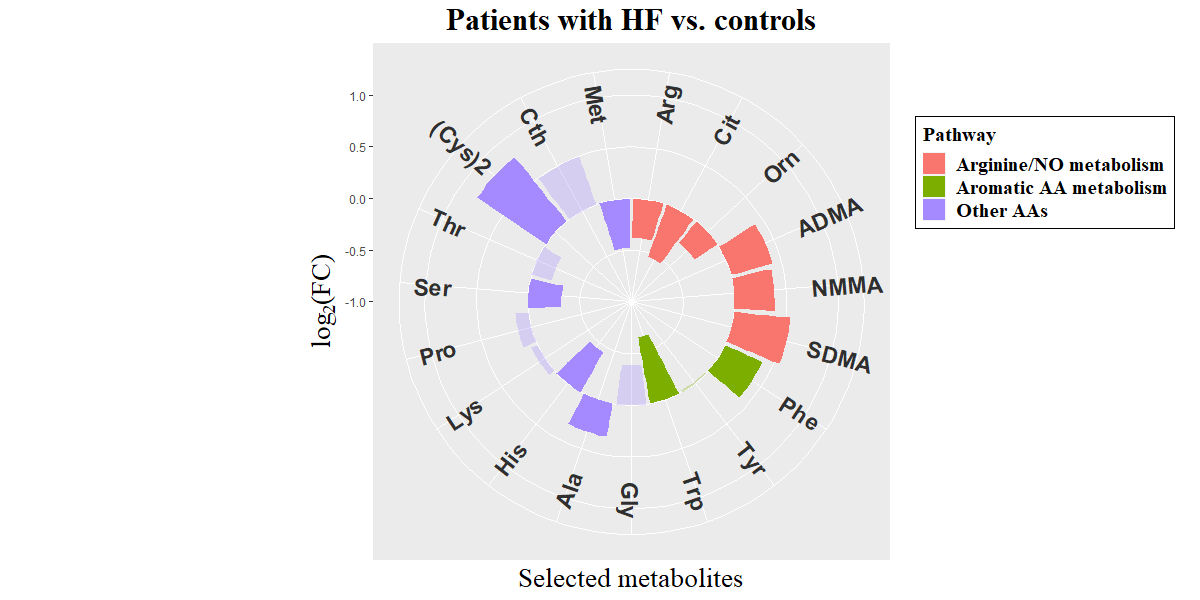


**Sup. Fig. 6.** *Plasma metabolomic signatures of cardiac remodelling in murine and human HF.* Polar (flower) plots of plasma metabolites in 10 investigated animal groups: 4-, 8-, 10-, 12- and 14-month-old FVB and Tgαq*44 mice (*n* = 7‒10/group) as well as in HF patients and control subjects (*n* = 18‒20/group). The fold changes (HF/Ctrl) for each metabolite are shown by their distance from the polar plot origin. Statistical differences between each set of group comparisons are denoted by the intensity of the colour (dark colour – the change statistically meaningful, shaded – non-significant). Data were analysed by unpaired two-tailed Student’s t-test. Polar plots were generated using R (ver. 4.3.1) package ggplot2 (ver. 3.4.3). Abbreviations: AAs, amino acids; ADMA, asymmetric dimethylarginine; Ala, alanine; Arg, arginine; Cit, citrulline; Cth, cystathionine; (Cys)_2_, cystine; Gly, glycine; His, histidine; Lys, lysine; Met, methionine; NMMA, N(G)-monomethyl-l-arginine; NO, nitric oxide; Orn, ornithine; Phe, phenylalanine; Pro, proline; SAM, S-adenosylmethionine; SAH, S-adenosylhomocysteine; SDMA, symmetric dimethylarginine; Ser, serine; Thr, threonine; Trp, tryptophan; Tyr, tyrosine.

**Sup. Fig. 7.** *The relationship between selected blood metabolites and LVEF in HF patients.* A strong relation between the dependent (LVEF) and the independent variables (metabolites) was found. LVEF, left ventricular ejection fraction; Ile, isoleucine; Leu, leucine; Val, valine; BCAA(s), branched-chain amino acid(s); Pyr, pyruvate; Lac, lactate; β-OHB, β-hydroxybutyrate; C16‒Car, palmitoylcarnitine.
